# Taxonomy of North European *Lumbricillus* (Clitellata, Enchytraeidae)

**DOI:** 10.3897/zookeys.703.13385

**Published:** 2017-09-27

**Authors:** Mårten J. Klinth, Emilia Rota, Christer Erséus

**Affiliations:** 1 Department of Biological & Environmental Sciences, University of Gothenburg, P.O. Box 463, SE-405 30 Gothenburg, Sweden; 2 Department of Physics, Earth and Environmental Sciences, University of Siena, Via P.A. Mattioli 4, IT-53100 Siena, Italy

**Keywords:** Annelida, Oligochaeta, *Lumbricillus latithecatus* sp. n., *Lumbricillus scandicus* sp. n., *Claparedrilus* gen. n., *Claparedrilus semifuscoides* sp. n., *Claparedrilus semifuscus* (Claparède, 1861) comb. nov.

## Abstract

*Lumbricillus* is a genus of clitellate worms with about 80 described species that inhabit marine and limnic habitats. This study follows a recent analysis of the phylogeny of the genus based on 24 species of *Lumbricillus* collected mainly in Norway and Sweden. We provide the illustrated taxonomic descriptions of all these species and describe two of them as new; *Lumbricillus latithecatus* **sp. n.** and *L. scandicus* **sp. n.** Using the recent phylogeny, we informally divide *Lumbricillus* into five distinct morphological groups, into which we also tentatively place the *Lumbricillus* species not included in this study. Furthermore, we establish *Claparedrilus* **gen. n.**, with the type species *C. semifuscoides* **sp. n.**, and transfer *Pachydrilus semifuscus* Claparède, 1861 (previously referred to *Lumbricillus*) into said genus.

## Introduction

Enchytraeids (Annelida, Clitellata, Enchytraeidae) are small clitellate worms that mainly inhabit terrestrial soils but the family is well represented in the aquatic environment. One of the about 30 genera, *Lumbricillus* Ørsted, 1844, is primarily found in marine and freshwater habitats, but also in humid soils (Nielsen and Christensen 1959). It was established by Ørsted (1844), and *Lumbricus lineatus* Müller, 1774, the first enchytraeid ever described (Michaelsen 1889), was later regarded as its type species (Brinkhurst and Jamieson 1971; Coates and Ellis 1981). Ørsted (1842) had earlier referred *Lumbricus lineatus* to one of three groups constituting the “Lumbricillae”, and specifically to the group distinguished by having short, almost straight chaetae, “resembling stitching awls”, in both upper and lower bundles. He would later (Ørsted 1844) name this group *Lumbricillus*. Today, after several emendations, the genus is conceived as having straight to sigmoid chaetae, nephridia with short anteseptale consisting of nephrostome only, and testes enclosed in lobed peritoneal sacs (testis sacs) which, for most species, are structured as regular bunches (Nielsen and Christensen 1959).

Returning to the 19^th^ Century, Claparède described his *Pachydrilus* Claparède, 1861 as an assemblage of marine littoral species lacking “hair bristles”, but having a single pair of spermathecae in segment V, clitellum covering segments XI–XIII, male pores in XII, and simple vascular and nervous systems. *Pachydrilus* (and its five representatives) shared these basic traits with the terrestrial species then classified in *Enchytraeus* Henle, 1837, but was distinguished by the lack of dorsal pores and by generally possessing red-colored blood. *Pachydrilus* was later redefined by Vejdovský (1879) to include only species with sigmoid chaetae and small nephridial anteseptals and then further restricted by Roule (1888, 1889) and Michaelsen (1888, 1889), with a diagnosis focusing on the possession of multilobed testes, which led to the selection of *P. verrucosus* Claparède, 1861 as the type species. It must be mentioned that the very same species epithet had been used by Ørsted in 1844 for his *Lumbricillus verrucosus*, but due to the lack of an adequate description, it was considered a *nomen nudum* and *incertae sedis* (both Vejdovský 1884:45 and Michaelsen 1900:51 believed it should be placed among Tubificidae). The present study will treat *P. verrucosus* Claparède, 1861 as a valid enchytraeid species.

**Figure 1.**
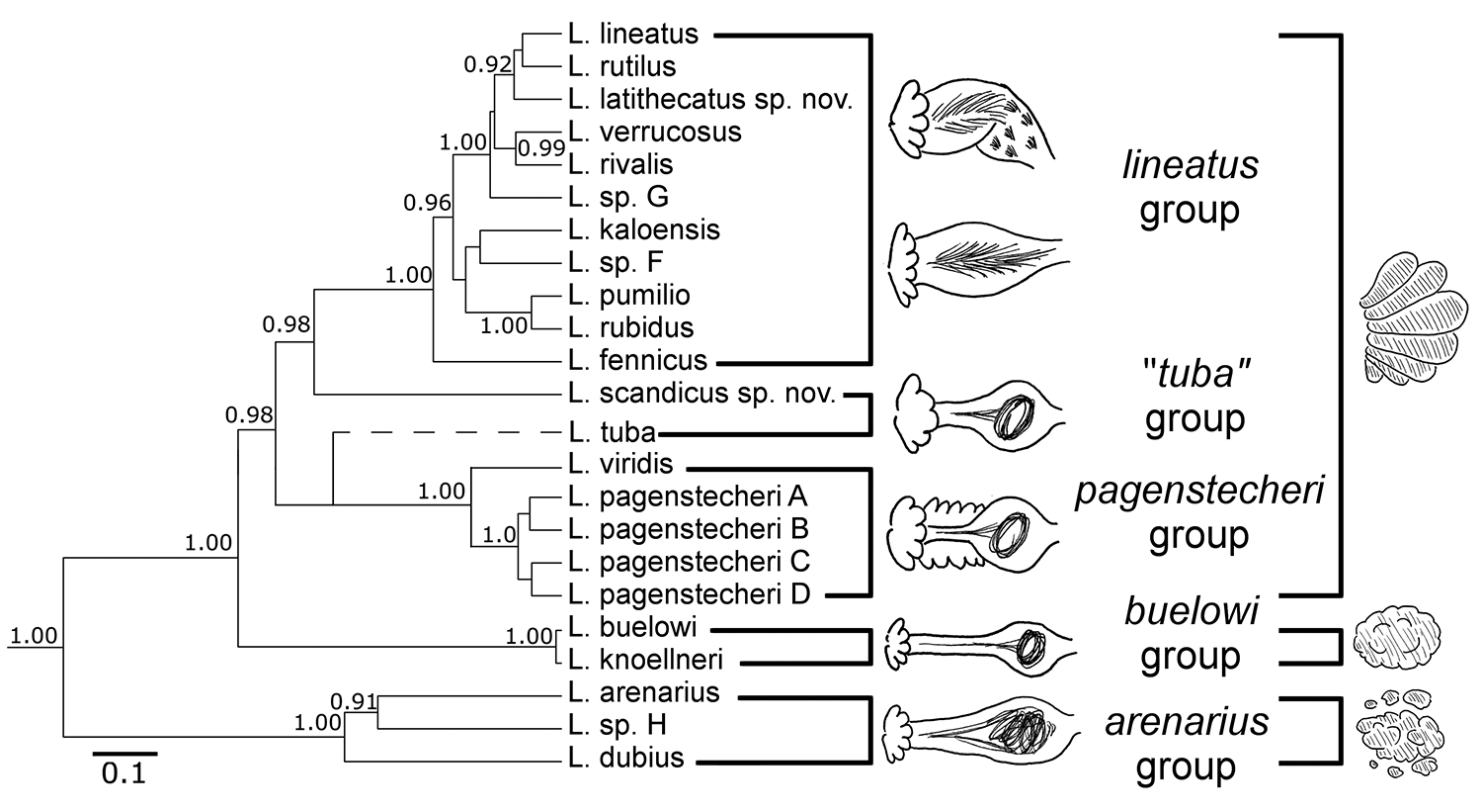
Phylogeny of North European *Lumbricillus*, modified from Klinth et al. (2017). Species tree based on 12S, 16S, COI, 18S, 28S, ITS and H3 genes, estimated using Bayesian inference under the multispecies coalescent model in *BEAST. Posterior probabilities higher than 0.9 shown as support values. Scale shows expected number of changes per site in COI with all other genes relative to it. *Lumbricillus tuba* has been added to the tree at the most probable position given the other gene and species trees from Klinth et al. (2017). The tree depicts the five morpho-groups that are used in the present study to discuss the relationships within *Lumbricillus*. The general morphology of the spermathecae and testes/testis sacs of these groups are also shown.

The name *Pachydrilus* soon became a competitor for *Lumbricillus* in the taxonomic literature. Some scientists favored *Pachydrilus* (Michaelsen 1888, 1889; Ude 1929; Černosvitov 1937), others *Lumbricillus* (Eisen 1904; Southern 1909; Stephenson 1911, 1930). In 1900, Michaelsen attempted to resolve the conflict by accepting the seniority of the name *Lumbricillus* and placing *Pachydrilus verrucosus* within this genus (Michaelsen 1900) and proceeded to use this name in following publications (Michaelsen 1905, 1911). However, he later went back to using *Pachydrilus* instead of *Lumbricillus* (Michaelsen 1925, 1929, 1934, 1935; see Rota et al. 2008). The use of two competing names ended in 1959 when Nielsen and Christensen synonymized *P. verrucosus* with *L. lineatus*, directly rendering *Pachydrilus* a junior synonym of *Lumbricillus*; *P. verrucosus* had previously been suggested as a form of *L. lineatus* by Ude (1929) and Černosvitov (1937). *Lumbricillus verrucosus* has recently been resurrected as a separate species with molecular support (Klinth et al. 2017) and will be given a more extensive morphological description in this paper. It is important to note that by re-instating *L. verrucosus* as a valid species, we do not change the status of *Pachydrilus* as a junior synonym to *Lumbricillus*, as *L. verrucosus* and *L. lineatus*, using genetic data, have been found to be closely related to each other (Klinth et al. 2017; see also Fig. 1 herein).

In 1885, Saint-Loup described the species *Pachydrilus enchytraeoides* Saint-Loup, 1885 from the Marseille harbor. The rather brief description mentioned pouch-like spermathecae, a simple circulatory system and irregularly lobed testes (Saint-Loup 1885). The combination of these characters, interpreted by Saint-Loup’s fellow researcher Roule to be intermediate between *Lumbricillus* and *Enchytraeus*, convinced Roule (1888) to transfer the species to a new genus, which he named *Enchytraeoides* Roule, 1888 in acknowledgement of Saint-Loup’s work. However, to avoid tautonymy (i.e., when genus name and species epithet are identical), he also unconventionally changed the species epithet to *marioni* substituting *Pachydrilus enchytraeoides* with *Enchytraeoides marioni*. Furthermore, Roule provided an extensive description of the embryology and development of his species along with over a hundred illustrations (Roule 1889). The male anatomy of *E. marioni* is in many ways reminiscent of some species of *Lumbricillus*, such as *L. arenarius* (Michaelsen, 1889), in lacking the typical regularly lobed testis sacs of most *Lumbricillus* and having sperm funnels several times longer than wide. In fact, *L. arenarius* has been placed in *Enchytraeoides* by some authors (Ude 1929; Knöllner 1935; von Bülow 1957). It is not completely clear if *Pachydrilus enchytraeoides* and *Enchytraeoides marioni* are indeed the same species. Regardless, the descriptions of both species fit within *Lumbricillus*, and thus *Enchytraeoides* should be considered a junior synonym of *Lumbricillus* (Stephenson 1930). A more extensive account on the intricate taxonomical history of *Enchytraeoides* and other genera can be found in Rota et al. (2008).

Among the new species described by Claparède (1861) when he established the genus *Pachydrilus* was *P. semifuscus* Claparède, 1861. This species was noted to have the body colorless anteriorly and brown posteriorly (due to the strongly pigmented chloragogen tissue), conspicuous nephridia, solid unlobed testes and huge penial bulbs. The species was later transferred to *Marionia* (a name soon after replaced by *Marionina*) by Michaelsen (1889) and remained there until Nielsen and Christensen (1959) moved it to *Lumbricillus*. The brief original description was amended partly by Southern (1909) and more extensively by Stephenson (1911), who reported 4 to 5 pairs of pharyngeal glands, along with details of the chaetae, brain, copulatory glands and more. The species was also described from Iceland by Erséus (1976), who observed that the anteseptale of the nephridia might contain more than just a funnel (also a few coils). It has later been noted that this species (as well as others) lack the typical lobes of the testes and thus might not fit in *Lumbricillus* (Kossmagk-Stephan 1983).

The molecular phylogenetic study by Erséus et al. (2010) documented that *Lumbricillus* is a non-monophyletic group, and that a group of species (including *L. arenarius*) is sister to *Grania* Southern, 1913 rather than to the remaining *Lumbricillus*. However, the more recent analysis of the North European *Lumbricillus* by Klinth et al. (2017) showed ambiguity as to whether three *Lumbricillus* species (*L. arenarius*, *L. dubius* (Stephenson, 1911) and an unidentified species) comprise the sister group of the remaining *Lumbricillus* or the sister to *Grania*, where they together with *Grania* in turn would be the sister to *Lumbricillus* sensu stricto. The same study also found strong evidence for excluding what they refer to as “*L. semifuscus*” from *Lumbricillus* sensu stricto (Klinth et al. 2017). Lately, with increased taxon sampling that species (which we here describe as *C. semifuscoides* sp. n.) has been found to be more closely related to *Globulidrilus* Christensen & Dózsa-Farkas, 2012 and *Bryodrilus* Ude, 1892 (Martinsson et al. 2017).

The aim of this study is to increase the knowledge of the taxonomy of North European *Lumbricillus*, based on the most recent phylogenetic reconstruction and molecular delimitation of species (Klinth et al. 2017), and herein, by the addition of morphological studies. However, we will not use the morphological characters for any phylogenetic analysis. We will provide illustrated descriptions of all the included species and establish three new species and one new genus and re-describe *L. helgolandicus* (Michaelsen, 1927), in order to clear up parts of the taxonomy of this poorly studied group.

## Material and methods

Worms were collected in marine, brackish and limnic habitats, mainly in Norway and Sweden (Appendix 1), by decantation of suspended organic material from bottom substrates and killed and preserved in 80 % ethanol. After sorting under a dissecting microscope, the posterior end of each worm was cut away for DNA analyses (partly published by Klinth et al. 2017) while the anterior end was stained in paracarmine, dehydrated in xylene and mounted in Canada balsam on microscope slides; for a more detailed description of procedures see Erséus (1994). Using a compound microscope, specimens on slides were identified to species using the primary taxonomic literature, and largely adhering to the list of accepted species in Schmelz and Collado (2012), with the following exceptions: *L. aegialites* Stephenson, 1922 and *L. georgiensis* Tynen, 1969, both synonymized with *L. pagenstecheri* (Ratzel, 1868) by Coates & Ellis, 1981, *L. magdalenae* Nurminen, 1965 which we consider a synonym of *L. arenarius* (Michaelsen, 1889), and finally *L. cervisiae* Kossmagk-Stephan, 1983 and *L. christenseni* Tynen, 1966, which we consider separate species from *L. knoellneri* Nielsen & Christensen, 1959 with which they were previously synonymized. In the species descriptions we provided brief chresonymy lists of the references we found most relevant for each species. Morphological characters were drawn using a camera lucida and the images were treated by Gimp 2.8.10 software. All specimens studied are vouchers of DNA sequences (Appendix 1), including the COI-barcodes generated by Klinth et al. (2017) study, which were also used in this study to find matches between our specimens and records in the Barcoding of Life Database (**BOLD**), to better estimate the geographical ranges of the species. Where available, Barcode Index Numbers or BIN:s have been noted in each species description. These BIN:s refer to clusters of COI-barcodes on BOLD that are considered to comprise specimens of the same species. Finally, the only remaining syntype of *Pachydrilus helgolandicus* Michaelsen, 1927, herein designated as the lectotype, was borrowed from the Zoological Museum in Hamburg (**ZMH**). It has now been mounted and morphologically examined. Types and other voucher specimens are deposited in the Swedish Museum of Natural History (**SMNH**) and the Zoological Museum, University of Bergen (**ZMBN**).

## Results

### General notes

All descriptions are based on fixed worms mounted on slides. This has some disadvantages for discerning the shape of certain internal organs such as the nephridia, but is not an unusual method for marine worms, and it improves the description of other characters such as chaetae. Nevertheless, morphology can differ from descriptions in the literature based on living specimens. Welch (1914) noted that fixation in alcohol can reduce *Lumbricillus* body length from about 15–19 mm in living specimens, to about 9–14 mm in fixed specimens. Finogenova and Streltsov (1978) noted that the ratio between the length and width of the sperm funnels was similarly reduced in fixed specimens. They reported sperm funnels about 2–4 times longer than wide in living specimens and a ratio of about 1.2–1.5:1 after fixation. Furthermore, Finogenova and Streltsov (1978) as well as Southern (1909) reported the ratio length:width of the sperm funnels to vary in living specimens due to body contractions. Southern (1909) further questioned the importance of the midventral subneural glands (previously referred to as copulatory glands) for separating species, as these glands seem to vary in size and sometimes in segmental distribution between individuals of the same species. We also observed great variation in these features. Lastly, as the colour cannot be distinguished after the staining, remarks on the colour of the worms is based on the notes made by the collector, prior to preservation and mounting.

All specimens in this study are amputated of their posterior segments (used for DNA extraction). Therefore, comparisons of total length and segment number with original descriptions have not been possible. When available, the length of the fifteen first segments as well as the width at the clitellum of the worms has been used to compare the general body size of the species.

In fixed *Lumbricillus* worms the origin of the dorsal vessel can be difficult to establish since vessel expansions are more or less conspicuous according to the peristaltic movement of the blood at the time of fixation. Thus, due to the varying conditions when animals were killed and fixed, the dorsal vessel may appear to originate in different segments.

### Abbreviations in the figures

as=anteseptale, b=brain, cl=clitellum, dg=duct glands, e=egg, ed=ectal duct, eg=ectal gland, mu=musculature, nd=nephridial duct, oe=ooesophagus, ov=ovaries, pb=penial bulb, pg=pharyngeal glands, ps=postseptale, s=spermatheca, sa=spermathecal ampulla, sf=sperm funnel, sm=sperm mass, sp=spermathecal pore, t=testis, ts=testis sac, vd=vas deferens.

## Taxonomy

### Lumbricillus

Taxon classificationAnimaliaEnchytraeidaEnchytraeidae

Ørsted, 1844

#### Genus description/diagnosis.

Mainly red, pink, orange, yellow or white when alive, sometimes green or black. Living worms ranging from about 5 to 20 (35 in extremes) mm, fixed from 3 to 14 mm (35 mm in *L. maximus* (Michaelsen, 1888) even after fixation; Rota 2001). Prostomium hemispherical. Head pore at 0/1. Epidermis with transverse rows of gland cells. Chaetae usually sigmoid, sometimes straight, without nodulus, upper bundles varying from a dorsolateral to a midlateral position. Oesophageal appendages absent. Pharyngeal glands in three pairs, located in IV–VI, usually converging dorsally, sometimes connected dorsally, usually with ventral lobes, but secondary glands absent. Only nucleated coelomocytes present. Dorsal vessel originating intra- or in a segment posterior to clitellum. Nephridia with anteseptale made up of funnel only. Clitellum covering XII-XIII, sometimes also extending over parts of XI. Testes surrounded by peritoneal sacs; the latter usually made up of large lobes arranged in a regular bunch, in some smaller species forming a compact mass, only slightly and irregularly lobed. Penial bulbs round and compact, in a few species bilobed. Midventral subneural glands usually present in XIII–XV, sometimes further back. Spermathecae in V, sometimes extending further back, attached to and usually communicating with lumen of oesophagus; glands surrounding ectal part of ectal duct, sometimes also along ectal duct. Spermathecae either club-shaped with ampulla distinctly set off from duct or spindle-shaped without clear distinction between ampulla and duct. Spermathecal diverticula absent. Mainly living in the littoral zone of the sea but some species also found in limnic and/or terrestrial habitats.

#### Type species.

*Lumbricus lineatus* Müller, 1774.

#### Other species.

See Table 1 and notes below.

#### Remarks.

Based on the recent phylogenetic analysis of North European *Lumbricillus*, a number of monophyletic groups within the genus were recognized (Klinth et al. 2017). Several of these are distinguished by a combination of morphological characters that we refer to when discussing the taxonomy below. For convenience, we informally divide the species investigated into five groups based on their morphology: the *lineatus* group, the *pagenstecheri* group, the *buelowi* group and the *arenarius* group, all molecularly monophyletic, and the “*tuba*” group, which is molecularly paraphyletic (thence the quotation marks) (see Klinth et al. 2017) (Table 1, Fig. 1). The *lineatus*, *pagenstecheri* and “*tuba*” groups all have testis sacs with several large lobes in a bunch-like arrangement, characteristic for the majority of *Lumbricillus* species. The testis sacs of the *buelowi* and *arenarius* groups appear as a more or less compact irregular mass, which can still be lobed but not bunch-like. Interestingly, we noted that in the former three groups the upper and lower chaetal bundles are arranged almost symmetrically around the body (as dorsolateral and ventrolateral bundles), whereas in the *buelowi* and *arenarius* groups the upper bundles tend to be closer to the lateral lines (observed by all three authors). The number of chaetae varies within each group and is usually 3–6 in each bundle but can reach 10 or more, except in the *buelowi* and *arenarius* groups where there are rarely more than 2–3 chaetae per bundle. The length/width ratio of the sperm funnels varies within and among the species in each group, but the funnels are usually only a few times longer than wide in all groups except for the *lineatus* and *arenarius* groups, where they can be 5–10 times longer than wide. The penial bulbs are round and compact in all groups except the *arenarius* group where they can also be bilobed. Finally, the spermathecae are spindle-shaped without a clear distinction between the ectal duct and the ampulla in the *lineatus* group; have a clear distinction between ectal duct and ampulla in the *pagenstecheri*, “*tuba*” and *buelowi* groups, with gland cells also along the duct in the *pagenstecheri* group; or have a gradually widening ectal duct, making the ampulla more or less indistinct in the *arenarius* group (Table 1, Fig. 1). Using these combinations of characters we made a preliminary placement of the remaining *Lumbricillus* species, not studied in this paper, into any of these five groups (Table 1). Note that, as we have not been able to include these species in our molecular phylogeny, we cannot be certain that our species groups would remain monophyletic with the addition of these species.

**Table 1.** The informal division of *Lumbricillus* into five morphological groups based on the phylogenies by Klinth et al. (2017), together with comparisons of some morphological characters. Furthermore, the *Lumbricillus* species not included in the present study are listed with tentative placements to one of these groups. Note that it is likely that some of the species not studied could upon closer examination prove to be synonyms to the included species in this study. * *L. latithecatus* sp. n. was referred to as *L.* sp. E in Klinth et al. (2017). ** *L. scandicus* was referred to as L. cf. helgolandicus in Klinth et al. (2017).

	**Monophyletic (Based solely on Klinth** et al. **2017)**	**Testis sacs**	**Spermathecal shape**	**Spermathecal duct glands**	**Penial bulb**		**Sperm funnels**	**Chaetae per bundle**
*lineatus* group	Yes	Regularly lobed into bunch-shape	Spindle-shaped, indistinct ampulla	No	Round		1-5 times longer than wide	3-6 or more
*pagenstecheri* group	Yes	Regularly lobed into bunch-shape	Club-shaped, distinct ampulla	Yes	Round		About twice longer than wide	3-6 or more
“*tuba*” group	No	Regularly lobed into bunch-shape	Club-shaped, distinct ampulla	No	Round		About as long as wide	3-6
*buelowi* group	Yes	Irregularly lobed without bunch-shape	Club-shaped, distinct ampulla	No	Round		About as long as wide	2-3
*arenarius* group	Yes	Irregularly lobed without bunch-shape	Pouch-shaped, indistinct ampulla	No	Round or bilobed		3-10 times longer than wide	2-3
	**Species included in the present study**		**Species not included in the present study, placed on the basis of their descriptions**					
*lineatus* group	*L. fennicus* Nurminen, 1964 *L. kaloensis* Nielsen & Christensen, 1959 *L. latithecatus* sp. n. * *L. lineatus* (Müller, 1774) *L. pumilio* Stephenson, 1932a *L. rivalis* Levinsen, 1884 *L. rubidus* Finogenova & Streltsov, 1978 *L. rutilus* Welch, 1914 *L. verrucosus* (Claparède, 1861) *L.* sp F *L.* sp G		*L. aestuum* (Stephenson, 1932b) *L. alaricus* Shurova, 1974 *L. antarcticus* Stephenson, 1932b *L. americanus* (Ude, 1896) *L. benhami* Stephenson, 1932b *L. enteromorphae* von Bülow, 1957 *L. griseus* (Stephenson, 1932b) *L. healyae* Rodriguez & Rico, 2008 *L. incisus* Wang & Liang, 1997 *L. insularis* (Ude, 1896) *L. immoderatus* Finogenova, 1988 *L. macqueriensis* Benham, 1905 *L. maximus* (Michaelsen, 1888)*L. minimus* (Černosvitov, 1929)			*L. minutus* (Müller, 1776) sensu Michaelsen, 1911 *L. murmanicus* Finogenova & Streltsov, 1978 *L. parabolus* Shurova, 1978 *L. parvus* (Ude, 1896) *L. pseudominutus* Timm, 1988 *L. pygmaeus* (Michaelsen, 1935) *L. rupertensis* Coates, 1981 *L. sadovskyi* Marcus, 1965 *L. santaeclarae* Eisen, 1904 *L. scoticus* Elmhirst & Stephenson, 1926 *L. werthi* (Michaelsen, 1905)		
*pagenstecheri* group	*L. pagenstecheri* A-D (Ratzel, 1868) *L. viridis* Stephenson, 1911		*L. annulatus* Eisen, 1904 *L. belli* Tynen, 1969 *L. corallinae* Shurova, 1977 *L. curtus* Coates, 1981 *L. franciscanus* Eisen, 1904 *L. ignotus* Shurova, 1977 *L. kalatdlitus* Nurminen, 1970 *L. kamtschatkanus* (Michaelsen, 1929) *L. kurilensis* Shurova, 1974 *L. maritimus* (Ude, 1896) *L. merriami* Eisen, 1904*L. mirabilis* Tynen, 1969			*L. nipponicus* (Yamaguchi, 1937) *L. orientalis* Shurova, 1974 *L. pinquis* Shurova, 1977 *L. qualicumensis* Tynen, 1969 *L. reynoldsoni* Backlund, 1948 *L. rufulus* Shurova, 1974 *L. taisiae* Shurova, 1978 *L. tenuis* (Ude, 1896) *L. tsimpseanis* Coates, 1981 *L. sapitus* Shurova, 1979 *L. similis* Shurova, 1977		
	**Species included in the present study**		**Species not included in the present study, placed on the basis of their descriptions**					
“*tuba*” group	*L. scandicus* sp. n. ** *L. tuba* Stephenson, 1911 *L. helgolandicus* (Michaelsen, 1927)		*L. balticus* von Bülow, 1957 *L. charae* (Tynen, 1970) *L. imakus* Nurminen, 1970*L. lentus* Shurova, 1978			*L. macrothecatus* Erséus, 1976 *L. niger* Southern, 1909 *L. ochotensis* Shurova, 1979		
*buelowi* group	*L. buelowi* Nielsen & Christensen, 1959 *L. knoellneri* Nielsen & Christensen, 1959		*L. cervisiae* Kossmagk-Stephan, 1983 *L. eltoni* (Stephenson, 1924) syn. *L. knoellneri*?*L. mangeri* (Michaelsen, 1914)			*L. muscicolus* (Stephenson, 1924) syn. *L. knoellneri*? *L. nielseni* Nurminen, 1965		
*arenarius* group	*L. arenarius* (Michaelsen, 1889) *L. dubius* (Stephenson, 1911) *L.* sp. H		*L. christenseni* Tynen, 1966 *L. crymodes* (Stephenson, 1922) syn. *L. arenarius*? *L. eudioptus* (von Bülow, 1955) *L. westheidei* Kossmagk-Stephan, 1983					
Species with uncertain placement	*L. algensis* Erséus, 1977 (seminal vesicles irregularly lobed without bunch-shape but otherwise *lineatus*-like) *L. brunoi* Martinez-Ansemil, 1982 (seminal vesicles irregularly lobed without bunch-shape but otherwise *lineatus*-like) *L. colpites* (Stephenson, 1932b) (seminal vesicles irregularly lobed without bunch-shape, penial bulb with several lobe-shaped glands) *L. horridus* Finogenova, 1988 (intricate penial apparatus unlike that of other *Lumbricillus*) *L. intricatus* Finogenova, 1977 (spermathecae with ventral openings, nodulate chaetae, but otherwise *lineatus*-like)							

### Preliminary key to species groups (based on species included in study only)

**Table:** 

1	Chaetae 3–6 (or higher) per bundle, upper bundles located dorsolaterally; testis sacs with lobes in bunch-like arrangement	**2**
–	Chaetae 2–3 (occasionally higher) per bundle, upper bundles located midlaterally, just above the lateral line; testis sacs irregularly lobed, not bunch-like	**4**
2	Spermathecae with short, indistinct ducts, and spindle-shaped ampullae; sperm funnels about 1–5 times longer than wide	***lineatus* group**
–	Spermathecae club-shaped, with rather long ducts and clearly set-off ampullae; sperm funnels about 1–2 times longer than wide	**3**
3	Sperm funnels about as long as wide; no glands along each spermathecal duct inside compact gland around duct at spermathecal pore	”***tuba*” group**
–	Sperm funnels about 2 times longer than wide; numerous glands along each spermathecal duct (inside compact gland around duct at spermathecal pore)	***pagenstecheri* group**
4	Sperm funnels about as long as wide; spermathecae club-shaped, with rather long ducts and clearly set-off ampullae	***buelowi* group**
–	Sperm funnels about 3–10 times longer than wide; spermathecae club-shaped, but ducts gradually widening into ampullae	***arenarius* group**

### The *lineatus* group

*Characteristics*: Testis sacs regularly lobed in bunch-like arrangement. Spermathecae spindle-shaped with short duct which is difficult to distinguish from ampulla, and glands surrounding the ectal pore. Chaetae sigmoid and usually 3–6 or more per bundle; upper bundles located dorsolaterally. Penial bulbs round. Sperm funnel from as long as wide to about 5 times longer than wide.

### Lumbricillus lineatus

Taxon classificationAnimaliaEnchytraeidaEnchytraeidae

(Müller, 1774)

Figs 2 , 3A

**Figure 2.**
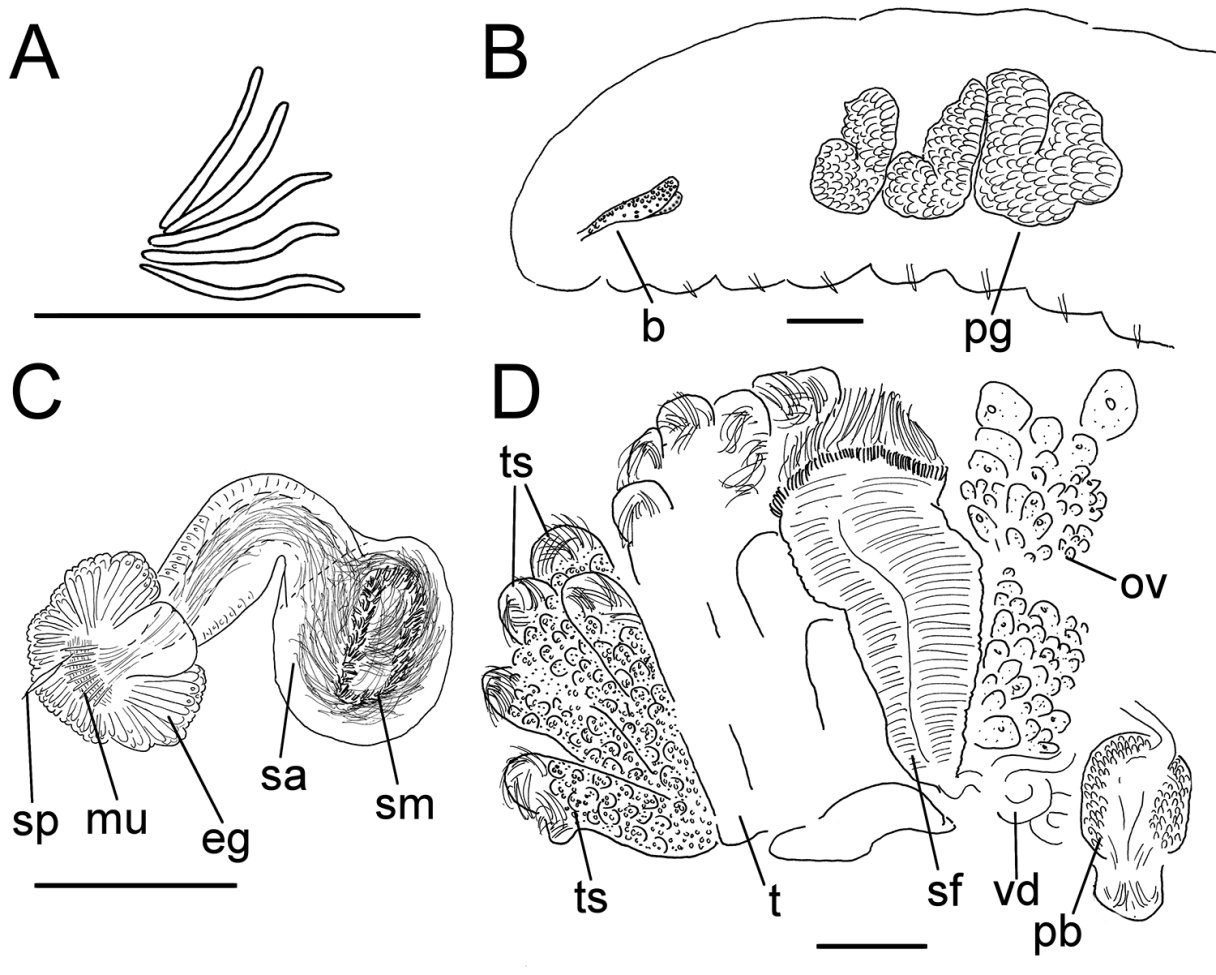
*Lumbricillus lineatus*. **A** Chaetal bundle **B** Anterior body **C** Spermatheca (neotype) **D** Other genitalia. Abbreviations under general notes. Scale bars: 100 µm.

Lumbricus lineatus Müller, 1774: p. 29.
Lumbricillus lineatus ; Erséus et al. 1999; Erséus et al. 2010; Klinth et al. 2017.
Pachydrilus claparedeanus Ditlevsen, 1904: pp. 431–435, figs 28 a–b.
Lumbricillus agilis Moore, 1905: pp. 395–397, pl. XXXIII, figs 23–28.
Lumbricillus lineatus partim; Michaelsen 1900: p. 80; Welch 1917: pp. 123–130; Nielsen and Christensen 1959: pp. 100–102, figs 109–112.
“Lumbricillus lineatus L2”; BOLD (unpublished records)
Non Pachydrilus lineatus; sensu Backlund 1947: pp. 3–5, figs 1–2 (see Lumbricillus latithecatus sp. n. below).

#### Type material (neotype).

*Lumbricillus lineatus* was described long before reference to types had become common practice and there is no remaining original material (“Typus amissus” in Nomenclatura Oligochaetologica). We designate SMNH Type-8931 [former SMNH 152751] (CE1894) as a neotype of this species. It is a whole-mounted voucher of a sexually mature and DNA-barcoded worm (COI barcode is KU894040 in NCBI/GenBank; Klinth et al. 2017) from the Baltic Sea, Öland, Mörbylånga, Skärlöv fishing harbor, on beach with mixed shelly sand, pebbles and organic material, 56.4241 N, 16.5815 E, collected 10 June 2006 by Lisa Matamoros, Sweden. Our decision to designate this neotype is further discussed in Remarks below.

#### Other material examined.

SMNH 152742 (CE1640), one mature specimen from Sweden; SMNH 152744 (CE1694), one mature avesiculate triploid specimen from Spain; ZMBN 107872 (CE12043), ZMBN 107875 (CE21688), ZMBN 107879 (CE22604) & ZMBN 107880 (CE22605), four mature specimens from Norway; ZMBN 107878 (CE21986), one mature avesiculate triploid specimen from Norway. For information on collection localities and GenBank accession numbers for COI barcodes see Appendix 1.

#### Description.

Orange, red or pink worms. Length (fixed worms) more than 2.2–5.5 mm (amputated specimens), first 15 segments 2.1–2.8 mm long, width at clitellum 0.45–0.75 mm. More than 14–38 segments. Chaetae sigmoid (Fig. 2A). Upper bundles dorsolateral, with 3–6 chaetae anterior to clitellum, 3–7 chaetae in postclitellar segments. Ventral bundles with 4–8 chaetae anterior to clitellum, 3–7(8) chaetae posteriorly. Each worm’s longest measured chaetae 50–75 µm long, about 3–5 µm wide. Clitellum extending over XII–1/2XIII/XIII. Head pore not observed.

Coelomocytes, in some specimens numerous, 10–20 µm long, round, oval or spindle-shaped, granulated with distinct nucleus. Paired pharyngeal glands present in IV, V and VI; each pair converging dorsally (Fig. 2B). Dorsal vessel originating in XII–XIV. Nephridia observed in VIII–X and XIII–XX, about 85–110 µm long. Anteseptale small, consisting of funnel only. Postseptale oval, tapering into posteroventral efferent duct. Brain with posterior incision.

Male genitalia paired (Fig. 2D). Testes originating in XI, extending forwards into X, sometimes IX, with testis sacs forming regular club-shaped lobes, except in two “avesiculate” specimens (SMNH 152744 and ZMBN 107878) which have atrophic testes. Sperm funnels in XI, 215–420 µm long, 125–185 µm wide, making them about 1.5–2.5 times longer than wide, funnels tapering towards vasa deferentia. Most of vasa irregularly coiled in XII, 15–20 µm wide. Penial bulbs round, 65–140 µm in diameter. Ovaries in XII. One to six mature eggs present at a time.

**Figure 3.**
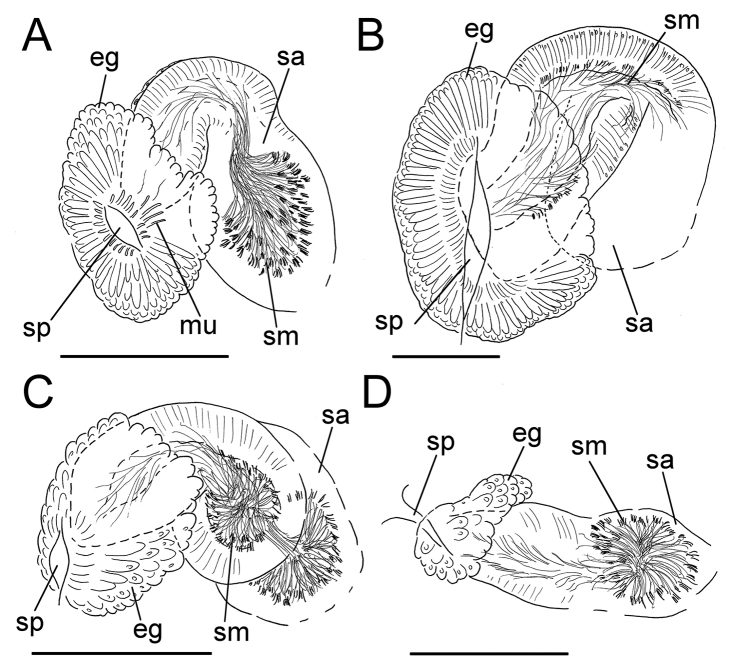
**A** *Lumbricillus lineatus*, spermatheca **B** *Lumbricillus latithecatus* sp. n. (holotype), spermathecae **C** *Lumbricillus verrucosus*, spermatheca **D** *Lumbricillus verrucosus*, spermatheca seen from below. Scale bars: 100 µm.

Spermathecae (Figs 2C, 3A) in V, spindle-shaped, without distinct ampulla. Ectal duct very short, widening into ampulla. Ampulla with constriction midway dividing it into sections, ectal part narrow, ental part wider, sometimes circular, connecting with oesophagus. Sperm in lumen of ectal part of ampulla, heads of spermatozoa embedded in wall of ental part of ampulla, forming aggregates. Spermathecae 220–275 µm long, 60–125 µm wide at widest part of ampulla. Gland cells surrounding ectal duct at pore, forming compact mass, glandular body 80–150 µm in diameter at its widest part. Two midventral subneural glands in XIII–XIV, 60–110 µm and 60–95 µm long, respectively.

#### Details of neotype.

Length 3.3 mm (amputated specimen), first 15 segments 2.4 mm long, width at clitellum 0.45 mm. 20 segments. Dorsal bundles with 4–6 chaetae anterior to clitellum, 3–4 chaetae in postclitellar segments. Ventral bundles with 6–8 chaetae anterior to clitellum, 3–5 chaetae posteriorly. Longest chaetae 50 µm, about 3 µm wide. Clitellum extending over XII–XIII.

Coelomocytes 15 µm long. Dorsal vessel originating in XIII. Nephridia observed in VIII–X and XIII–XX, about 110 µm long.

Testes originating in XI, extending forwards into X. Sperm funnels folded, length and width unclear. Vasa deferentia 15 µm wide. Penial bulbs 115 µm in diameter. No mature eggs observed.

Spermathecae (Fig. 2C) 270 µm long, 120 µm wide at widest part of ampulla. Glandular body at ectal pore 115 µm in diameter at its widest part. No midventral subneural glands observed.

#### Geographical distribution including BOLD data.

Genetically identified from the Netherlands, Norway, Spain and Sweden; also recognized from Canada (BIN-numbers BOLD:AAF9630 & BOLD:ACV7068). This species has historically been widely reported from Europe and North America, and even from the southern hemisphere (Stephenson 1932b).

#### Remarks.

*Lumbricillus lineatus*, possibly the first ever described enchytraeid, has an interesting history and was for a long time poorly defined as a species. It was given the name *Lumbricus lineatus* by Müller (1774) who described it as abundant on the shores of the Baltic Sea. Müller classified the worm under “Setosa”, and gave a brief description of its circulatory system and short protruding chaetae. In the same work, he refers to a worm that he previously described (Müller 1771), which he found on sandy shores of the Baltic and particularly among the rotting seaweed by the ramparts of Copenhagen, where he did most of his work. However, he classified that worm in *Gordius*, referred to it as a Faden Wurm (nematode) said to lack any segments or ring, and he failed to mention anything about chaetae. It is therefore difficult to say if Müller simply missed those characters or if the worm from 1771 was something different. Nevertheless, as Müller worked with live material, it is most plausible that the type locality for the first described *Lumbricillus lineatus* is around Copenhagen. Unfortunately, we do not have any specimens from Copenhagen, but we have found that our molecularly defined *L. lineatus* is common throughout the Baltic Sea. For the sake of finally defining the true *L. lineatus* and connecting it to a molecular profile, we decided to designate one of our specimens from Öland in the Baltic Sea as a neotype.

The specimens of *L. lineatus* in this study were smaller than the ones in the re-description by Nielsen and Christensen (1959) and the sperm funnels were shorter in relation to their length. However, the shape of the spermathecae, number of chaetae and segments conformed well with these authors’ description. Furthermore, both the vesiculate and avesiculate forms of the species were observed, as has previously been noted to occur in *L. lineatus* (Nielsen and Christensen 1959, Christensen 1961), further supporting the designation of a neotype from our material. Christensen and O’Connor (1958) were the first to describe diploid vesiculate and triploid avesiculate forms of *L. lineatus* and their intriguing life histories (see also Christensen 1960). While the diploid form has normal gametogenesis and is amphimictic, the triploid form is dependent on copulation with the diploid form, which acts as sperm donor, to produce offspring. Furthermore, the sperm from the diploid form does merely activate the egg of the triploid individual, without fertilizing it. Instead, oogenesis in the triploid cytotype follows a peculiar pattern of chromosome divisions and mergings that results ultimately in the restoration of triploid nuclei. The relationship between the two forms has been described as an obligatory co-existence (Christensen 1960). To further complicate things, tetra- and pentaploids have also been observed within *L. lineatus*. The tetra- and pentaploids have testis sacs that are smaller than those of diploids but larger than those of triploids. Furthermore, the sperm funnels are smaller in tetra- pentaploids than in di- and triploids. Apparently, the testes sacs of tetra- and pentaploids sometimes produce sperm which can activate the eggs of all polyploid forms (Christensen et al. 1978). Unfortunately, we did not determine the ploidy level of our sampled specimens, but we did not observe any genetic distinction between the vesiculate and avesiculate forms, neither in COI nor in ITS sequences (Klinth at al. 2017).

*Lumbricillus lineatus* is morphologically most similar to *L. verrucosus* and *L. latithecatus* sp. n. (compare spermathecae in Fig. 3 and see Remarks for these species), but also superficially similar to the other members of the group we have chosen to call the *linaetus* group. Genetically it is closely related to *L. rutilus* Welch, 1914 and *L. latithecatus* sp. n. (Klinth et al. 2017; where *L. latithecatus* is called *L.* sp. E) (Fig. 1).

### Lumbricillus rutilus

Taxon classificationAnimaliaEnchytraeidaEnchytraeidae

Welch, 1914

Fig. 4

Lumbricillus rutilus Welch, 1914: 143–151, pl. VIII, fig. 13, pl. IX, figs 14–24; Klinth et al. 2017.
“Lumbricillus rivalis”; BOLD (published records; Vivien et al. 2015)

#### Type material.

USNM 25507, 26318, 30863–4 (Nomenclatura Oligochaetologica). Type locality: Chicago Sewage Testing Station, United States (Welch 1914). Not studied.

#### Material examined.

SMNH 152801 (CE1887), SMNH 152802 (CE1903), SMNH 152804 (CE2510), SMNH 152809 (CE2937), SMNH 152811 (CE2939), SMNH 152813 (CE3060), SMNH 152814 (CE3061) & SMNH 152819 (CE9267), eight mature specimens from Sweden; SMNH 152815 (CE3502) & SMNH 152816 (CE3506), two mature specimens from the United Kingdom. For information on specimen collection locality and GenBank accession numbers see Appendix 1.

#### Description.

**Figure 4.**
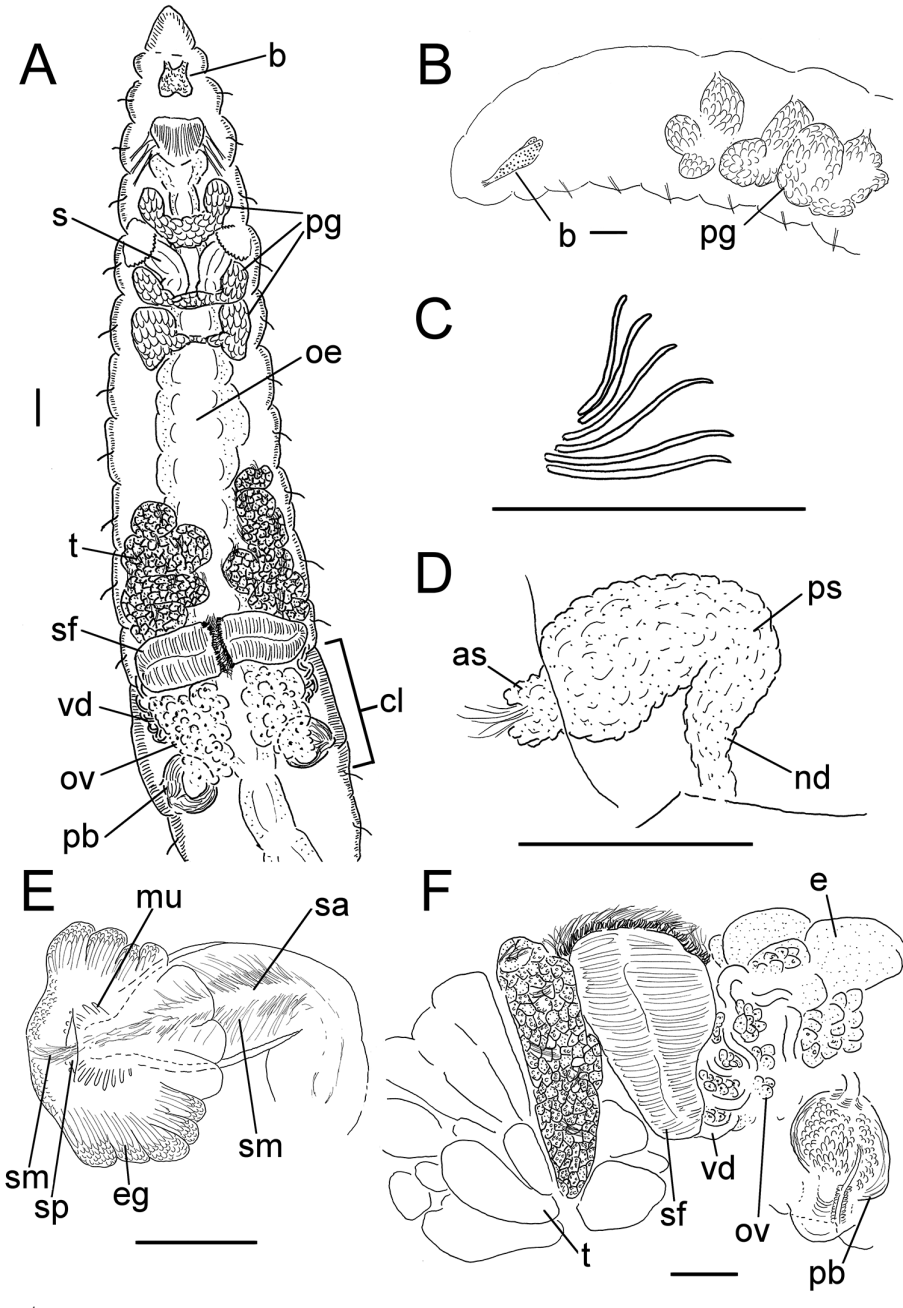
*Lumbricillus rutilus*. **A** Anterior body, seen from above **B** Anterior body **C** Chaetal bundle **D** Nephridium **E** Spermatheca **F** Other genitalia. Abbreviations under general notes. Scale bars: 100 µm.

Orange-reddish worms. Length (fixed worms) more than 2.7–7.2 mm (amputated specimens), first 15 segments 2.4–4.8 mm long, width at clitellum 0.39–0.65 mm. More than 14–30 segments. Chaetae (Fig. 4C) slightly sigmoid. Dorsal bundles with 3–6, rarely 2 or 7, chaetae anterior to clitellum, 2–5 chaetae in postclitellar segments. Ventral bundles with 3–9, usually 4–6, chaetae anterior to clitellum, 2–6 chaetae posteriorly. Each worm’s longest measured chaetae 70–105 µm long, about 3–5 µm wide. Clitellum generally extending over XII–1/2XIII, sometimes including all of XIII. Head pore at 0/1. Epidermis with transverse rows of gland cells.

Coelomocytes in some specimens numerous, 10–25 µm long, round, oval or spindle-shaped, granulated. Paired pharyngeal glands present in IV, V and VI; each pair converging dorsally (Fig. 4B). Third pair larger, occupying most of VI, sometimes extending into VII. Dorsal vessel originating in XIV. Nephridia (Fig. 4D) observed in XV–XXI, about 120–170 µm long. Anteseptale small, consisting of funnel only. Postseptale oval, tapering posteriorly into efferent duct. Brain (Fig. 1B) slightly widening posteriorly, with posterior incision.

Male genitalia paired (Fig. 4F). Testes originating in XI, extending forwards into X, sometimes IX, with testis sacs forming regular club-shaped lobes. Sperm funnels in XI, 295–395 µm long, 145–225 µm wide, making them about 1.5–2.5 times longer than wide, funnels tapering towards vasa deferentia. Most of vasa irregularly coiled in XII, 20–30 µm wide. Penial bulbs round, 110–175 µm in diameter. Ovaries in XII. Two to six mature eggs present at a time.

Spermathecae (Fig. 4D) in V, spindle-shaped, without distinct ampulla. Ectal duct short, muscular, widening into ampulla. Ampulla, after widest part, making sharp bend before entally connecting with oesophagus. Sperm evenly embedded in tissue of ectal duct and ampulla. Spermathecae 215–335 µm long, 70–115 µm wide at widest part of ampulla. Crown of gland cells surrounding ectal pore, lobed, whole glandular body 110–225 µm in diameter at its widest part. Up to four midventral subneural glands in XIII–XVI, 80–325 µm, 55–200 µm, 60–100 µm and 50 µm long, respectively; glands in XIII, XV or XVI not observed in all specimens.

#### Geographical distribution including BOLD data.

Originally described from USA, now genetically identified from Norway, Sweden and United Kingdom; also recognized from Canada and Switzerland (BIN-number: BOLD:ACV8942).

#### Remarks.

Our newly sampled material matches Welch’s (1914) description of *Lumbricillus rutilus* well in all characters, except for the sperm funnel shape, where our specimens had funnels less elongate in relation to their width. The material in this study was collected in Sweden and the United Kingdom, whereas this species was originally described from a sewage treatment plant in Chicago, USA. Interestingly, some of our sampled specimens also come from two such plants, in Sweden and the UK, respectively. The species was additionally collected in littoral and freshwater environments in Sweden, and it is likely to be an opportunist that thrives in nutrient-rich habitats. Specimens found in the treatment plants showed increased body size and reduced number of chaetae per bundle, compared to the specimens sampled in the sea, possibly a side effect of living in such rich environments.

*Lumbricillus rutilus* is genetically most closely related to *L. lineatus* and *L. latithecatus* sp. n. (Fig. 1). However, morphologically it is more similar to *L. rivalis*, particularly in the shape of the spermathecae. *Lumbricillus rutilus* has on average fewer chaetae per bundle compared with *L. rivalis* and sperm funnels that are shorter in relation to their width.

### Lumbricillus latithecatus sp. n.

Taxon classificationAnimaliaEnchytraeidaEnchytraeidae

http://zoobank.org/575B08C8-3F02-4D35-B9FF-814F52DD3573

Figs 3B , 5

“Lumbricillus lineatus L1”; BOLD (unpublished records)
Lumbricillus sp. E; Klinth et al. 2017.
? Pachydrilus lineatus; sensu Backlund 1947: pp. 3–5, figs 1–2.

#### Holotype.

ZMBN 107940 (CE12041), a whole-mounted voucher of a sexually mature and DNA-barcoded worm (COI barcode is KU894054 in NCBI/GenBank; Klinth et al. 2017).

#### Type locality.

Norway, Rogaland, Sola, Ölbörhamna, intertidal in decomposing algae, 58.8697N, 5.5654E, collected 15 June 2012 by C. Erséus. Norway.

#### Paratype.

ZMBN 107941 (CE12042), one whole-mounted sexually mature specimen from the type locality.

#### Other material examined.

SMNH 152830 (CE1976) & SMNH 152831 (CE1979), two mature specimens from Sweden. For information on specimen collection localities and GenBank accession numbers see Appendix 1.

#### Etymology.

Named from the Latin *latus* meaning wide and *theca* for spermatheca.

#### Diagnosis.

This species can be distinguished from other *Lumbricillus* species by the shape of the spermathecae, which do not gradually widen from the ectal pore but instead originates from a very wide pore followed by an ectal duct and ampulla of even width throughout. This makes the duct and ampulla of the spermathecae virtually indistinguishable. There is at least a superficial similarity to the spermathecae of *Lumbricillus lineatus* and *L. verrucosus* with a midway constriction or bend and sperm aggregated in the ental part of the ampulla (Fig. 3). However, the spermathecae of *L. lineatus* and *L. verrucosus* have ectal pores that are much smaller than the diameter of their ampullae, giving the impression of a rapid widening of the spermathecae after the ectal pore, even though the duct and ampulla are difficult to distinguish also in these species.

#### Description of all material.

**Figure 5.**
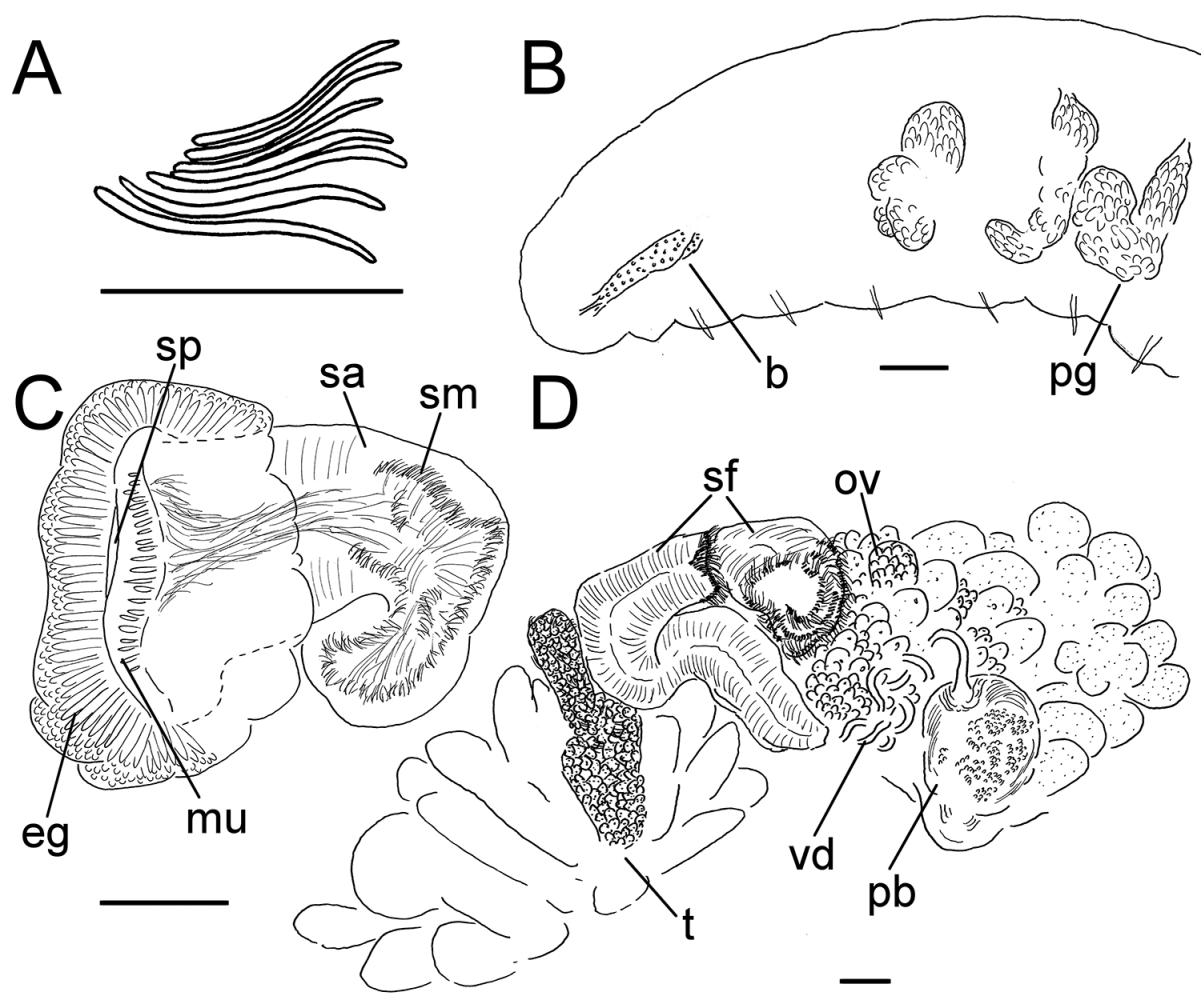
*Lumbricillus latithecatus* sp. n. **A** Chaetal bundle **B** Anterior body **C** Spermatheca **D** Other genitalia. Abbreviations under general notes. Scale bars: 100 µm.

Length (fixed worms) more than 2.5–7.8 mm (amputated specimens), first 15 segments 2.5–4.7 mm long, width at clitellum 0.42–0.85 mm. More than 15–27 segments. Chaetae slightly sigmoid (Fig. 5A). Dorsal bundles with (3)4–8(9) chaetae anterior to clitellum, 3–7 chaetae in postclitellar segments. Ventral bundles with 5–10 chaetae anterior to clitellum, 4–9 chaetae posteriorly. Each worm’s longest measured chaetae 60–75 µm long, about 4–5 µm wide. Clitellum extending over XII–XIII. Head pore not observed. Epidermis with transverse rows of gland cells.

Coelomocytes numerous, 10–25 µm long, round, oval or spindle-shaped, granulated. Paired pharyngeal glands present in IV, V and VI; each pair converging dorsally (Fig. 5B). Dorsal vessel originating in XIII. Nephridia examined in IX and XIV–XXV, about 75–155 µm long. Anteseptale small, consisting of funnel only. Postseptale oval, tapering posteriorly into efferent duct. Brain with posterior incision.

Male genitalia paired (Fig. 5D). Testes originating in XI, extending forwards into IX, with testis sacs forming regular club-shaped lobes. Sperm funnels in XI, sometimes extending into X or XII, 360–1300 µm long, 155–235 µm wide, making them about 2–6 times longer than wide, funnels tapering towards vasa deferentia. Most of vasa irregularly coiled in XII, 15–30 µm wide. Penial bulbs round, 155–285 µm in diameter, possibly with a small ventral lobe set off from the rest of the bulb. Ovaries in XII. One to four mature eggs present at a time.

Spermathecae (Figs 3B, 5C) in V, pouch-shaped, without distinct ampulla. Ectal duct seemingly indistinguishable from ampulla as the wide pore is followed by a lumen that remains about the same width or possibly widening slightly. Ectal pore surrounded by mass of gland cells forming compact body 140–325 µm in diameter at its widest part. Ampulla, with possible constriction midway dividing it into two sections; ental connection with oesophagus. Sperm filling middle of ectal duct, heads of spermatozoa embedded and forming aggregates mainly in ental part of ampulla. Each spermatheca altogether 220–410 µm long, 65–160 µm wide at widest part of ampulla. Up to three midventral subneural glands in XIII–XV, 80–200 µm, 100–250 µm and 115 µm long, respectively; glands in XIV and XV not observed in all specimens.

#### Details of holotype.

The largest specimen of the lot. Length 7.8 mm (amputated specimen), first 15 segments 4.7 mm long, width at clitellum 0.85 mm. 27 segments. Dorsal bundles with 5–8 chaetae anterior to clitellum, 4–7 chaetae in postclitellar segments. Ventral bundles with 5–10 chaetae anterior to clitellum, 5–9 chaetae posteriorly. Longest measured chaetae 75 µm long, about 5 µm wide. Clitellum extending over XII–1/2XIII.

Coelomocytes 10–25 µm long. Dorsal vessel originating in XIII. Nephridia observed in IX and XXIII–XXV, about 125–135 µm long.

Testis sacs extending forwards into IX. Sperm funnels in XI, 1100 µm long, 210 µm wide, making them about 5 times longer than wide. Vasa deferentia 30 µm wide. Penial bulbs 285 µm in diameter. No mature eggs present.

Spermathecae (Fig. 3B) 335 µm long, 135 µm wide at widest part of ampulla. Glandular body at ectal pores 275 µm in diameter at its widest part. Midventral subneural glands in XIII and XIV, 200 µm and 150 µm long, respectively.

#### Geographical distribution including BOLD data.

Genetically identified from Norway and Sweden; also recognized from Denmark (BIN-number BOLD :AAU0294).

#### Remarks.

The measured lengths of the sperm funnels are probably underestimated due to the difficulty of tracing them through the worms and due to folding. The two Swedish specimens were somewhat smaller than the Norwegian ones, and their funnels folded and only measurable for about 360 µm, but the length:width ratio was close to 4–6:1, as noted for the Norwegian specimens.

The description of *Pachydrilus lineatus* by Backlund (1947), from a drainpipe in Southern Sweden, in some ways reminds of our new species. Backlund was uncertain if his species belonged to *P. lineatus* because of the very wide spermathecal duct (which seemed as wide at the pore as in its medial part), the lack of a distinct ampulla and the possession of a large gland around the ectal pore. Furthermore, he described the penial bulbs as bilobed with a larger dorsal and a smaller ventral lobe. The description of the spermathecae sounds like the one of those of *L. latithecatus*, and after having examined the penial bulbs in the whole-mounted specimens of the latter species, it seems as if there could be a small lobe hidden behind the large spherical lobe (when viewed laterally). However, we would need transverse sections to truly compare this character to that which Backlund described. Lastly, Backlund reported small pharyngeal glands without dorsal development, which does not match with what we have observed for our species. Therefore, we are not certain as to the identity of Backlund’s *P. lineatus*.

*Lumbricillus latithecatus* is genetically most closely related to *L. lineatus* and *L. rutilus* (Fig. 1).

### Lumbricillus verrucosus

Taxon classificationAnimaliaEnchytraeidaEnchytraeidae

(Claparède, 1861)

Figs 3C–D , 6

Pachydrilus verrucosus Claparède, 1861: pp. 82–85, pl. I, figs 1–6;
Lumbricillus verrucosus ; Michaelsen 1900: p. 80; Klinth et al. 2017.
Pachydrilus lineatus forma verrucosus ; Černosvitov 1937: p. 292.
Lumbricillus lineatus partim; Nielsen and Christensen 1959: pp. 100–102, figs 109–112.

#### Type material.

Typus amissus (Nomenclatura Oligochaetologica). Type locality: Sound of Sleat, Isle of Skye, Hebrides, United Kingdom (Claparède, 1861). We did not designate a neotype as we do not have material from the type locality.

#### Material examined.

SMNH 152826 (CE968), one mature specimen from Sweden, ZMBN 107919 (CE21479), ZMBN 107920 (CE21486), ZMBN 107921 (CE21490), ZMBN 107922 (CE21494), ZMBN 107924 (CE21811), ZMBN 107925 (CE21816) & ZMBN 107926 (CE21821), seven mature specimens from Norway. For information on specimen collection localities and GenBank accession numbers see Appendix 1.

#### Description.

**Figure 6.**
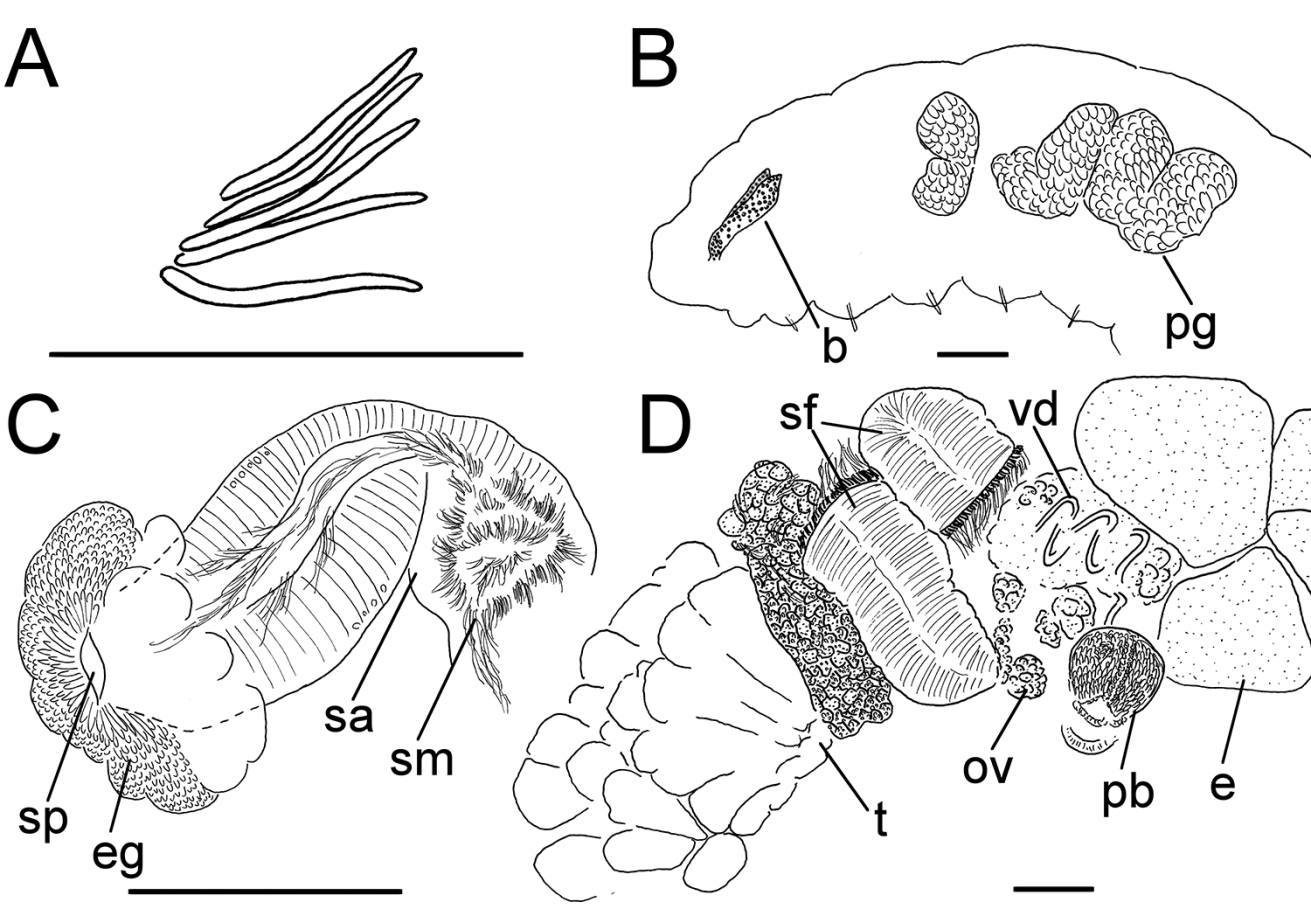
*Lumbricillus verrucosus*. **A** Chaetal bundle **B** Anterior body **C** Spermatheca **D** Other genitalia. Abbreviations under general notes. Scale bars: 100 µm.

White to yellow worms. Length (fixed worms) more than 2.3–5.7 mm (amputated specimens), first 15 segments 2.3–3.4 mm long, width at clitellum 0.42–0.60 mm. More than 18–33 segments. Chaetae slightly sigmoid (Fig. 6A). Dorsal bundles with (2)3–5(6) chaetae anterior to clitellum, 2–4 chaetae in postclitellar segments. Ventral bundles with (2)3–6(7) chaetae anterior to clitellum, (2)3–4(5) chaetae posteriorly. Each worm’s longest measured chaetae 45–60 µm long, about 2.5 µm wide. Clitellum extending over XII–XIII. Head pore at 0/1. Epidermis with transverse rows of gland cells.

Coelomocytes in some specimens numerous, 10–25 µm long, round, oval or spindle-shaped, granulated. Paired pharyngeal glands present in IV, V and VI; each pair converging dorsally (Fig. 6B). Dorsal vessel originating in XIII. Nephridia observed in XIV–XXV, 75–120 µm long. Anteseptale small, consisting of funnel only. Postseptale oval, tapering posteriorly into efferent duct. Brain twice as long as wide, with posterior incision.

Male genitalia paired (Fig. 6D). Testes originating in XI, extending forwards into X, sometimes IX, with testis sacs forming regular club-shaped lobes. Sperm funnels in XI, 230–370 µm long, 125–175 µm wide, making them about 1.5–2.5 times longer than wide, funnels tapering towards vasa deferentia. Most of vasa irregularly coiled in XII, 15–20 µm wide. Penial bulbs round, 105–140 µm in diameter. Ovaries in XII. One to five mature eggs present at a time.

Spermathecae (Figs 3C–D, 6C) in V, spindle-shaped, without distinct ampulla. Ectal duct short, widening into ampulla. Ampulla with constriction midway to two thirds of the length, dividing it into two sections, the inner one of which connecting with oesophagus. Sperm filling lumen of ectal duct, heads of spermatozoa embedded in inner part of ampulla, sometimes also in outer part, forming aggregates. Spermathecae 180–300 µm long, 65–110 µm wide at widest part of ampulla. Gland cells surrounding ectal pore, forming compact mass, whole glandular body 95–180 µm in diameter at its widest part. One or two midventral subneural glands in XIII–XIV, 90–125 µm and 95–125 µm long, respectively; gland in XIV not observed in all specimens.

#### Geographical distribution.

Originally described from the United Kingdom, now genetically identified from Norway and Sweden. The full extent of this species’ distribution is difficult to ascertain since it was previously synonymized with *L. lineatus*, a species distributed worldwide. BIN-number BOLD:ACV7714.

#### Remarks.

*Lumbricillus verrucosus* was originally described by Claparède (1861) and later synonymized with *L. lineatus* by Nielsen and Christensen (1959), probably following Černosvitov (1937) who had downgraded the former to a form of the latter. Specimens from the two species examined in this study are indeed very similar when considering the shape of the spermathecae (Fig. 3) and most body measurements, but they differ in body colour, chaetal number and the proportions of the sperm funnels: indeed *Lumbricillus verrucosus* was described as being pale yellow, having 3–5 chaetae and sperm funnels about three times longer than wide (Claparède 1861), against *L. lineatus* being orange-red, having more chaetae and sperm funnels about five times longer than wide. Furthermore, the spermathecae of *L. verrucosus* seem to have an ampulla that is slightly longer and wider in the part ectal to the constriction, but the importance of this character remains to be proved. Even though the two species may be very difficult to separate morphologically, except perhaps by body colour, they are supported as separate species molecularly and avesiculate specimens have been found by us only in *L. lineatus*, not in *L. verrucosus*.

Interestingly, *Lumbricillus verrucosus* is genetically most closely related to *L. rivalis* (Levinsen, 1883) and not to *L. lineatus* (Fig. 1). However, these three species are well supported as closely related to each other.

### Lumbricillus rivalis

Taxon classificationAnimaliaEnchytraeidaEnchytraeidae

(Levinsen, 1883)

Fig. 7

**Figure 7.**
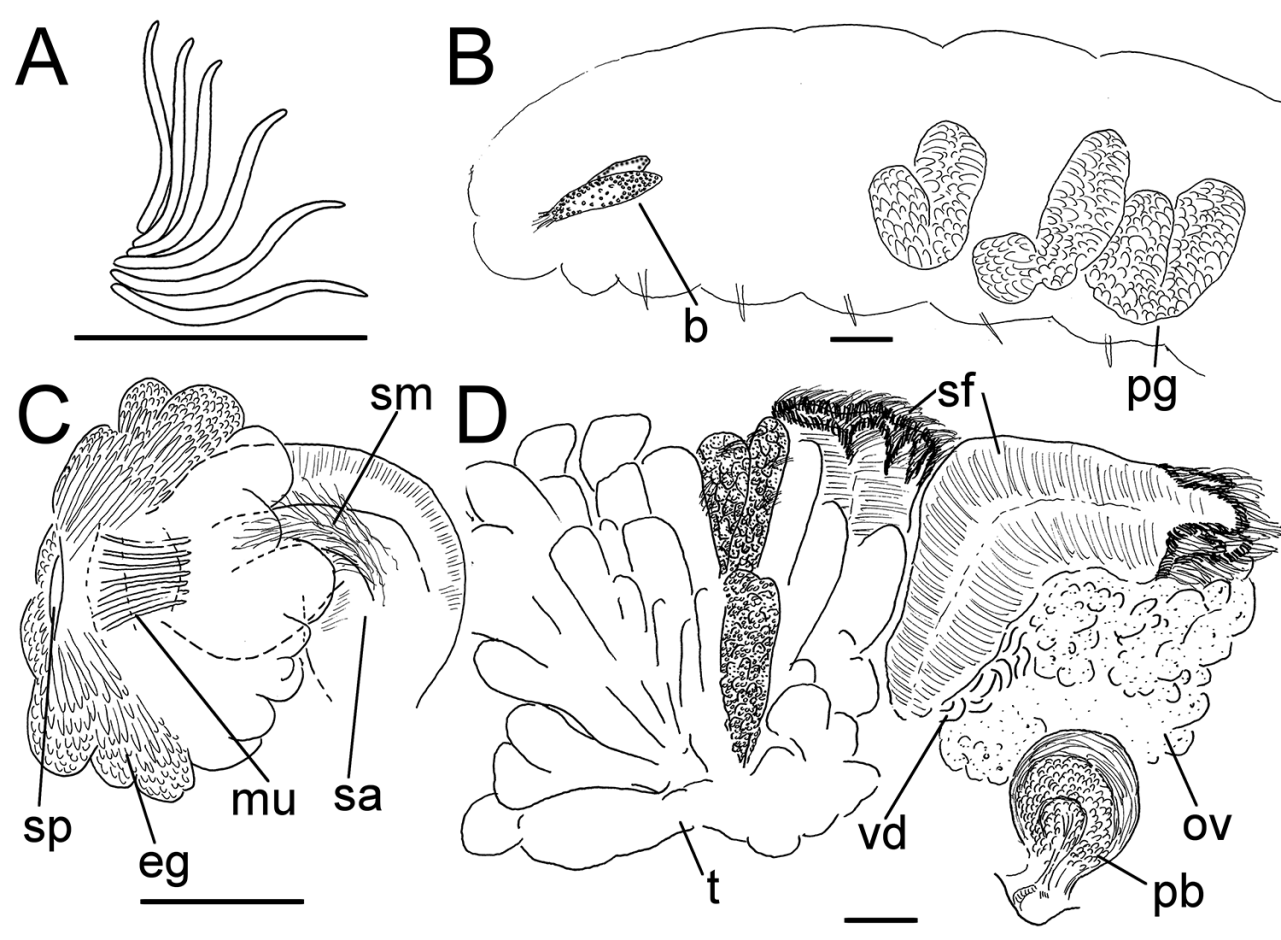
*Lumbricillus rivalis*. **A** Chaetal bundle **B** Anterior body **C** Spermatheca **D** Other genitalia. Abbreviations under general notes. Scale bars: 100 µm.

**Figure 8.**
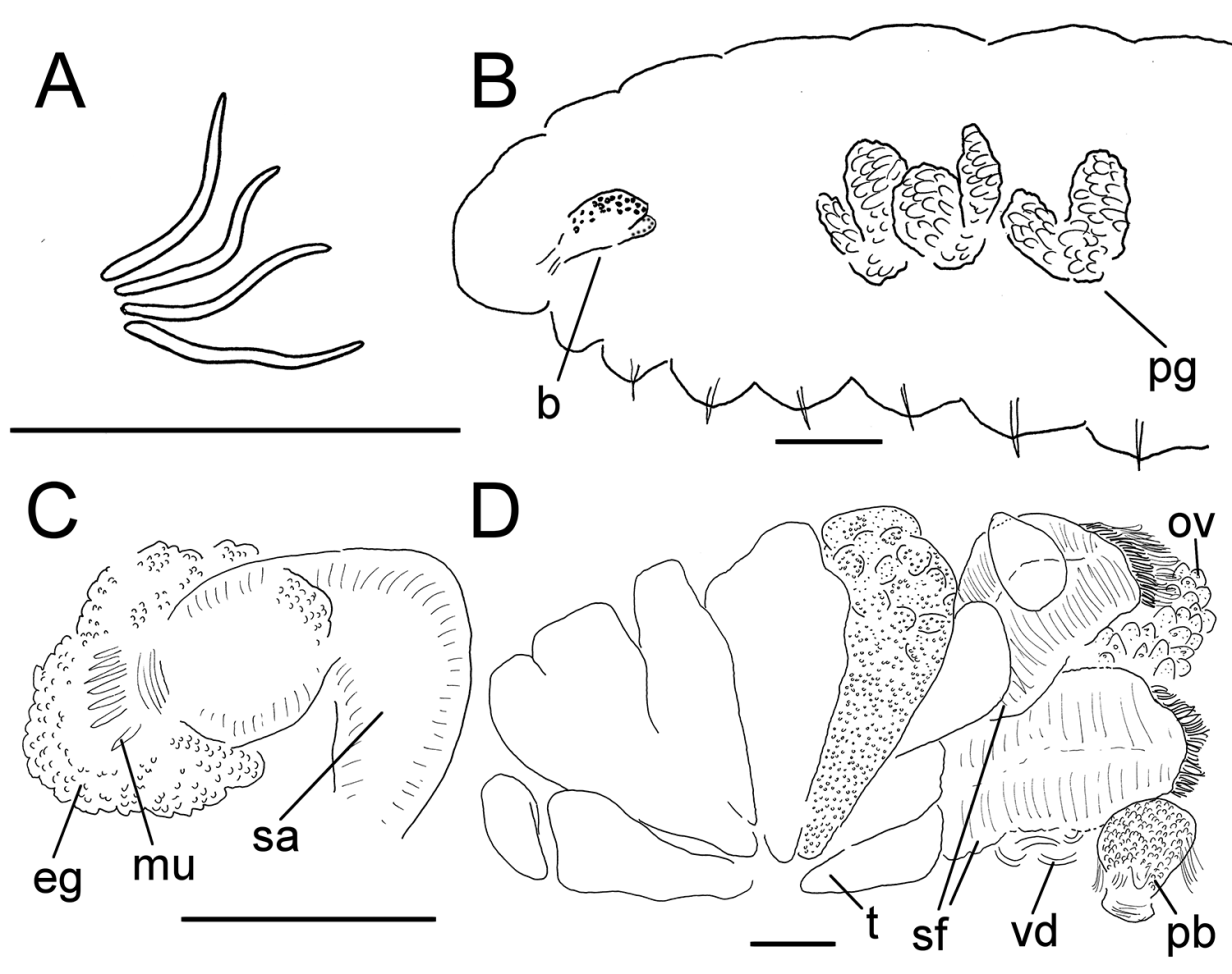
*Lumbricillus* sp. G. **A** Chaetal bundle **B** Anterior body **C** Spermatheca **D** Other genitalia. Abbreviations under general notes. Scale bars: 100 µm.

**Figure 9.**
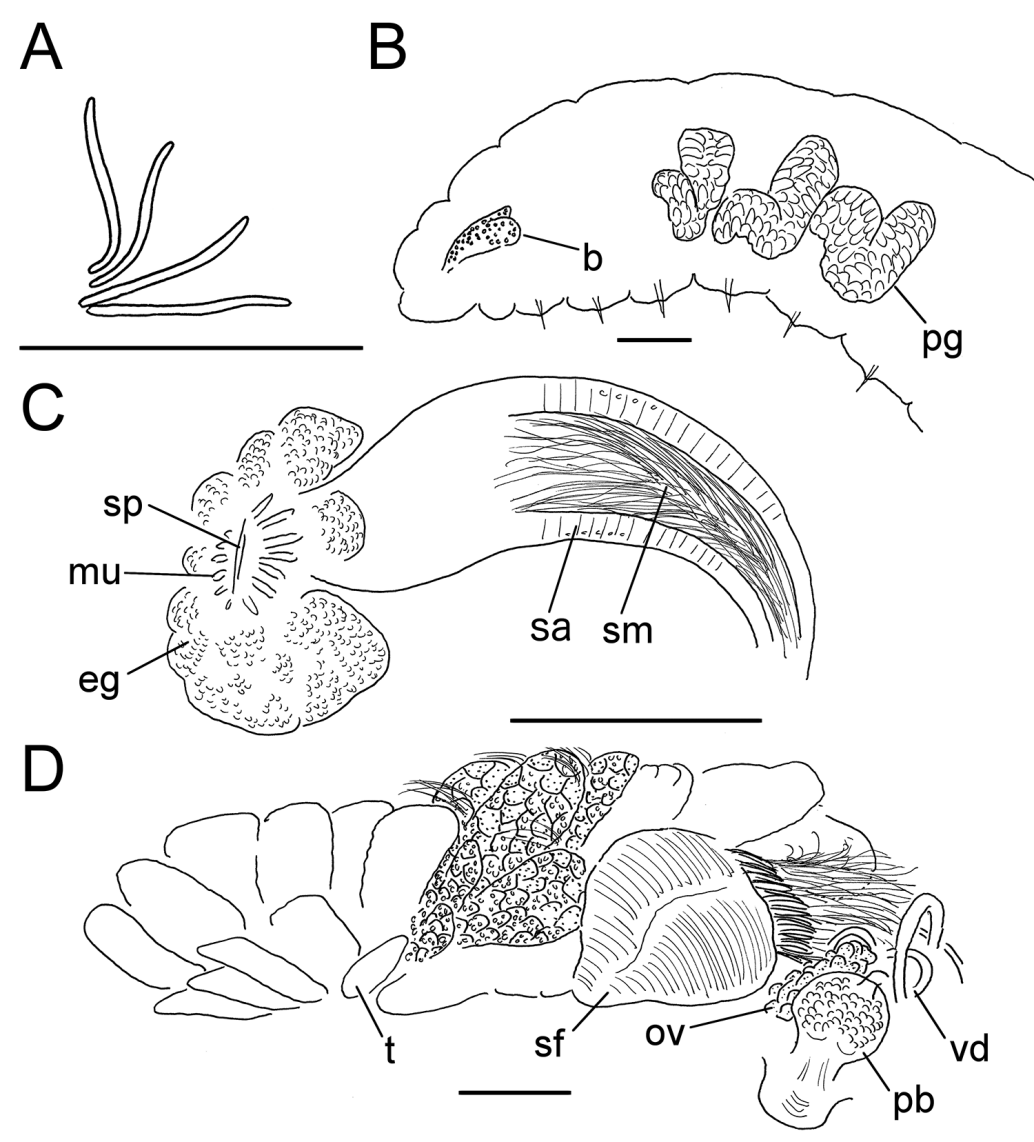
*Lumbricillus kaloensis*. **A** Chaetal bundle **B** Anterior body **C** Spermatheca **D** Other genitalia. Abbreviations under general notes. Scale bars: 100 µm.

**Figure 10.**
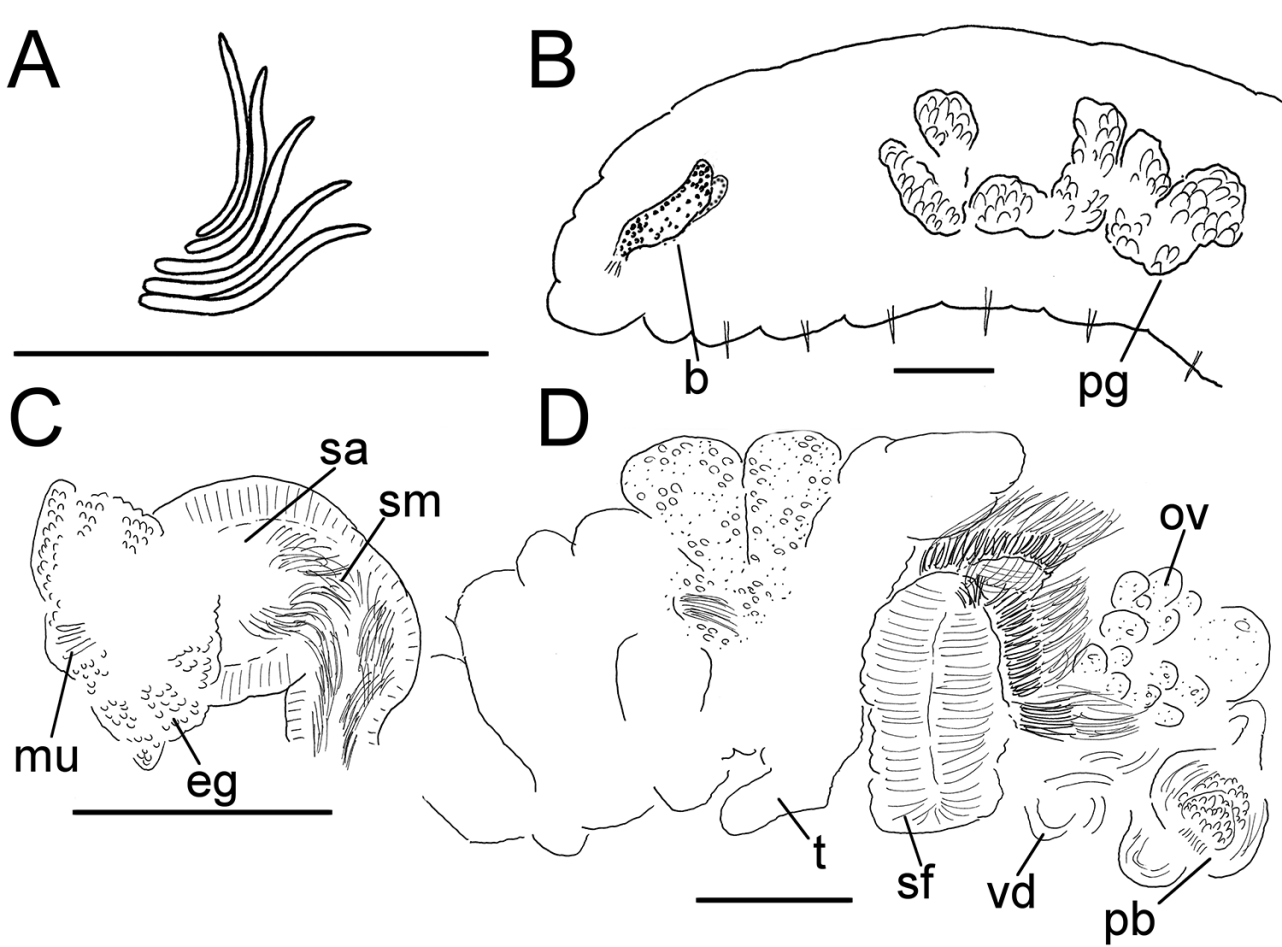
*Lumbricillus* sp. F. **A** Chaetal bundle **B** Anterior body **C** Spermatheca **D** Other genitalia. Abbreviations under general notes. Scale bars: 100 µm.

Pachydrilus rivalis Levinsen, 1883: p. 231; Ditlevsen 1904: pp. 430–431.
Lumbricillus rivalis ; Nielsen and Christensen 1959: pp. 97–98, figs 107–108; Erséus et al. 1999; Erséus et al. 2010; Klinth et al. 2017.
Pachydrilus subterraneus Vejdovsky, 1889: pp. 1–3.
Pachydrilus germanicus Michaelsen, 1886: pp. 43–44.
Lumbricillus evansi Southern, 1909: pp. 151–152, pl. X, figs 10a–f.
Non Lumbricillus enteromorphae von Bülow, 1957: pp. 82–84, pl. XXVI, figs 6–10, pl. XXVII, fig. 1, pl. XXX, fig. 16.

#### Type material.

Typus amissus (Nomenclatura Oligochaetologica). Type locality: Langelinie, Denmark (Levinsen, 1883). We did not designate a neotype as we do not have material from the type locality.

#### Material examined.

SMNH 152782 (CE1873), SMNH 152783 (CE1874) & SMNH 152785 (CE2503), three mature specimens from Sweden, ZMBN 107897 (CE22596), ZMBN 107898 (CE22600) & ZMBN 107899 (CE22602), three mature specimens from Norway. For information on specimen collection localities and GenBank accession numbers see Appendix 1.

#### Description.

Orange-red worms. Length (fixed worms) more than 3.4–7.6 mm (amputated specimens), first 15 segments 2.6–3.8 mm long, width at clitellum 0.60–0.85 mm. More than 17–44 segments. Chaetae slightly sigmoid (Fig. 7A). Dorsal bundles with 4–8(9) chaetae anterior to clitellum, (3)4–7(8) chaetae in postclitellar segments. Ventral bundles with (4)5–10(11) chaetae anterior to clitellum, (4)5–8(9) chaetae posteriorly. Each worm’s longest measured chaetae 85–105 µm long, about 5 µm wide. Clitellum extending over XII–XIII. Head pore at 0/1. Epidermis with transverse rows of gland cells.

Coelomocytes numerous, 15–20 µm long, spindle-shaped, oval, granulated. Paired pharyngeal glands present in IV, V and VI; each pair converging dorsally (Fig. 7B). Dorsal vessel originating in XIV. Nephridia observed in VII–X and XV–XXIII, about 145–170 µm long. Anteseptale small, consisting of funnel only. Postseptale oval, tapering posteriorly into efferent duct. Brain with posterior incision, about as long as wide.

Male genitalia paired (Fig. 7D). Testes originating in XI, extending forwards into X, sometimes IX, with testis sacs forming regular club-shaped lobes. Sperm funnels in XI, 325–685 µm long, 110–295 µm wide, making them about 1.5–4.5 times longer than wide, funnels tapering towards vasa deferentia. Most of vasa irregularly coiled in XII, 25 µm wide. Penial bulbs round, 105–190 µm in diameter. Ovaries in XII. Two mature eggs present in one specimen.

Spermathecae (Fig. 7C) in V, spindle-shaped, without distinct ampulla. Ectal duct short, gradually widening into ampulla. Ampulla with constriction two thirds of the length from pore, dividing it into sections, entally connecting with oesophagus. Sperm evenly embedded in ampulla. Spermathecae 250–360 µm long, 85–140 µm wide at widest part of ampulla. Gland cells surrounding ectal pore forming lobed collar, 140–240 µm in diameter at its widest part. Up to three midventral subneural glands in XIII–XV, 90–130 µm, 80–145 µm and 80–90 µm long, respectively; glands in XV not observed in all specimens.

#### Geographical distribution.

Genetically identified from Greenland, Norway and Sweden; also recognized from Canada (BOLD:AAF9076). This species was originally described from Denmark and is considered well distributed throughout Europe, and North America.

#### Remarks.

*Pachydrilus rivalis* was originally defined by Levinsen (1884) as a species with up to 9 chaetae per bundle, spermatheca formed by a large, red, pear-shaped container (Danish “beholder”), ending ectally with a glandular rosette, but bearing no glands on duct. The nephridial efferent duct originated at the posterior end of postseptale; the postseptals had red spots; the body color was red. Michaelsen (1889, 1900) placed the species in synonymy with *L. lineatus* along with his own *Pachydrilus germanicus* Michaelsen, 1886, but Ditlevsen (1904) reinvestigated Levinsen’s type locality (Langelinie, a pier in the port of Copenhagen) and found that only one species conformed to Levinsen’s short primary diagnosis, a species that was different from *L. lineatus*. He thus expanded the description of *rivalis* by adding the following traits: body length 15–20 mm, copulatory glands in XIII–XV, dorsal vessel from XIV, chaetal number 6–9 dorsally, 8–11 ventrally. Nevertheless, Welch (1917) and Černosvitov (1937) adhered to Michaelsen’s opinion and the species had to await Nielsen and Christensen (1959) to be revalidated.

Our new specimens fit well with the more detailed re-descriptions, particularly in the number of chaetae and the shape of the spermathecae. The only differences concern the body size, where our worms are much smaller than those reported before, and the length/width ratio of the sperm funnels, which Nielsen and Christensen described as up to 10 times longer than wide, compared to our observed 4.5 times. These differences could be explained by our examination of fixed instead of live material.

*Lumbricillus rivalis* is genetically most closely related to *L. verrucosus* (Fig. 1), but it is morphologically more alike *L. rutilus* (see remarks for that species) and *L. enteromorphae*, although it can be distinguished from the latter by lacking an atrium-like part where the vas deferens meets the penial bulb (see remarks for *L. rubidus*).

### Lumbricillus

Taxon classificationAnimaliaEnchytraeidaEnchytraeidae

sp. G

Fig. 8

Lumbricillus sp. G; Klinth et al. 2017.

#### Material examined.

Specimen SMNH 152834 (CE2246) & SMNH 152835 (CE2661), one mature and one immature specimen from the United Kingdom, and ZMBN 107942 (CE23373), one immature specimen from Norway. For information on specimen collection localities and GenBank accession numbers see Appendix 1.

#### Description.

Colour of worms unknown. Length (fixed worms) more than 3.3–4.1 mm (amputated specimens), first 15 segments 2–2.8 mm long, width at clitellum 0.42–0.49 mm. More than 17–33 segments. Chaetae slightly sigmoid (Fig. 8A). Dorsal bundles with 2 –5 chaetae anterior to clitellum, 3–6 chaetae in postclitellar segments. Ventral bundles with 3–6 chaetae anterior to clitellum, 3–4 chaetae posteriorly. Each worm’s longest measured chaetae 55–60 µm long, about 5 µm wide. Clitellum extending over XII–1/2XIII. Head pore at 0/1. Epidermis with transverse rows of gland cells.

Coelomocytes numerous, 15–30 µm long, round, oval or spindle-shaped, granulated with distinct nucleus. Paired pharyngeal glands present in IV, V and VI (Fig. 8B). Dorsal vessel originating in XIII–XV. Nephridia not observed. Brain elongate, further details unknown.

Male genitalia paired (Fig. 8D). Testes originating in XI, extending forwards into IX, with testis sacs forming regular club-shaped lobes. Sperm funnels in XI, extending backwards into XII, 335–430 µm long, 180 µm wide, making them about 1.9–2.4 times longer than wide, funnels tapering towards vasa deferentia. Most of vasa irregularly coiled in XII, 15 µm wide. Penial bulbs pear-shaped, 110 µm in diameter. Ovaries in XII. One mature egg present in the single mature individual.

Spermathecae (Fig. 8C) in V, spindle-shaped, without distinct ampulla. Ectal duct short, widening into ampulla. Ampulla with constriction midway, dividing it into two sections, the inner of which connecting with oesophagus. No sperm observed. Spermathecae 210 µm long, 80 µm wide at widest part of ampulla. Gland cells surrounding ectal pore, forming compact mass, 105 µm in diameter at its widest part. Two midventral subneural glands in XIII–XIV, 70 µm and 90 µm long, respectively.

#### Geographical distribution.

Only known from Norway and the United Kingdom.

#### Remarks.

Unfortunately only one mature specimen was available for this study making it difficult to assign it to a known species. On the other hand, the description of a new species on such limited material would be premature. *Lumbricillus* sp. G is genetically placed within the *lineatus* group (Fig. 1).

### Lumbricillus kaloensis

Taxon classificationAnimaliaEnchytraeidaEnchytraeidae

Nielsen & Christensen, 1959

Fig. 9

Lumbricillus kaloensis Nielsen & Christensen, 1959: p. 100, figs 113–114; Erséus et al. 2010; Klinth et al. 2017.

#### Type material.

Typus amissus (Nomenclatura Oligochaetologica). Type locality: Kalø Vig, Denmark. We did not designate a neotype as we do not have material from the type locality.

#### Material examined.

SMNH 152733 (CE978), one mature specimen from Sweden & ZMBN 107842 (CE5412), one immature specimen from Norway. For information on specimen collection localities and GenBank accession numbers see Appendix 1.

#### Description.

Orange or whitish worms. Length (fixed worms) more than 2.8–3.6 mm (amputated specimens), first 15 segments 2.5–3.1 mm long, width at clitellum 0.32–0.37 mm. More than 18 segments. Chaetae slightly sigmoid (Fig. 9A). Dorsal bundles with 3–5 chaetae anterior to clitellum, 2–3 chaetae in postclitellar segments. Ventral bundles with 3–6 chaetae anterior to clitellum, 2–5 chaetae posteriorly. Each worm’s longest measured chaetae 50–60 µm long, about 3 µm wide. Clitellum extending over XII–1/2XIII. Head pore at 0/1. Epidermis with transverse rows of gland cells.

Coelomocytes numerous, 15 µm long, round, oval, granulated. Paired pharyngeal glands present in IV, V and VI; each pair converging dorsally (Fig. 9B). Dorsal vessel originating in XIII. Nephridia observed in IX–X and possibly XIV–XV, about 110 µm long. Anteseptale small, consisting of funnel only. Postseptale oval, tapering posteriorly into efferent duct. Brain with posterior incision.

Male genitalia paired (Fig. 9D). Testes originating in XI, extending forwards into IX, in one specimen also extending backwards into XII, with testis sacs forming regular club-shaped lobes. Sperm funnels in XI, 185 µm long, 155 µm wide, making them slightly longer than wide, funnels tapering towards vasa deferentia. Most of vasa irregularly coiled in XII, 15 µm wide. Penial bulbs round, 80 µm in diameter. Ovaries in XII. No mature eggs observed.

Spermathecae (Fig. 9C) in V, spindle-shaped, without distinct ampulla, gradually widening, entally connecting with oesophagus. Sperm evenly embedded in ampulla. Spermathecae 290 µm long, 65 µm wide at widest part of ampulla. Gland cells surrounding ectal pore divided into at least three somewhat separated lobes, one lobe significantly larger than the others, whole glandular body 120 µm in diameter at its widest part. Two midventral subneural glands in XIII–XIV, 95 µm and 75 µm long, respectively.

#### Geographical distribution including BOLD data.

Originally described from Denmark, now genetically identified from Norway and Sweden. BIN-number: BOLD:AAU0152.

#### Remarks.

The specimens examined in this study match the original description by Nielsen and Christensen (1959) in the majority of the characters. Our worms were smaller than theirs, with slightly fewer chaetae (particularly in postclitellar bundles) and sperm funnels shorter in relation to their width, the latter of which could be explained by the difference between live and mounted material. We also observed two subneural glands which were not originally reported by Nielsen and Christensen. The spermathecae, despite being slightly damaged in our mature specimen, exhibit the typical large asymmetrical ectal gland subdivided into flap-like lobes, one of which is clearly larger than the others. Asymmetrical ectal glands have also been observed in *L. enteromorphae* von Bülow, 1957, and to some extent in *L. rubidus* Finogenova & Streltsov, 1978. The former can be distinguished from *L. kaloensis* by having sperm funnels 8 times longer than wide, and an atrium-like part where the vasa deferentia meet the penial bulbs. *Lumbricillus rubidus*, while having ectal glands on the spermathecae that may appear asymmetrical, does not have the glands subdivided into such clearly asymmetrical flaps as in *L. kaloensis*. Furthermore, *L. rubidus* has a more distinct musculature covering the ectal duct of the spermathecae, sometimes appearing as a muscular bulb; *L. kaloensis* also has a muscular coating of the duct, but it is thinner.

*Lumbricillus kaloensis* is genetically placed within the *lineatus* group. Its possible sister-group relationship with *Lumbricillus* sp. F (suggested by the tree in Fig. 1) is not statistically supported by the DNA data.

### Lumbricillus

Taxon classificationAnimaliaEnchytraeidaEnchytraeidae

sp. F

Fig. 10

Lumbricillus sp. F; Klinth et al. 2017.

#### Material examined.

SMNH 152832 (CE2659), one mature specimen from the United Kingdom. For information on specimen collection locality and GenBank accession number see Appendix 1.

#### Description.

Colour of worms unknown. Length (fixed worm) more than 3.1 mm (amputated specimen), first 15 segments 2.2 mm long, width at clitellum 0.35 mm. More than 21 segments. Chaetae slightly sigmoid (Fig. 10A). Dorsal bundles with 3–5 chaetae anterior to clitellum, 3–4 chaetae in postclitellar segments. Ventral bundles with 3–5 chaetae anterior to clitellum, 2–4 chaetae posteriorly. The worm’s longest measured chaetae 45 µm long, about 3 µm wide. Clitellum extending over XII–1/2XIII. Head pore at 0/1. Epidermis with transverse rows of gland cells.

Coelomocytes numerous, 15 µm long, round, oval or spindle-shaped, granulated with distinct nucleus. Paired pharyngeal glands present in IV, V and VI (Fig. 10B). Dorsal vessel originating in XIV. Nephridia possibly observed in X, about 95 µm, shape uncertain. Brain elongate, with posterior incision.

Male genitalia paired (Fig. 10D). Testes originating in XI, extending forwards into X, with testis sacs forming regular club-shaped lobes. Sperm funnels in XI, 175 µm long, 90 µm wide, making them about twice as long as wide. Vasa deferentia not observed. Penial bulbs round, 85 µm in diameter. Ovaries in XII. About two or three mature eggs present.

Spermathecae (Fig. 10C) in V, spindle-shaped, without distinct ampulla. Ectal duct short, widening into ampulla. Ampulla with constriction midway, dividing it into two sections, the inner of which connecting with oesophagus. Sperm concentrated in inner part of ampulla, embedded in wall of ampulla. Spermathecae 210 µm long, 80 µm wide at widest part of ampulla. Gland cells surrounding ectal pore, forming compact mass, 110 µm in diameter at its widest part. Possibly one subneural gland in XIV, 50 µm long.

**Geographical distribution.** Only known from the United Kingdom and Norway (unpublished results).

**Remarks.** Unfortunately only one mature specimen was available for this study, making it difficult to assign it to a known species. On the other hand, the description of a new species on such limited material is not recommended. *Lumbricillus* sp. F is genetically placed within the *lineatus* group; it is possibly phylogenetically close to *L. kaloensis*, but this is not strongly supported (Fig. 1).

### Lumbricillus pumilio

Taxon classificationAnimaliaEnchytraeidaEnchytraeidae

Stephenson, 1932

Fig. 11

Lumbricillus pumilio Stephenson, 1932a: pp. 902–904, figs 1–3; Nielsen and Christensen 1959: p. 96; Tynen and Nurminen 1969: pp. 151–153; Klinth et al. 2017.
Lumbricillus pumillio (sic); Erséus 1976: p. 9.

#### Type material.

Typus amissus (Nomenclatura Oligochaetologica). Type locality: Wembury Bay, Plymouth, United Kingdom (Stephenson 1932a). We did not designate a neotype as we do not have material from the type locality.

#### Material examined.

SMNH 152775 (CE3346), SMNH 152776 (CE3347), SMNH 152777 (CE3427), SMNH 152778 (CE3428), SMNH 152779 (CE3430), SMNH 152780 (CE3436) & SMNH 152781 (CE3437), seven mature specimens from the United Kingdom. For information on specimen collection localities and GenBank accession numbers see Appendix 1.

#### Description.

**Figure 11.**
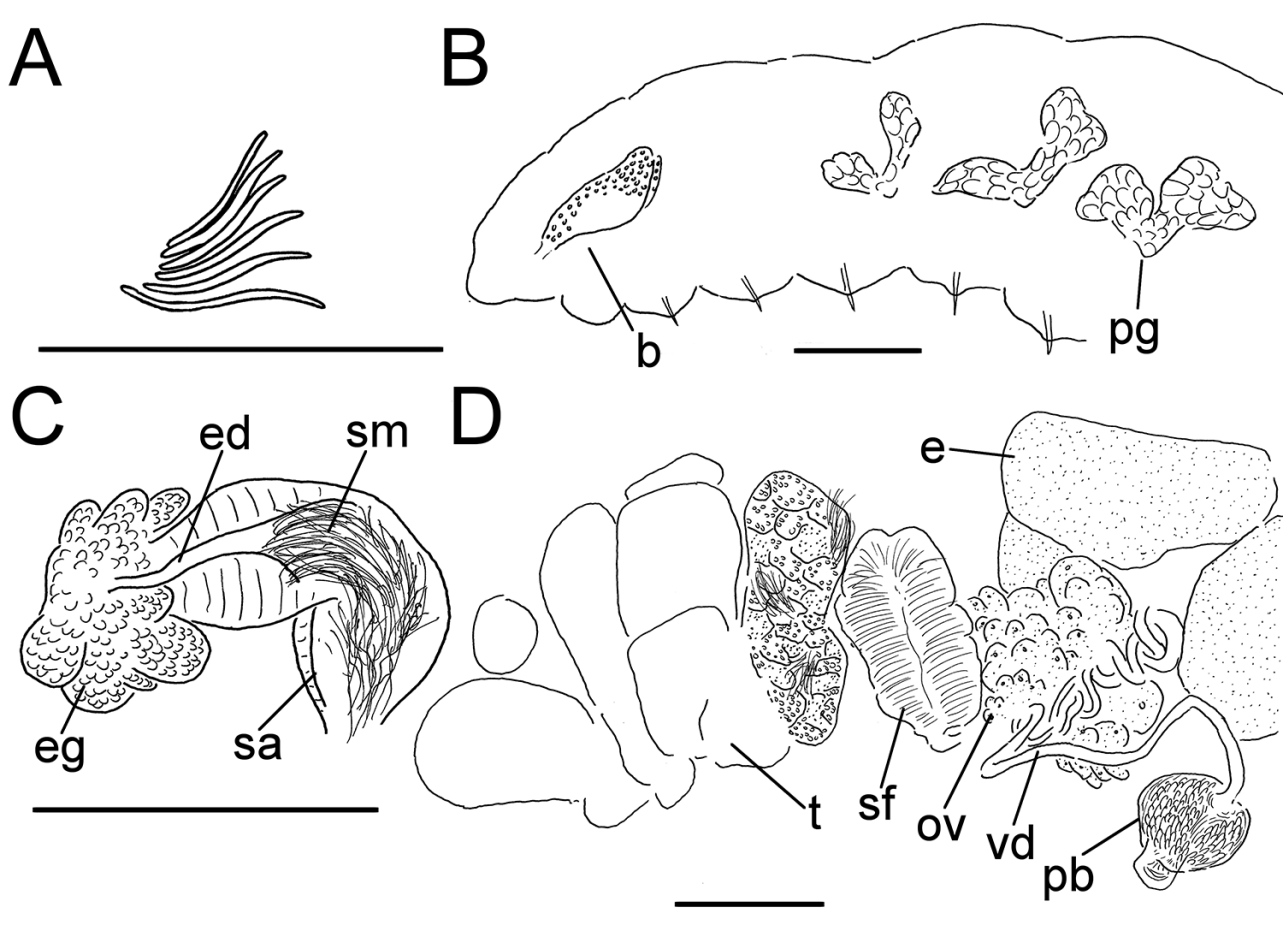
*Lumbricillus pumilio*. **A** Chaetal bundle **B** Anterior body **C** Spermatheca **D** Other genitalia. Abbreviations under general notes. Scale bars: 100 µm.

Short and stout worms. Colour unknown. Length (fixed worms) more than 1.7–3.2 mm (amputated specimens), first 15 segments 1.3–2.3 mm long, width at clitellum 0.24–0.38 mm. More than 15–23 segments. Chaetae slightly sigmoid (Fig. 11A). Dorsal bundles with 3–6 chaetae anterior to clitellum, 3–5 chaetae in postclitellar segments. Ventral bundles with 3–6, rarely 2 or 7, chaetae anterior to clitellum, 3–6 chaetae, rarely 2, posteriorly. Each worm’s longest measured chaetae 36–48 µm long and about 2.5 µm wide. Clitellum extending over XII–1/2XIII. Head pore not observed. Epidermis with transverse rows of gland cells.

Coelomocytes difficult to identify as such in this species, but small round, oval or spindle-shaped granulated cells about 5–7 µm long were noted in the coelomic cavity. Paired pharyngeal glands present in IV, V and VI; each pair converging dorsally (Fig. 11B). Dorsal vessel originating in XII or XIII. Nephridia observed in IX and XV–XVIII, about 45–50 µm long. Anteseptale small, consisting of funnel only. Postseptale oval, tapering posteriorly into efferent duct. Brain longer than wide, with a marked posterior incision creating two horn-like structures.

Male genitalia paired (Fig. 11D). Testes originating in XI, extending forwards into X, with testis sacs forming regular club-shaped lobes. Sperm funnels in XI, 115–190 µm long, 65–140 µm wide making them about 1.5–2 times longer than wide, funnels tapering towards vasa deferentia. Most of vasa deferentia irregularly coiled in XII together with ovaries, vasa 5–10 µm wide. Penial bulbs round, 55–80 µm in diameter. One or two mature eggs present at a time.

Spermathecae (Fig. 11C) in V, spindle-shaped. Ectal duct short, ampulla indistinct, lumen of ampulla usually filled with sperm, ental duct connected to oesophagus. Ampulla usually making a sharp bend caudad towards oesophagus at about half its length. Spermathecae 100–150 µm long, 30–50 µm wide at widest part of ampulla. Ectal pore surrounded by compact, roundish mass of gland cells; whole glandular body 45–110 µm in diameter at its widest part. One midventral subneural gland in XIV, 60–90 µm long.

#### Geographical distribution.

Genetically identified from the United Kingdom. Also reported and briefly described from Iceland (Erséus 1976).

#### Remarks.

Our measurements of the coelomic corpuscles (5–7 µm long) contradict the original description where they are described as being 20–28 µm. This could either be due to a high degree of variation in this trait or that we are comparing non-homologous cell types. The smaller subneural gland in XV described by Stephenson could not be distinguished, either because of its small size or because it was absent. Despite a few discrepancies from the original description the small body size of the worms together with the shape of the spermathecae and other reproductive organs supports that the sampled specimens belong to *L. pumilio*.

**Figure 12.**
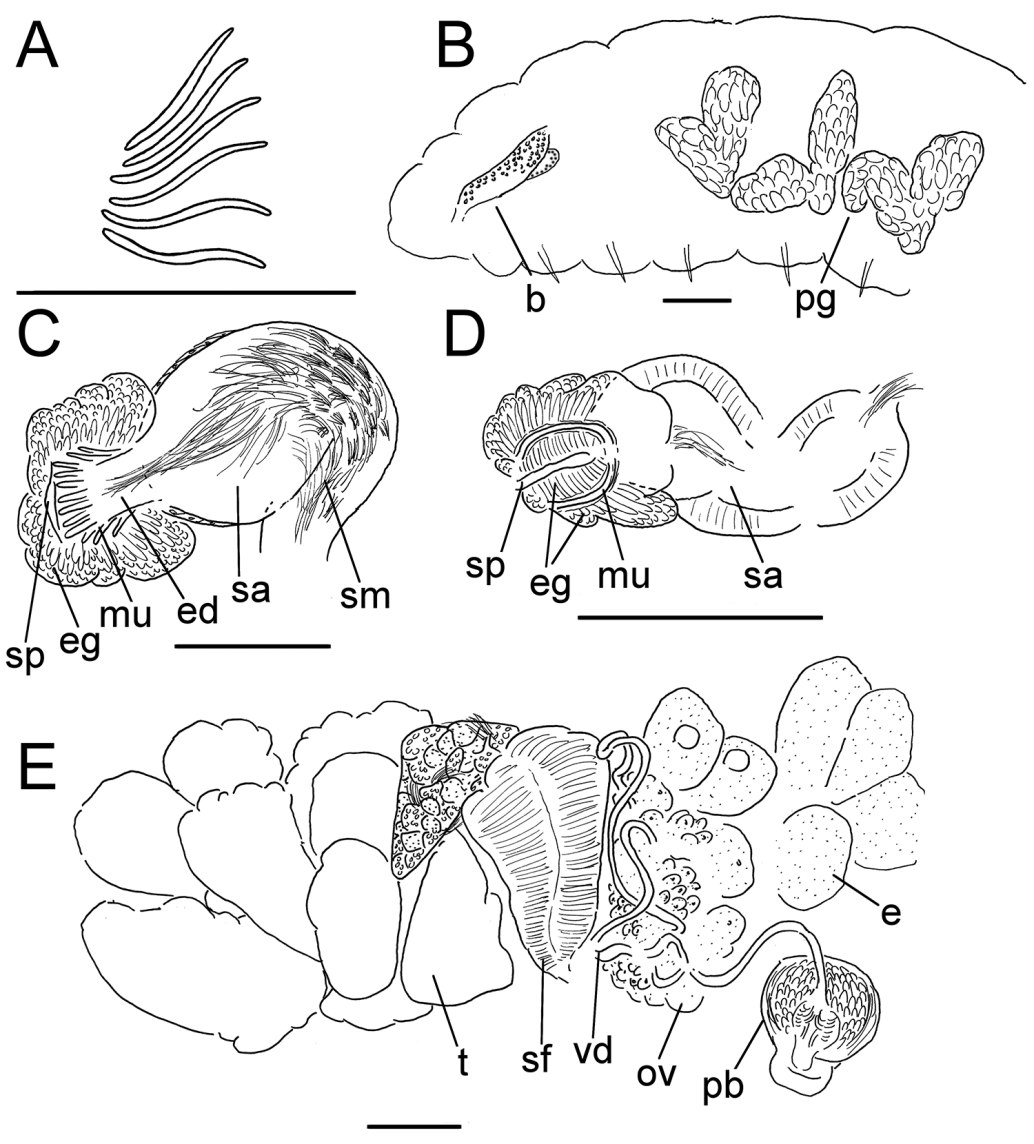
*Lumbricillus rubidus*. **A** Chaetal bundle **B** Anterior body **C**, **D** Spermatheca. **E** Other genitalia. Abbreviations under general notes. Scale bars: 100 µm.

*Lumbricillus pumilio* is genetically closely related to *L. rubidus* Finogenova & Streltsov, 1978 (Fig. 1), with which it also shares morphological similarities. However, *L. pumilio* is generally smaller than *L. rubidus*, and it does not have an as distinct muscular covering of the spermathecal ectal duct as that of the latter (compare Figs 11 and 12).

### Lumbricillus rubidus

Taxon classificationAnimaliaEnchytraeidaEnchytraeidae

Finogenova & Streltsov, 1978

Fig. 12

Lumbricillus rubidus Finogenova & Streltsov, 1978: pp. 17–23, fig. 1; Kossmagk-Stephan 1983: p. 8; Klinth et al. 2017.
Lumbricillus enteromorphae ; sensu Kossmagk-Stephan 1985; nec von Bülow, 1957.

#### Type material.

ZIAS 1/42509 (Nomenclatura Oligochaetologica). Type locality: Dal’nii Plyazh in Dal’nie Zelentsy Bay, Murmansk, Russia (Finogenova and Streltsov 1978). Not studied.

#### Material examined.

SMNH 152792 (CE2549), SMNH 152793 (CE2551), SMNH 152794 (CE2553), SMNH 152795 (CE6105), SMNH 152796 (CE6106), SMNH 152797 (CE6107) & SMNH 152798 (CE6108), seven mature specimens from Sweden. For information on specimen collection localities and GenBank accession numbers see Appendix 1.

#### Description.

Pale to pinkish worms. Length (fixed worms) more than 2.2–3.9 mm (amputated specimens), first 15 segments 2.0–3.1 mm long, width at clitellum 0.31–0.68 mm. More than 17–23 segments. Chaetae slightly sigmoid (Fig. 12A). Dorsal bundles with 3–7 chaetae anterior to clitellum, 3–6 chaetae in postclitellar segments. Ventral bundles with 3–8 chaetae anterior to clitellum, 3–8 chaetae posteriorly. Each worm’s longest measured chaetae 45–50 µm long and 3–5 µm wide. Clitellum extending over XII–1/2XIII. Head pore not observed. Epidermis with transverse rows of gland cells.

Coelomocytes numerous, 10–20 µm long, round, oval or spindle-shaped. Paired pharyngeal glands present in IV, V and VI; each pair converging dorsally (Fig. 12B). Dorsal vessel originating in XIII. One nephridium observed in X, pear-shaped, about 80 µm long, narrowing posteriorly. Anteseptale small, consisting of funnel only. Efferent duct originating at mid length. Brain widening posteriorly, but exact shape uncertain.

Male genitalia paired (Fig. 12E). Testes originating in XI, extending forwards into X, sometimes IX, with testis sacs forming regular club-shaped lobes. Sperm funnels in XI, 135–265 µm long, 85–170 µm wide making them about 1.5–2 times longer than wide, funnels tapering towards vasa deferentia. Most of vasa irregularly coiled in XII, and 10–20 µm wide. Penial bulbs round, 70–130 µm in diameter. Ovaries in XII. One to five mature eggs present at a time.

Spermathecae (Fig. 12C, D) in V, spindle-shaped, without distinct ampulla. Ectal duct short, about 1/5 of total length of spermatheca, rapidly widening into ampulla. Conspicuous muscle cells encircling duct and connecting it to epidermis. Ampulla making sharp bend inwards and entally connecting with oesophagus. Sperm tightly packed in ectal duct, possibly covered by thin layer of secretion, spermatozoan tails occupying outer part of ampulla, heads aggregating into distinct clusters in inner part, and partly embedded in wall, of ampulla. Spermathecae 120–275 µm long, 60–110 µm wide at widest part of ampulla. Gland cells surrounding ectal pore, forming compact body with few marginal lobes, glandular body 60–135 µm in diameter at its widest part. One midventral subneural gland in XIV, 60–150 µm long.

#### Geographical distribution.

Described from Russia and Germany, now genetically identified from Sweden.

#### Remarks.

The specimens in this study match the original description of *L. rubidus* by Finogenova and Streltsov (1978) in most characters such as length, number of chaetae and sperm funnel ratio. It seems that our specimens in general possessed larger internal organs, such as sperm funnels, penial bulbs and spermathecae. Nevertheless, the strong musculature around the ectal pore of the spermathecae, originally described as a muscular bulb, was found with clear resemblance in our specimens. This muscular sleeve covering the ectal duct of the spermathecae is conspicuous in all specimens. Several separate muscle bundles radiate around the base of the duct connecting to the body wall and may have a function in widening the pore in conjunction with copulation or fertilization of eggs. In one specimen, where one spermatheca was seen from above, the layers of musculature created a circle seemingly dividing the ectal gland in two. A closer examination revealed that the musculature more probably is tightly encircling the gland cells of the ectal gland without dividing them. The muscular bulb, as originally termed by Finogenova and Streltsov, is probably the same ectal part of the ectal gland, separated by the encircling musculature, rather than a compact mass of muscles. Similar muscle structures encircling the ectal pore of the spermathecae have been seen in most species of *Lumbricillus* during this study but they never appeared so conspicuous as in *L. rubidus*.

In 1959, Nielsen and Christensen classified the species *L. enteromorphae* von Bülow, 1957 as a hunger form of *L. rivalis*. Kossmagk-Stephan (1985) rejected this idea and instead synonymized *L. rubidus* with von Bülow’s species based on the fact that they both have muscular bulbs. We agree that *L. enteromorphae* should be considered a separate species from *L. rivalis*, because of the asymmetrical ectal glands of the spermathecae and the atrium-like part where the vasa deferentia meets the penial bulbs, observed only in the former species. However, we do not agree that *L. rubidus* is a synonym of *L. enteromorphae*, as the former has sperm funnels that are much shorter in relation to their width (2–4 compared to 8 times longer than wide) and lacks any atrium-like part of the vasa deferentia. It is true that von Bülow described a funnel-like thickening of the spermathecal ectal duct which could be a structure similar to the muscular bulb seen in *L. rubidus*. However, we have observed varied extents of muscular coverings of the ectal ducts in most species belonging to the *lineatus* group, and a true comparison of this character can only be made once we have specimens of von Bülow’s species.

*Lumbricillus rubidus* is genetically closely related to *L. pumilio* (Fig. 1), and shares morphological similarities with both *L. pumilio* and *L. kaloensis* (see remarks for each species respectively).

### Lumbricillus fennicus

Taxon classificationAnimaliaEnchytraeidaEnchytraeidae

Nurminen, 1964

Fig. 13

Lumbricillus fennicus Nurminen, 1964: pp. 48–51, fig. 2; Graefe and Schmelz 1999: p. 61; Rota and Healy 1999: p. 54; Erséus et al. 1999; Klinth et al. 2017.

#### Type material.

HUZM (Nomenclatura Oligochaetologica). Type locality: Tvärminne, Finland (Nurminen 1964). Not seen.

#### Material examined.

SMNH 152729 (CE2767), SMNH 152730 (CE2768), SMNH 152731 (CE2988) & SMNH 152732 (CE6092), four mature specimens from Sweden. For information on specimen collection localities and GenBank accession numbers see Appendix 1.

#### Description.

**Figure 13.**
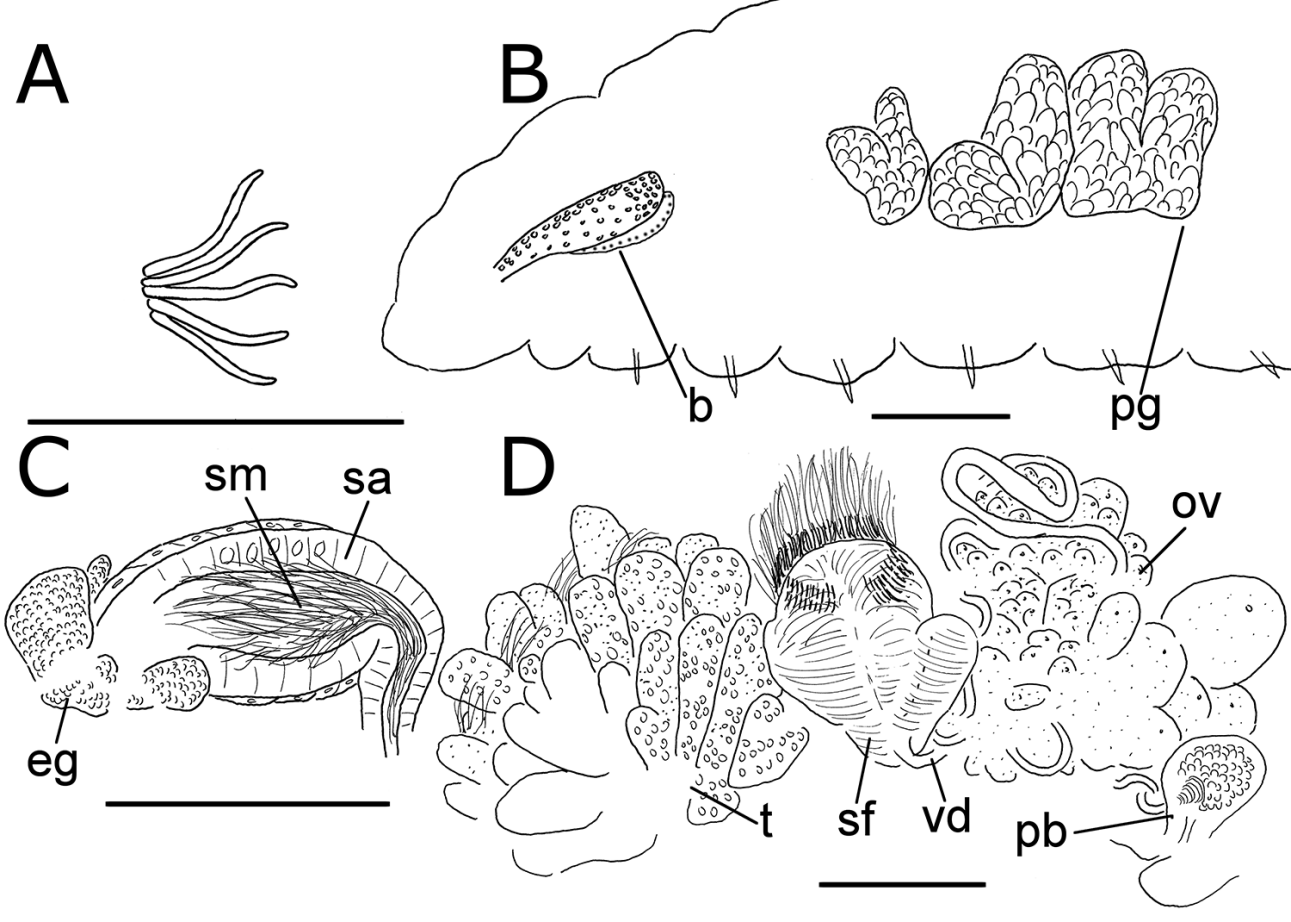
*Lumbricillus fennicus*. **A** Chaetal bundle **B** Anterior body **C** Spermatheca **D** Other genitalia. Abbreviations under general notes. Scale bars: 100 µm.

Colour of worms unknown. Length (fixed worms) more than 1.8–3.5 mm (amputated specimens), first 15 segments 2.0–2.3 mm long, width at clitellum 0.4–0.48 mm. More than 12–23 segments. Chaetae slightly sigmoid (Fig. 13A). Dorsal bundles with 3–5, rarely 2 or 7, chaetae anterior to clitellum, 2–5 chaetae in postclitellar segments. Ventral bundles with 3–7, usually 4–5, chaetae anterior to clitellum, 4–5 chaetae posteriorly. Each worm’s longest measured chaetae 35–50 µm long and about 2.5 µm wide. Clitellum extending over XII–1/2XIII, with granulated and hyaline cells irregularly distributed. Head pore not observed. Epidermis with transverse rows of gland cells.

Coelomocytes numerous, 15–25 µm long, round, oval or spindle-shaped. Paired pharyngeal glands present in IV, V and VI; each pair converging dorsally (Fig. 13B). Dorsal vessel originating in XIII. Nephridia observed in XIII–XVI about 45 µm long, anteseptale consisting of funnel only, efferent duct originating at mid length of postseptale. Brain widening posteriorly, with posterior incision creating two hornlike structures.

Male genitalia paired (Fig. 13D). Testes originating in XI, extending forwards into X, sometimes IX, with testis sacs forming regular club-shaped lobes. Sperm funnels in XI, 100–125 µm long, 95–110 µm wide making them about 1–1.5 times longer than wide. Funnels lobed rather than cylindrical, and abruptly tapering towards vasa deferentia. Vasa with few irregular coils around ovaries in XII, and about 10 µm wide. Penial bulbs round/pear-shaped 45–50 µm in diameter. Three to four mature eggs present at a time.

Spermathecae (Fig. 13C) in V, spindle-shaped, without distinct ampulla. Ectal duct short, encircled by musculature, and rapidly widening into ampulla. Ampulla after maximum width making sharp bend inwards, entally connecting with oesophagus. Sperm evenly embedded in wall of ampulla, filling but not embedded in ental duct. Spermathecae 170–195 µm long, 45–65 µm wide at widest part of ampulla. Gland cells surrounding ectal pore, forming compact mass, somewhat lobed, whole glandular body 60–75 µm in diameter at its widest part. Midventral subneural glands in XIII, XIV and in one specimen in XV, measuring 45–70 µm, 75–90 µm and 70 µm respectively.

#### Geographical distribution.

Originally described from Finland, but also reported from Denmark, France (Lafont and Vivier 2006), Germany (Giere 1976), Ireland (Healy 2007), Norway and Sweden, now genetically identified from Sweden.

#### Remarks.

The original description of *L. fennicus* matches the specimens of this study in most characters, but there are a few differences. Our specimens measured 2–3.5 mm in length after fixation, but considering that some had been cut directly posterior to the clitellum the length of the complete worms probably was 3–5 mm. This is smaller than the 8 mm reported by Nurminen (1964), but he based his description on living worms. Nurminen described the clitellum as covering 1/2XI–XII while in our specimens, the clitellum extends over XII–1/2XIII. The extension of the clitellum may vary as it develops, but the whole structures does not generally shift in position, and this suggests the possibility of a printing or observation error in the original description.

The lobed, as opposed to cylindrical, sperm funnels are so far (in European species) only reported for *L. fennicus*, and this, together with the matching shape of the spermathecae, allowed confident allocation of the specimens to this species despite some incongruence among the characters mentioned above. The interpretation of the lobes of the sperm funnels probably also differs between living and fixed specimens. Our Swedish specimens were collected in freshwater habitats, but the sites are possibly subjected to brackish water at times, making the range of salinity similar to the original records from the Gulf of Finland. Most other records in Europe are from coastal oligohaline or inland freshwater habitats.

*Lumbricillus fennicus* is both genetically (Fig. 1) and morphologically placed within the *L. lineatus* group.

### The *pagenstecheri* group

*Characteristics*: Testes with testis sacs regularly lobed in bunch-like arrangement. Spermathecae with distinct ampulla and glands both surrounding the ectal pore and distributed along the duct. Chaetae 3–6 per bundle, or more; upper bundles dorsolateral. Penial bulbs round. Sperm funnels about twice as long as wide.

### Lumbricillus pagenstecheri

Taxon classificationAnimaliaEnchytraeidaEnchytraeidae

(Ratzel, 1869), a species complex

Enchytraeus pagenstecheri Ratzel, 1869: pp. 587–588, pl. XLII, figs 2, 13, 20b & 21.
Pachydrilus pagenstecheri ; Vejdovsky 1877: p. 298; Ditlevsen 1904: pp. 433–434, fig. 29, pl. XVIII, fig. 6; Knöllner 1935: p. 436; Černosvitov 1937: p. 292.
Lumbricillus pagenstecheri ; Ude 1901: p. 9, pl. I, fig. 14; Southern 1909: p. 153; Stephenson 1925: pp. 1315–1316; von Bülow 1957: pp. 77–78, pl. XXV, figs 1–7; Nielsen and Christensen 1959: pp. 104–105, figs 117–120; Erséus 1976: pp. 9–11, fig. 8.
Lumbricillus henkingi Ude, 1901: pp. 9–10, pl. II, figs 15–18; Stephenson 1925: p. 1315.
Lumbricillus ritteri Eisen, 1904: pp. 84–86, figs 53–54, pl. XIII, figs 5–9; Nielsen and Christensen 1959: p. 97; Erséus 1976: pp. 9–10, fig. 12.
Lumbricillus aegialites Stephenson, 1922: pp. 1126–1130, figs 2–3; Stephenson 1924: p. 211; Stephenson 1925: p. 1314.
Lumbricillus necrophagus Stephenson, 1922: pp. 1130–1133, figs 4–5.
Lumbricillus georgiensis Tynen, 1969: pp. 390–391, figs 1–3.

#### Type material.

Typus amissus (Nomenclatura Oligochaetologica). Type locality: The original material was collected in Rhine River near Karlsruhe, and in ponds around Heidelberg, Germany (Ratzel 1869), but none of these places has yet been specifically designated as the type locality. We did not designate a neotype as we do not have material from any of the original localities, nor do we know which one, if any, of our cryptic species that represent the true nominate species.

#### Remarks.

The molecular studies by Klinth et al. (2017) supported the delimitation of four different species with the morphology of *L. pagenstecheri*, here denoted as cryptic species A–D. Particularly the morphology of the spermathecae characterizes this group. There are two groups of gland cells, one creating the typical mass of glands surrounding the ectal pore, as seen in the other species of *Lumbricillus*, and the other group composed of numerous, rather long, gland cells covering the ectal duct. These two groups of gland cells can be difficult to distinguish from each other, depending on the orientation of the mounted specimens, but they create the impression of a very narrow duct followed by a distinct, almost spherical, thin-walled ampulla. While there seems to be some morphological differences between the four species in this study, such as size and number of chaetae, there are too few sampled specimens to verify that these characters do not overlap.

*Lumbricillus pagenstecheri* was originally described by Ratzel (1869) from the Rhine River in Germany and has later been re-described by Nielsen and Christensen (1959) as well as others and today includes five synonymized species (listed above). Such synonymies may need reappraisal as there are some differences between the original descriptions concerning size, number of segments and number of chaetae and there is a possibility that some of the synonymized species are present in our material. Moreover, about thirteen described species from the Northwestern Pacific and eight from the Northeastern Pacific have a morphology similar to that of *L. pagenstecheri* (Timm 2005), and a more extensive phylogenetic study focused on this part of the genus will be necessary, to resolve the taxonomy of this complex group.

For this study, we chose to present the morphological measurements only for our cryptic species A, which is the only one with a sufficient sample size, and provide a comparison of some characters with the other three cryptic species in Table 2. In general, species B and D where the largest, species D possessed fewer chaetae per bundle than the others, and for species C we unfortunately had no fully mature specimens. Full information on collection localities and accession numbers of all four species are given in Appendix 1.

### Lumbricillus pagenstecheri

Taxon classificationAnimaliaEnchytraeidaEnchytraeidae

(Ratzel, 1869) Cryptic species A

Fig. 14 , Table 2

#### Material examined.

SMNH 152766 (CE1896), SMNH 152767 (CE1897), SMNH 152768 (CE1899), SMNH 152769 (CE2497), SMNH 152770 (CE2498) & SMNH 152771 (CE2500), six mature specimens from Sweden. For information on specimen collection localities and GenBank accession numbers see Appendix 1.

#### Description.

**Figure 14.**
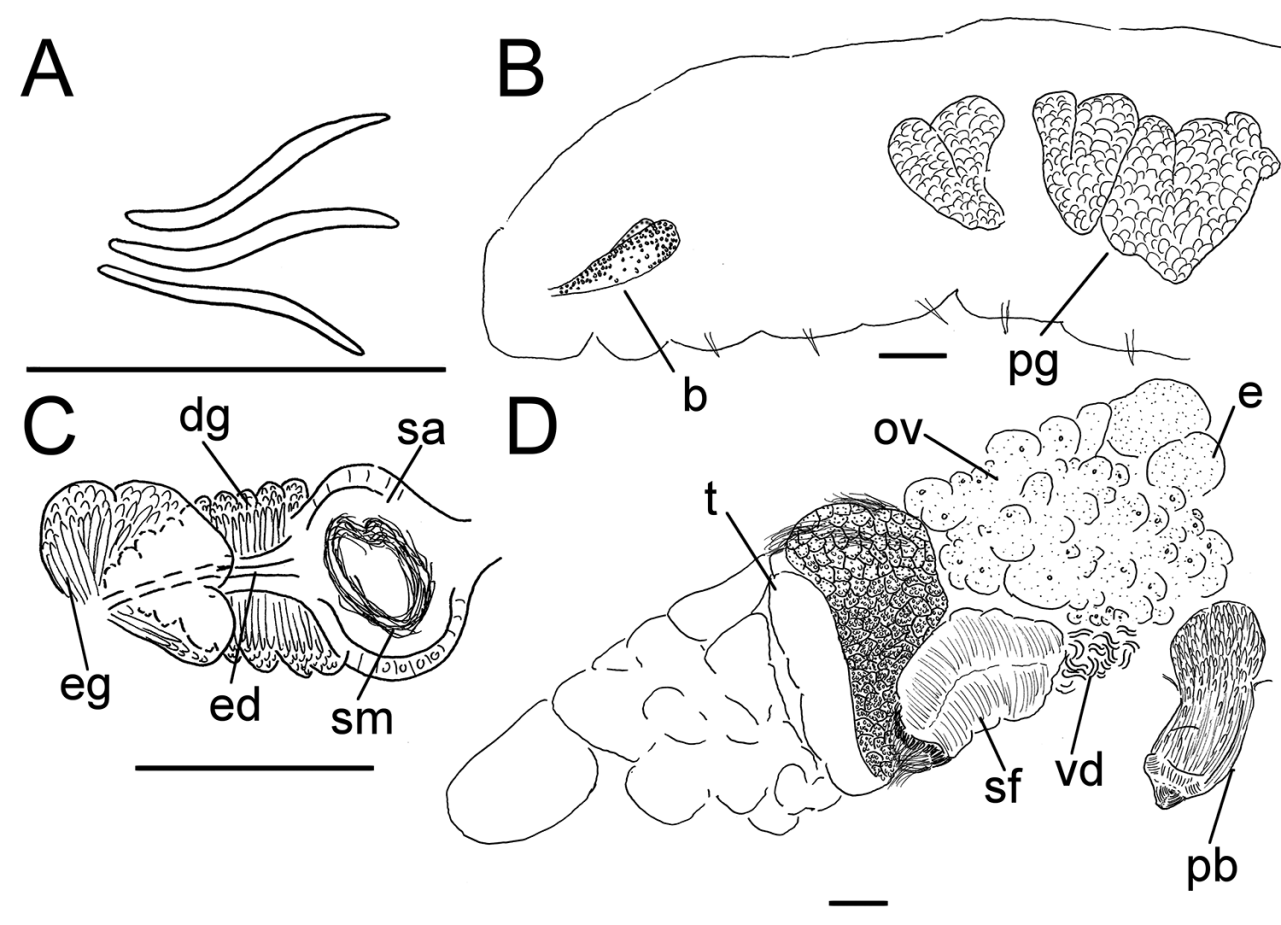
*Lumbricillus pagenstecheri* A. **A** Chaetal bundle **B** Anterior body **C** Spermatheca **D** Other genitalia. Abbreviations under general notes. Scale bars: 100 µm.

White to yellow worms. Length (fixed worms) more than 2.8–9.3 mm (amputated specimens), first 15 segments 2.4–4.2 mm long, width at clitellum 0.59–0.75 mm. More than 17–40 segments. Chaetae sigmoid (Fig. 14A). Dorsal bundles with 2–5 chaetae anterior to clitellum, 2–5(6) chaetae in postclitellar segments. Ventral bundles with (2)4–7 chaetae anterior to clitellum, (2)3–6(8) chaetae posteriorly. Each worm’s longest measured chaetae 70–95 µm long, about 5–8 µm wide. Clitellum extending over XII–XIII. Head pore at 0/1. Epidermis with transverse rows of gland cells.

**Table 2.** Comparison of selected measured traits from the four possibly cryptic species of *L. pagenstecheri*, as well as their known geographical distribution. As our specimens were amputated for the extraction of DNA we could only compare the size of the first 15 segments.

*L. pagenstecheri* cryptic species: distribution	Length I–XV (mm) /Width at clitellum (mm)	Chaetae					Penial bulbs diameter (µm)	Spermathecae		
		Dorsal		Ventral		Length (µm)				
		Preclit.	Postclit.	Preclit.	Postclit.			Length (µm)	Width of ampulla (µm)	Ectal gland diameter (µm)
A: Canada, Denmark, Sweden	2.4–4.2 /0.6–0.8	2–5	2–5(6)	(2)4–7	(2)3–6(8)	70–95	135–185	140–215	75–110	80–195
B: Norway	4.3–5.3 /0.9–1.8	5–6(7)	3–5	(6)7–8	4–6(7)	125–135	365–390	170–265	125–225	260–340
C: Canada, Norway, Spain	2.5–2.7 /0.3–0.7	3–5	2–4	4–6	2–7	65–75	130	110	80	105
D: Canada, Norway	2.5–5.0 /0.6–1.0	3–5	2–3(4)	4–6(7)	2–4	95–110	115–245	180–205	145–170	190–250

Coelomocytes numerous, 15–25 µm long, spindle-shaped, oval, round, granulated with distinct nucleus. Paired pharyngeal glands present in IV, V and VI; two first pairs connected dorsally, third pair with uncertain connection (Fig. 14B). Dorsal vessel originating in XIII. Nephridia observed in VII–X and XII–XXI, 120–130 µm long, anteseptale funnel only, postseptale oval, tapering into posterior efferent duct. Brain with shallow posterior incision.

Male genitalia paired (Fig. 14D). Testes originating in XI, extending forwards into X, sometimes IX, with testis sacs forming regular club-shaped lobes. Sperm funnels in XI, 210–300 µm long, 145–225 µm wide, making them about as long as wide or twice as long as wide, funnels gradually tapering towards vasa deferentia. Most of vasa irregularly coiled in XII, 10–15 µm wide. Penial bulbs round, 135–185 µm in diameter. Ovaries in XII. About two to eight mature eggs present at a time.

Spermathecae (Fig. 14C) in V, club-shaped, with distinct ampulla. Ectal duct narrow, about as long as ampulla, abruptly widening into ampulla. Ampulla round, entally connecting with oesophagus. Sperm arranged in circular masses in ampulla. Spermathecae 140–215 µm long, 75–110 µm wide at widest part of ampulla. Two groups of gland cells, one covering ectal duct, the other surrounding ectal pore. Gland cells surrounding ectal pore forming compact mass, slightly lobed, whole glandular body 80–195 µm in diameter at its widest part. Two midventral subneural glands in XIII–XIV, 130 µm and 95 µm long, respectively.

#### Geographical distribution including BOLD data.

Genetically identified from Sweden; also recognized from Canada and Denmark (BIN-number: BOLD:AAF9627).

### Lumbricillus viridis

Taxon classificationAnimaliaEnchytraeidaEnchytraeidae

Stephenson, 1911

Fig. 15

Lumbricillus viridis Stephenson, 1911: pp. 46–50, figs 6a–b & 7a–c; Nielsen and Christensen 1959: pp. 103–104, fig. 116; Klinth et al. 2017.
Pachydrilus orthochaetus Delphy, 1921: pp. 64–82, figs 29–41.

#### Type material.

Typus amissus (Nomenclatura Oligochaetologica). Type locality: Firth of Clyde, Wemyss Bay, United Kingdom (Stephenson, 1911). We did not designate a neotype as we do not have material from the type locality.

#### Material examined.

ZMBN 107933 (CE12037), ZMBN 107934 (CE12038), ZMBN 107935 (CE12039) & ZMBN 107938 (CE23255), three mature and one half-mature specimens from Norway. For information on specimen collection localities and GenBank accession numbers see Appendix 1.

#### Description.

**Figure 15.**
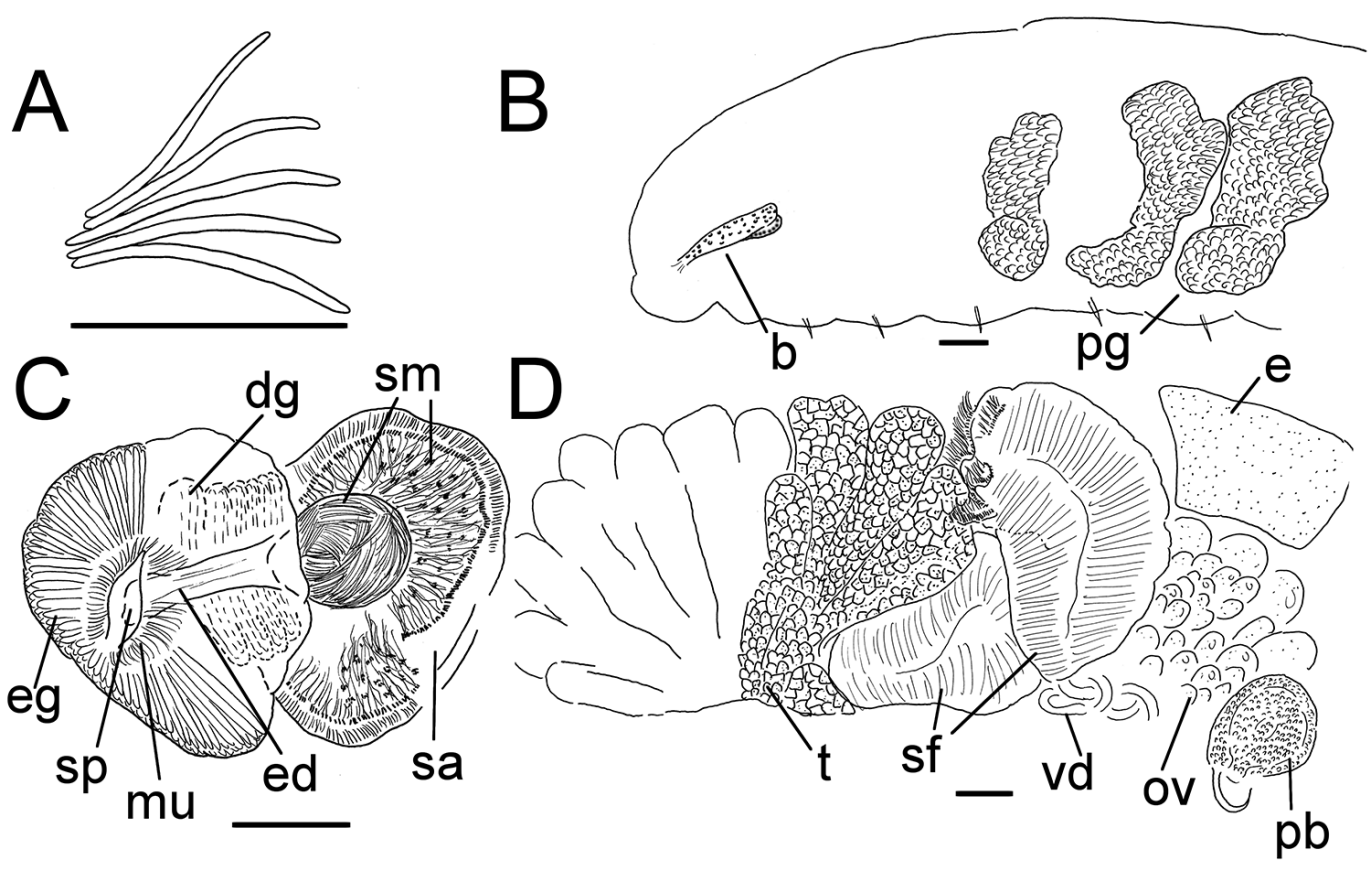
*Lumbricillus viridis*. **A** Chaetal bundle **B** Anterior body **C** Spermatheca **D** Other genitalia. Abbreviations under general notes. Scale bars: 100 µm.

Green worms (sometimes yellowish-green). Length (fixed worms) more than 7.9–10.6 mm (amputated specimens), first 15 segments 3.8–6.2 mm long, width at clitellum 0.74–1.05 mm. More than 23–41 segments. Chaetae straight or slightly sigmoid (Fig. 15A). Dorsal bundles with 3–6 chaetae anterior to clitellum, 3–5 chaetae in postclitellar segments. Ventral bundles with 3–6 chaetae anterior to clitellum, 3–5 chaetae posteriorly. Each worm’s longest measured chaetae 70–85 µm long, about 5 µm wide. Clitellum extending over XII–XIII. Head pore at 0/1. Epidermis with transverse rows of gland cells.

Coelomocytes numerous, 20–35 µm long, spindle-shaped, oval, round, granulated. Paired pharyngeal glands present in IV, V and VI; each pair converging dorsally (Fig. 15B). Dorsal vessel originating in XIII. Nephridia observed in VIII–X and XV–XIX, about 250 µm long, anteseptale funnel only, postseptale oval, tapering into posterior efferent duct. Brain with posterior incision.

Male genitalia paired (Fig. 15D). Testes originating in XI, extending forwards into X, with testis sacs forming regular club-shaped lobes. Sperm funnels in XI, 620–670 µm long, 320–350 µm wide, making them about twice as long as wide, funnels tapering towards vasa deferentia. Most of vasa irregularly coiled in XII, 25–30 µm wide. Penial bulbs round, 170–180 µm in diameter. Ovaries in XII. About five mature eggs present at a time.

Spermathecae (Fig. 15C) in V, club-shaped, with distinct ampulla. Ectal duct narrow, shorter than ampulla, abruptly widening into ampulla. Ampulla round. Sperm arranged in a compact central sphere in the ampulla as well as embedded in the wall of ampulla, creating a circle around the sphere. Spermathecae 265–320 µm long, 270–310 µm wide at widest part of ampulla. Gland cells surrounding ectal pore, forming compact mass, slightly lobed, whole glandular body 310–325 µm in diameter at its widest part. Gland cells also along the ectal duct. Up to four midventral subneural glands in XIV–XVII, 240–270 µm, 215–245 µm, 190–215 µm and 130 µm long, respectively; glands in XVII not observed in all specimens.

#### Geographical distribution including BOLD data.

Genetically identified from France and Norway. Previously described from Denmark, Norway (Nurminen 1965b) the United Kingdom and Sweden (Erséus 1977). BIN-number: BOLD:AAU1636.

#### Remarks.

Specimens in this study are smaller and possess somewhat fewer chaetae, than the ones from the original description by Stephenson and the later re-description by Nielsen and Christensen. Furthermore, the observed proportions of the sperm funnels (twice longer than wide) differ greatly from those (7–10:1, or 6–8:1) described by Stephenson and Nielsen and Christensen, respectively. However, folding of these organs may have caused us to underestimate their true length. Nevertheless, the distinct greenish colour of the sampled specimens and the resemblance between their spermathecae and particularly the one described by Nielsen and Christensen confirm these specimens as *Lumbricillus viridis*.

According to our knowledge, the presence of gland cells along the spermathecal ectal duct has not been reported for *L. viridis* before, possibly because of the difficulty of distinguishing these gland cells from the large ones surrounding the ectal pore. In this study, similar duct glands have only been observed in *L. pagenstecheri* sensu lato.

*Lumbricillus viridis* is genetically most closely related to the *L. pagenstecheri* species complex (Fig. 1: *L. pagenstecheri* A–D).

### The “*tuba*” group

*Characteristics*: Testes with testis sacs regularly lobed in bunch-shaped arrangement. Spermathecae with ampulla distinctly set off from the duct and glands surrounding the ectal pore. Chaetae usually 3–6 per bundle; upper bundles dorsolateral. Penial bulbs round. Sperm funnels about as long as wide.

Note that this group, containing also *L. scandicus* sp. n., is not monophyletic (Fig. 1); it is based on morphological similarity only.

### Lumbricillus tuba

Taxon classificationAnimaliaEnchytraeidaEnchytraeidae

Stephenson, 1911

Fig. 16

Lumbricillus tuba Stephenson, 1911: pp. 42–46, figs 5a–b, pl. I, figs 6–8; Nielsen and Christensen 1959: p. 105, fig. 131; Erséus et al. 2010; Klinth et al. 2017.

#### Type material.

Typus amissus (Nomenclatura Oligochaetologica). Type locality: Firth of Clyde, Millport, Island of Cumbrae, United Kingdom (Stephenson 1911). We did not designate a neotype as we do not have material from the type locality.

#### Material examined.

ZMBN 107916 (CE22614), one mature specimen from Norway. For information on specimen collection locality and GenBank accession number see Appendix 1. Note that this specimen is the only sexually mature available for this study, but additional (immature) worms were studied genetically by Klinth et al. (2017).

#### Description.

**Figure 16.**
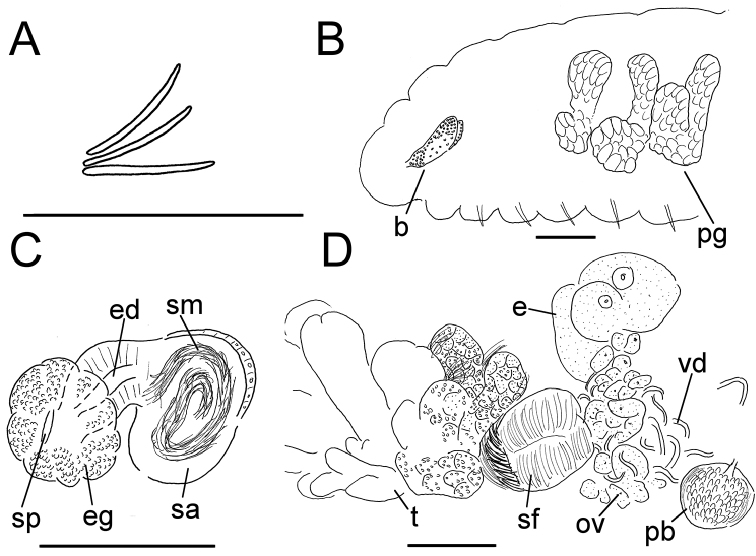
*Lumbricillus tuba*. **A** Chaetal bundle **B** Anterior body **C** Spermatheca **D** Other genitalia. Abbreviations under general notes. Scale bars: 100 µm.

White to grey worm. Length (fixed worm) more than 4.8 mm (amputated specimen), first 15 segments 2.0 mm long, width at clitellum 0.39 mm. More than 39 segments. Chaetae slightly sigmoid (Fig. 16A). Dorsal bundles with 2–3 chaetae anterior to clitellum, 2–3 chaetae in postclitellar segments. Ventral bundles with 3–4 chaetae anterior to clitellum, 2–3 chaetae posteriorly. The worm’s longest measured chaeta 48 µm long, about 3 µm wide. Clitellum extending over XII–1/2XIII. Head pore not observed. Epidermis with transverse rows of gland cells.

Coelomocytes numerous, 20 µm long, spindle-shaped, oval, round, granulated. Paired pharyngeal glands present in IV, V and VI (Fig. 16B). Dorsal vessel originating in XII. Nephridia not observed. Brain longer than wide, further shape unclear.

Male genitalia paired (Fig. 16D). Testes originating in XI, extending forwards into IX, with testis sacs forming regular club-shaped lobes. Sperm funnels in XI, 120 µm long, 105 µm wide, making them slightly longer than wide, funnels tapering towards vasa deferentia. Most of vasa irregularly coiled in XII, 10 µm wide. Penial bulbs round, 85 µm in diameter. Ovaries in XII. Two mature eggs present.

Spermathecae (Fig. 16C) in V, club-shaped, with distinct ampulla. Ectal duct as long as ampulla, abruptly widening into oval ampulla. Sperm circularly arranged in ampulla. Spermathecae 120 µm long, 85µm wide at widest part of ampulla. Gland cells surrounding ectal pore, forming compact mass, whole glandular body 75 µm in diameter at its widest part. Three midventral subneural glands in XIII– XV, 85µm, 90 µm and 85 µm long, respectively.

#### Geographical distribution including BOLD data.

Genetically identified from Norway and Sweden. Also described from Denmark and the United Kingdom. BIN-number: BOLD:ACQ1913.

#### Remarks.

Our specimen matches the original description by Stephenson (1911) well in most characters, including the shape of the spermathecae and the proportions of the sperm funnels. However, its body size was smaller and the chaetae per bundle slightly fewer than in the original description.

*Lumbricillus tuba* is genetically most closely related to the *L. pagenstecheri* group (including *L. viridis*), but it is morphologically most similar to *L. scandicus* sp. n. described below.

### Lumbricillus scandicus sp. n.

Taxon classificationAnimaliaEnchytraeidaEnchytraeidae

http://zoobank.org/A45F3597-1CA9-40D1-96C8-D4034588A6A6

Fig. 17

Lumbricillus cf. helgolandicus Nielsen and Christensen 1959: pp. 102–103, fig. 115; Finogenova and Timm 1988: 97–99, figs 6–10; Klinth et al. 2017.
Lumbricillus helgolandicus sensu von Bülow 1957: p. 79, pl. XXV, figs 11–12, pl. XXIX, figs 5–6; Tynen and Nurminen 1969: p. 152, fig. 1i.
Non Pachydrilus helgolandicus Michaelsen, 1927: p. 12, fig. 11; Michaelsen 1934: pp. 135–141, fig. 1.

#### Holotype.

SMNH Type-8923 [former SMNH 152721] (CE1905), a whole-mounted voucher of a sexually mature and DNA-barcoded worm (COI barcode is KU893950 in NCBI/GenBank; Klinth et al. 2017).

#### Type locality.

Sweden, Öland, Borgholm, Neptuni Åkrar, beach with mixed shelly sand, pebbles and organic material, 57.3346 N, 17.0102 E, collected 11 June 2006 by L. Matamoros.

#### Paratype.

SMNH Type-8925 [former SMNH 152722] (CE1907), a whole-mounted sexually mature specimen from the type locality.

#### Other material examined.

SMNH 152720 (CE975), SMNH 152723 (CE1915), SMNH 152724 (CE2548) & SMNH 152725 (CE2552), four mature specimens from Sweden. For information on specimen collection localities and GenBank accession numbers see Appendix 1.

#### Etymology.

Named after Scandinavia where the species has been found.

#### Diagnosis.

This species is morphologically most similar to *L. helgolandicus* and *L. tuba*. It is distinguished from *L. helgolandicus* in having shorter sperm funnels, sperm arranged circularly in the spermathecae and generally possessing more chaetae per bundle. *Lumbricillus scandicus* can be distinguished from *L. tuba* in having spermathecal ectal glands that are larger than the ampulla and generally possessing more chaetae per bundle.

#### Description of all material.

**Figure 17.**
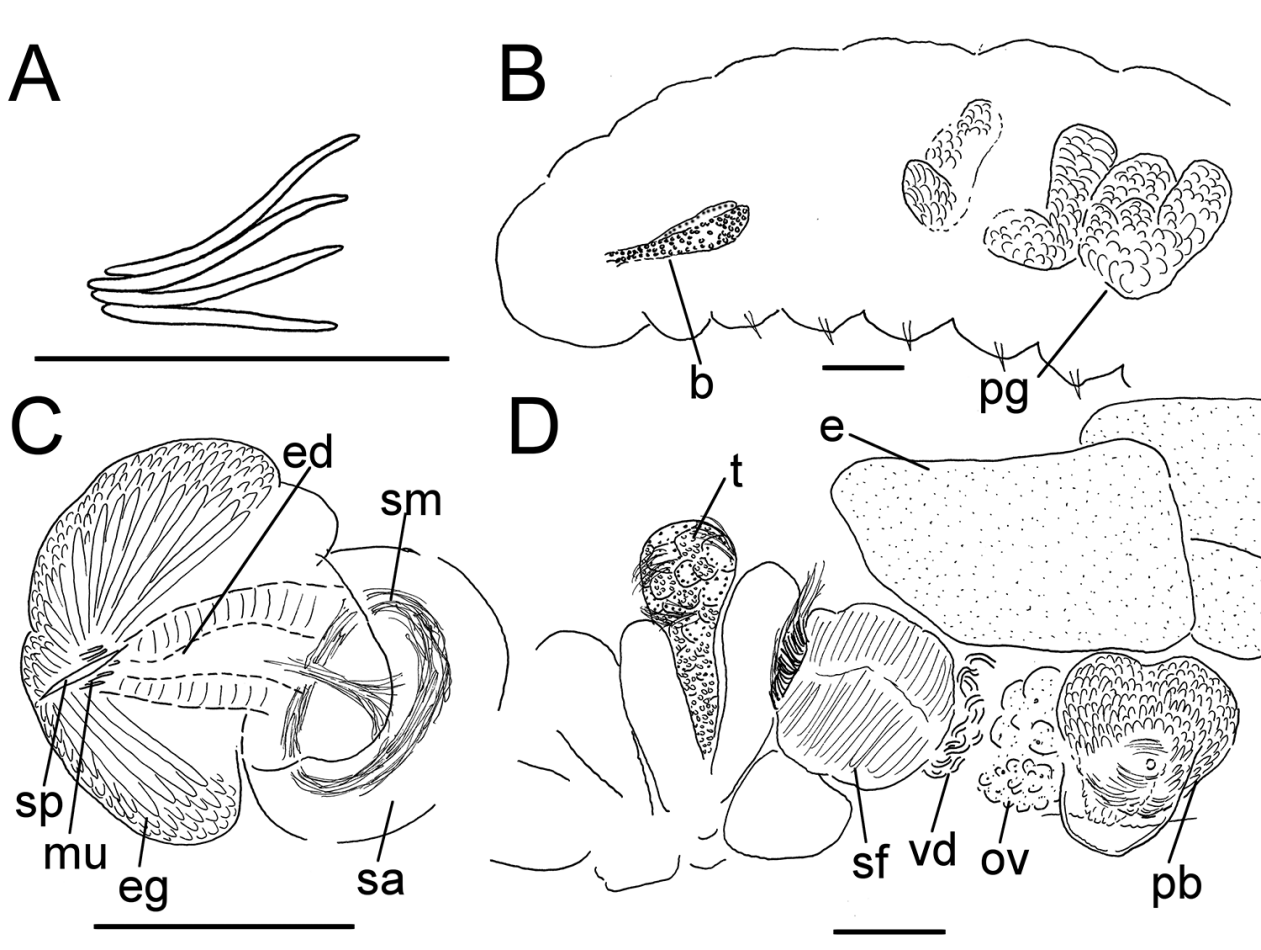
*Lumbricillus scandicus* sp. n. **A** Chaetal bundle. **B** Anterior body **C** Spermatheca **D** Other genitalia. Abbreviations under general notes. Scale bars: 100 µm.

Pale, white to pinkish or orange worms. Length (fixed worms) more than 2.6–3.9 mm (amputated specimens), first 15 segments 2.0–2.9 mm long, width at clitellum 0.3–0.7 mm. More than 18–24 segments. Prostomium hemispherical, sometimes triangular. Chaetae slightly sigmoid (Fig. 17A). Dorsal bundles with 3–6, usually 4–5, chaetae anterior to clitellum, 2–5 chaetae in postclitellar segments. Ventral bundles with 4–7 chaetae anterior to clitellum, 3–6 chaetae posteriorly. Each worm’s longest measured chaetae 50–60 µm long and about 2.5 µm wide. Clitellum extending over XII–1/2XIII, with granulated and hyaline cells irregularly distributed. Head pore not observed. Epidermis with transverse rows of gland cells.

Coelomocytes numerous, 15–20 µm long, round or oval. Paired pharyngeal glands present in IV, V and VI; each pair converging dorsally, with large ventral lobes (Fig. 17B). Posteriormost pair sometimes extending into VII. Dorsal vessel originating in either XII or XIII, difficult to distinguish due to presence of mature eggs. One nephridium observed in XIV about 85 µm long, anteseptale consisting of funnel only, duct originating posteroventrally. Brain widening posteriorly, possibly with posterior incision.

Male genitalia paired (Fig. 17D). Testes originating in XI, extending forwards into X, sometimes IX, with testis sacs forming regular club-shaped lobes. Sperm funnels in XI, 95–205 µm long, 90–160 µm wide making them about 1–1.5 times longer than wide. Funnels cylindrical, abruptly tapering towards vasa deferentia. Vasa with few irregular coils around ovaries in XII, and about 7–10 µm wide. Penial bulbs round/pear-shaped 75–145 µm in diameter. Two to six mature eggs present at a time.

Spermathecae (Fig. 17C) in V, club-shaped, with ampulla distinctly set apart from ectal duct. Ectal duct wall with long cylindrical cells. Ampulla sub-spherical, thin-walled, entally communicating with oesophagus. Sperm following duct to ampulla, in ampulla aggregated into central mass haloed by circle of spermatozoa. Spermathecae 90–160 µm long, 65–115 µm wide at widest part of ampulla. Gland cells surrounding ectal pore, forming compact, slightly folded mass, 100–155 µm in diameter at its widest part. Up to three midventral subneural glands in XIII– XV, 85–90 µm, 90–140 µm and 70–100 µm long, respectively; glands in XIII and XIV not observed in all specimens.

#### Details of holotype.

Length 3.5 mm (amputated specimen), first 15 segments 2.7 mm long, width at clitellum 0.5 mm. More than 20 segments. Dorsal bundles with 3–5, chaetae anterior to clitellum, 3–4 chaetae in postclitellar segments. Ventral bundles with 4–7 chaetae anterior to clitellum, 3–5 chaetae posteriorly. Longest chaetae about 60 µm long and about 2.5 µm wide.

Coelomocytes about 20 µm long.

Sperm funnels about 155 µm long and 160 µm. Vasa deferentia about 7 µm wide. Penial bulbs 145 µm in diameter. Four mature eggs present.

Spermathecae (Fig. 17C) 120 µm long, 80 µm wide at widest part of ampulla. Gland cells surrounding ectal pore 155 µm in diameter at its widest part. Three midventral subneural glands in XIII–XV, 85 µm, 105 µm and 85 µm long, respectively.

#### Geographical distribution.

Genetically identified from Norway and Sweden. Also reported from Denmark and Russia (White Sea).

#### Remarks.

The new species corresponds well to the description of Lumbricillus cf. helgolandicus (Michaelsen, 1927) by Nielsen and Christensen (1959), which is why this name was used in the molecular study by Klinth et al. (2017). However, Nielsen and Christensen noted several differences in the morphology of their specimens in comparison to the extended description of *L. helgolandicus* given later by Michaelsen (1934), the most important being the morphology of the spermathecae and the sperm funnels. The spermathecal ampulla was interpreted by Michaelsen as being filled with an irregular mass of spermatozoa. Von Bülow (1957) instead redrew the spermathecae as having a distinct circle of spermatozoa which also corresponds to the interpretation by Nielsen and Christensen and what we observed in this study. Furthermore, Michaelsen originally described the sperm funnels as 12 times longer than wide. Nielsen and Christensen, on the other hand, found the funnels to be only 2–3 times longer than wide, which corresponds better to the ratio measured in our material. von Bülow, 1957, unfortunately did not comment on the length/width ratio of the funnels.

These circumstances prompted re-examination of the last remaining syntype of *Pachydrilus helgolandicus* from Michaelsen’s collection in the Zoological Museum in Hamburg (see description of that material below). We found that the sperm funnels were more than 4 times longer than wide, compared to his reported 12 times. This difference could be explained by Michaelsen having examined live material, whereas the syntype that we studied had been fixed (contracted) in formalin or alcohol, shortening the sperm funnels. Furthermore, we might have underestimated the true length of the sperm funnels due to the difficulties with measuring folded organs in mounted material. Regardless, compared to our material of “L. cf. helgolandicus”, here described as *L. scandicus* sp. n., the sperm funnels of *L. helgolandicus* sensu stricto clearly have a higher length/width ratio.

The spermathecae of *L. helgolandicus* are similar to those of *L. scandicus* in having a distinct ampulla and a very large ectal gland. However, in *L. helgolandicus*, the spermatheca contains sperm that are arranged in an irregular mass, and it has a very distinct musculature covering the ectal duct (possibly made more apparent by the aging of the material), whereas the spermatheca of *L. scandicus* has sperm arranged in a more circular manner and only weakly defined musculature covering the ectal duct.

*Lumbricillus helgolandicus* is larger than *L. scandicus* and has generally larger internal organs. It also has fewer chaetae per bundle, no more than 5 in preclitellar, and 2–3 in postclitellar bundles, whereas *L. scandicus* has up to 7 chaetae in preclitellar, and up to 6 in postclitellar bundles.

Nielsen and Christensen (1959) examined specimens that seem to have been larger than ours and closer to *L. helgolandicus* in size. However, like our material, they had sperm funnels that were not much longer than wide, more chaetae per bundle and spermathecae with sperm arranged in a circular manner. Similarly, the material that von Bülow (1957) referred to as *L. helgolandicus* also had more chaetae per bundle than Michaelsen’s worm and had spermathecae with sperm arranged in a circular manner.

Based on our assessment of the syntype from Helgoland, we conclude that our Scandinavian material is not conspecific with *L. helgolandicus* (Michaelsen, 1927), and instead deserves to be treated as a new species (*L. scandicus*). Furthermore, we conclude that *L. helgolandicus* sensu von Bülow 1957 and L. cf. helgolandicus sensu Nielsen and Christensen 1959 are identical to *L. scandicus*.

*Lumbricillus scandicus* was genetically found as sister to the *L. lineatus* group (Fig. 1).

### Lumbricillus helgolandicus

Taxon classificationAnimaliaEnchytraeidaEnchytraeidae

(Michaelsen, 1927)

Fig. 18

Pachydrilus helgolandicus Michaelsen, 1927: p. 12, fig. 11; Michaelsen 1934: pp. 135–141, fig. 1.

#### Type material.

ZMH V5786 Zoologisches Museum Hamburg (Michaelsen 1927), syntype, now designated as lectotype. Type locality: Helgoland beach.

#### Description of lectotype.

**Figure 18.**
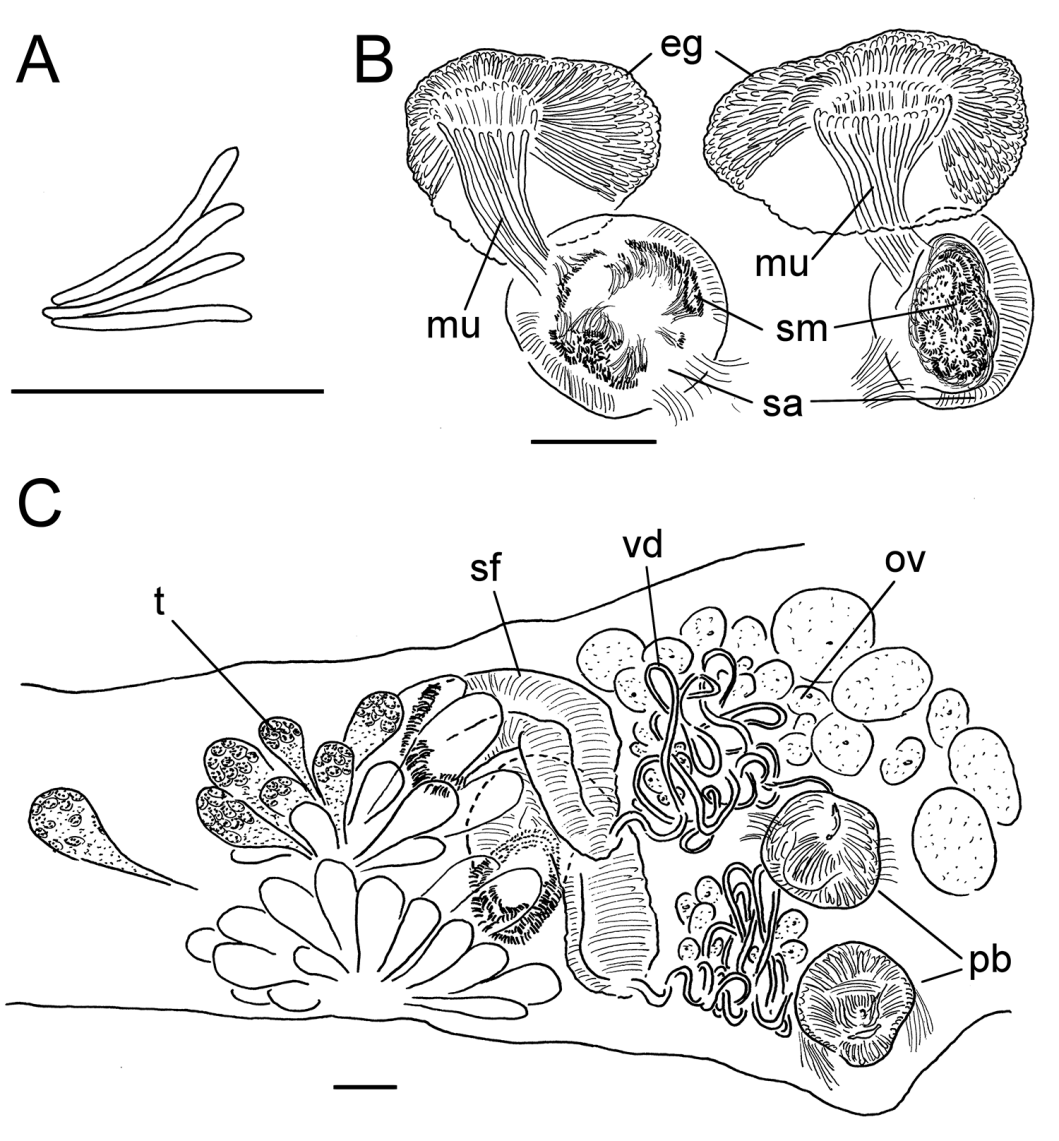
*Lumbricillus helgolandicus*. **A** Chaetal bundle **B** Spermathecae **C** Other genitalia. Abbreviations under general notes. Scale bars: 100 µm.

White worm. Length (fixed) 13 mm, 41 segments; first 15 segments 4.9 mm long, width at clitellum 1.1 mm. Prostomium hemispherical. Chaetae straight to slightly sigmoid (Fig. 18A). Dorsal bundles with 3–5, chaetae anterior to clitellum, 2–3 chaetae in postclitellar segments. Ventral bundles with 3–5 chaetae anterior to clitellum, 2–3 chaetae posteriorly. The worm’s longest measured chaetae 70 µm long and about 7.5 µm wide. Clitellum extending over XII.

Coelomocytes numerous, about 25 µm long, round or oval. Paired pharyngeal glands present in IV, V and VI; each pair converging dorsally, with large ventral lobes. Nephridia observed in XX, XXVI–XXVII, and possibly VIII–X, about 120–130 µm long, anteseptale consisting of funnel only, duct originating posteroventrally.

Male genitalia paired (Fig. 18C). Testes originating in XI, extending forwards into X, and possibly IX, with testis sacs forming regular club-shaped lobes. Sperm funnels in XI, about 685 µm long, 205 µm wide making them about 3.5 times longer than wide. Funnels cylindrical, gradually tapering towards vasa deferentia. Vasa with irregular coils around ovaries in XII, about 17 µm wide. Penial bulbs round 230 µm in diameter. Several large oocytes but no mature eggs present.

Spermathecae (Fig. 18B) in V, club-shaped, with ampulla distinctly set apart from ectal duct. Ampulla sub-spherical, thin-walled, entally possibly communicating with oesophagus. Sperm in ampulla, aggregated into irregular mass or in circle embedded in wall of ampulla. Spermathecae 290–350 µm long, 180–190 µm wide at widest part of ampulla. Gland cells surrounding ectal pore, forming large compact mass, 235–285 µm in diameter at its widest part. Four midventral subneural glands in XIV–XVII, 225 µm, 205 µm, 145 µm and 115 µm long, respectively.

#### Geographical distribution.

Originally described from Germany.

#### Remarks.

We have mounted and re-examined the only remaining syntype of *L. helgolandicus* from the Zoological Museum in Hamburg and found the worm to correspond well to the descriptions of *L. helgolandicus* by Michaelsen (1927, 1934) and no doubt to represent the attached species name. However, we found some discrepancies with the published measurements of the sperm funnels (also discussed above). As the slide of the mounted specimen is of good quality and since this is the only remaining syntype of the species we designate ZMH V5786 as the lectotype of *L. helgolandicus*.

Based on morphology, *L. helgolandicus* is similar to *L. scandicus* and is probably closely related to this species. In the DNA-based phylogeny, *L. scandicus* is placed close to the *lineatus* group (Fig. 1), and it is likely that *L. helgolandicus* phylogenetically belongs there too. However, for convenience of morphological identification, both taxa are referred to the paraphyletic “*tuba*” group.

### The *buelowi* group

*Characteristics*: Testes with testis sacs irregularly lobed and compact. Spermathecae with long duct distinctly set off from ampulla, and glands surrounding the ectal pore. Chaetae usually 2–3 per bundle; upper bundles midlateral, just above the lateral line. Penial bulbs round. Sperm funnels about as long as wide.

### Lumbricillus buelowi

Taxon classificationAnimaliaEnchytraeidaEnchytraeidae

Nielsen & Christensen, 1959

Fig. 19

Lumbricillus buelowi Nielsen & Christensen, 1959: pp. 106, figs 121–124 & 129; Erséus et al. 2010; Klinth et al. 2017.
Fridericia bulbosa ; sensu von Bülow 1957: pp. 87–88, pl. XXVII, figs 5–11; nec Rosa, 1887.

#### Type material.

Typus amissus (Nomenclatura Oligochaetologica). Type locality not precisely defined; the species was originally described from four different sites (Kalø, Femmøller, Ebeltoft and Avedøre) in Denmark (Nielsen and Christensen 1959). We did not designate a neotype as we do not have material from any of the type localities.

#### Material examined.

SMNH 152719 (CE5224), one mature specimen from Sweden, and ZMBN 107802 (CE22293), ZMBN 107804 (CE23273), ZMBN 107805 (CE23375), ZMBN 107806 (CE23376), ZMBN 107811 (CE24678), ZMBN 107814 (CE24688) & ZMBN 107816 (CE24690), seven mature specimens from Norway. For information on specimen collection localities and GenBank accession numbers see Appendix 1.

#### Geographical distribution including BOLD data.

Genetically identified from Norway and Sweden. Also known from Denmark and Germany. BIN-number: BOLD:ACQ3084.

#### Description.

**Figure 19.**
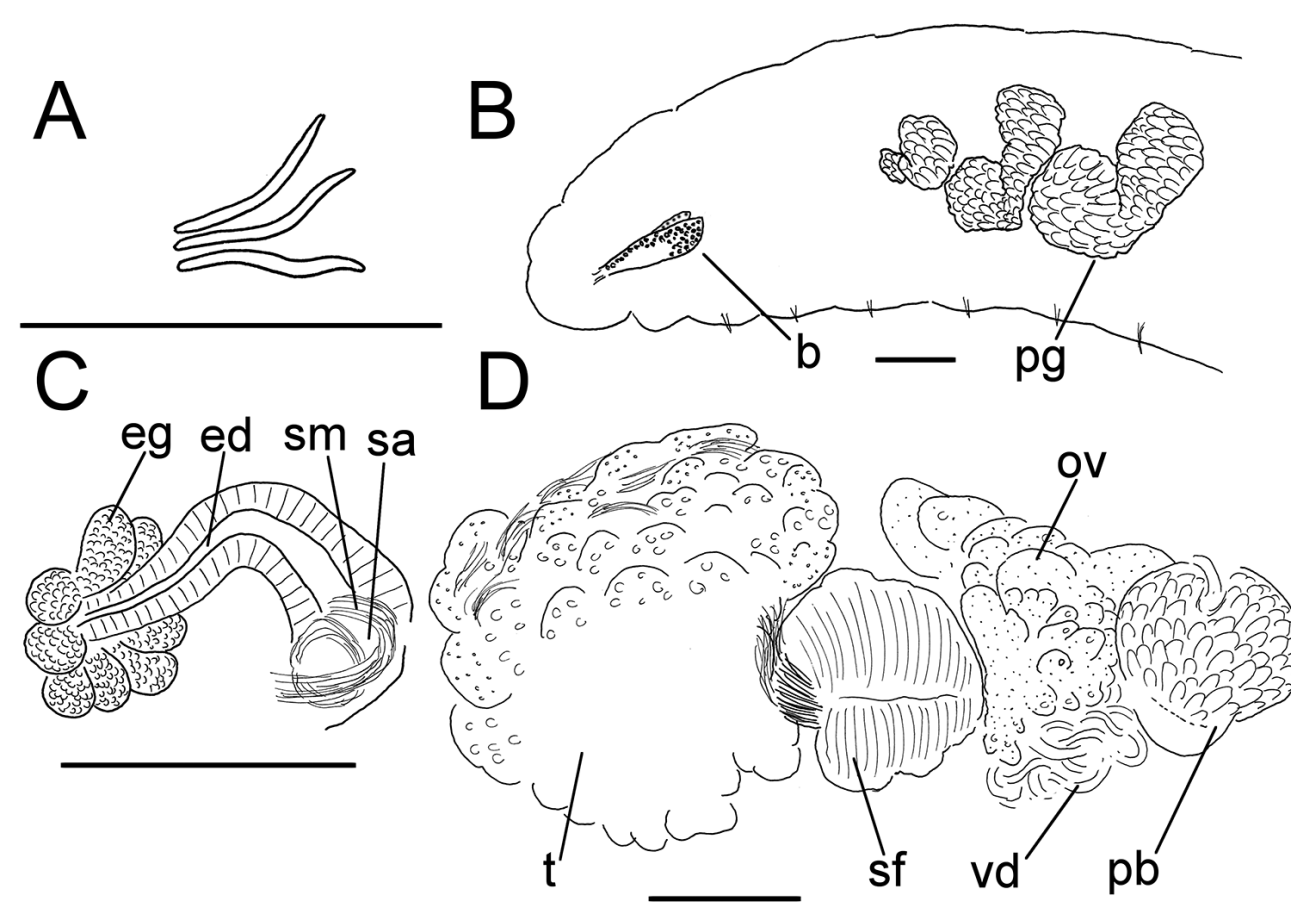
*Lumbricillus buelowi*. **A** Chaetal bundle **B** Anterior body **C** Spermatheca **D** Other genitalia. Abbreviations under general notes. Scale bars: 100 µm.

White to slightly pink or yellow worms. Length (fixed worms) more than 2.4–5.2 mm (amputated specimens), first 15 segments 1.7–2.4 mm long, width at clitellum 0.28–0.49 mm. More than 21–32 segments. Chaetae straight or slightly sigmoid (Fig. 19A). Lateral bundles with 2–3 chaetae anterior to clitellum, 2(3) chaetae in postclitellar segments. Ventral bundles with 2–3(4) chaetae anterior to clitellum, 2(3–4) chaetae posteriorly. Each worm’s longest measured chaetae 30–55 µm long, about 5 µm wide. Clitellum extending over XII–1/2XIII. Head pore at 0/1. Epidermis with transverse rows of gland cells.

Coelomocytes numerous, 10–25 µm long, spindle-shaped, oval, round, granulated with distinct nucleus. Paired pharyngeal glands present in IV, V and VI; each pair converging dorsally (Fig. 19B). Dorsal vessel originating in XIV. Nephridia observed in VIII–X and XV–XXVIII, about 95 µm long, anteseptale funnel only, postseptale oval, tapering into posterior efferent duct. Brain slightly longer than wide, with posterior incision.

Male genitalia paired (Fig. 19D). Testes originating in XI, extending forwards into X, with testis sacs covering compact mass, slightly but not regularly lobed. Sperm funnels in XI, 85–140 µm long, 100–145 µm wide, making them about as long as wide, funnels tapering towards vasa deferentia. Most of vasa irregularly coiled in XII, 5–10 µm wide. Penial bulbs round, 75–110 µm in diameter, everted in one specimen. Ovaries in XII. One to three mature eggs present at a time.

Spermathecae (Fig. 19C) in V, club-shaped, with distinct ampulla. Ectal duct narrow, more than twice the length of the ampulla, abruptly widening into ampulla. Ampulla round, entally connecting with oesophagus. Sperm in ampulla aggregated into central mass haloed by circle of spermatozoa. Spermathecae 130–160 µm long, 45–65 µm wide at widest part of ampulla. Gland cells surrounding ectal pore, folded, glandular body 40–90 µm in diameter at its widest part. Up to five midventral subneural glands in XIII– XVII, 50–95 µm, 60–120 µm, 60–95 µm, 50–65 µm and 45 µm long, respectively; glands in XVI–XVII not observed in all specimens.

#### Geographical distribution including BOLD data.

Genetically identified from Norway and Sweden. Also known from Denmark and Germany. BIN-number: BOLD:ACQ3084.

#### Remarks.

It is clear that the two species here identified as *L. buelowi* and *L. knoellneri* Nielsen & Christensen, 1959 are closely related (Klinth et al. 2017) (see also Fig. 1), and most morphological characters such as the spermathecae, sperm funnels and penial bulbs are virtually identical between them. However, there are some general differences in our studied material: *L. buelowi* is on average larger than *L. knoellneri*, as originally noted by Nielsen and Christensen, although there is overlap between the two. *Lumbricillus buelowi* possesses 2–3 chaetae in the lateral bundles anterior of the clitellum while *L. knoellneri* possesses only 2. In their original description, Nielsen and Christensen also differentiate the two species by colour and size of coelomocytes where *L. buelowi* is red with two types of coelomocytes, one being larger than the chaetae, compared with *L. knoellneri* which is white and have only one type of coelomocytes, shorter than the chaetae. We observed only the smaller coelomocytes in both species, and as we stained the material we only have observations of the live animals; some specimens of *L. buelowi* were noted as being pinkish.

There are a number of species with descriptions similar to the ones of *L. buelowi*, and therefore also of *L. knoellneri*, such as *L. eltoni* (Stephenson, 1924), *L. muscicolus* (Stephenson, 1924) and *L. nielseni* Nurminen, 1965. All these latter three were described from Svalbard where we found specimens of *L. knoellneri* but not *L. buelowi*. Unfortunately, the two species described by Stephenson (1924) were not illustrated and the descriptions are not extensive enough for us to synonymize either of them with *L. knoellneri* (Stephenson’s species would in that case hold seniority in name). Also *L. nielseni* was described too briefly (Nurminen 1965a) and the illustrated spermathecae seems slightly different from those of *L. buelowi* and *L. knoellneri*, causing us once again to avoid synonymization.

### Lumbricillus knoellneri

Taxon classificationAnimaliaEnchytraeidaEnchytraeidae

Nielsen & Christensen, 1959

Fig. 20

Lumbricillus knoellneri Nielsen & Christensen, 1959: pp. 106–107, figs 125–126, 130; Klinth et al. 2017.
Fridericia bulbosa ; sensu Knöllner 1935: p. 443; nec Rosa, 1887.

#### Type material.

Typus amissus (Nomenclatura Oligochaetologica). Type locality: Ebeltoft Vig, Denmark (Nielsen and Christensen 1959). We did not designate a neotype as we do not have material from the type locality.

#### Material examined.

SMNH 152734 (CE980) & SMNH 152735 (CE982), two mature specimens from Sweden, and ZMBN 107859 (CE19369), ZMBN 107860 (CE20761), ZMBN 107861 (CE20762), ZMBN 107863 (CE22615), ZMBN 107865 (CE23252) & ZMBN 107866 (CE23253), four mature and two immature specimens from mainland Norway and Svalbard. For information on specimen collection localities and GenBank accession numbers see Appendix 1.

#### Description.

**Figure 20.**
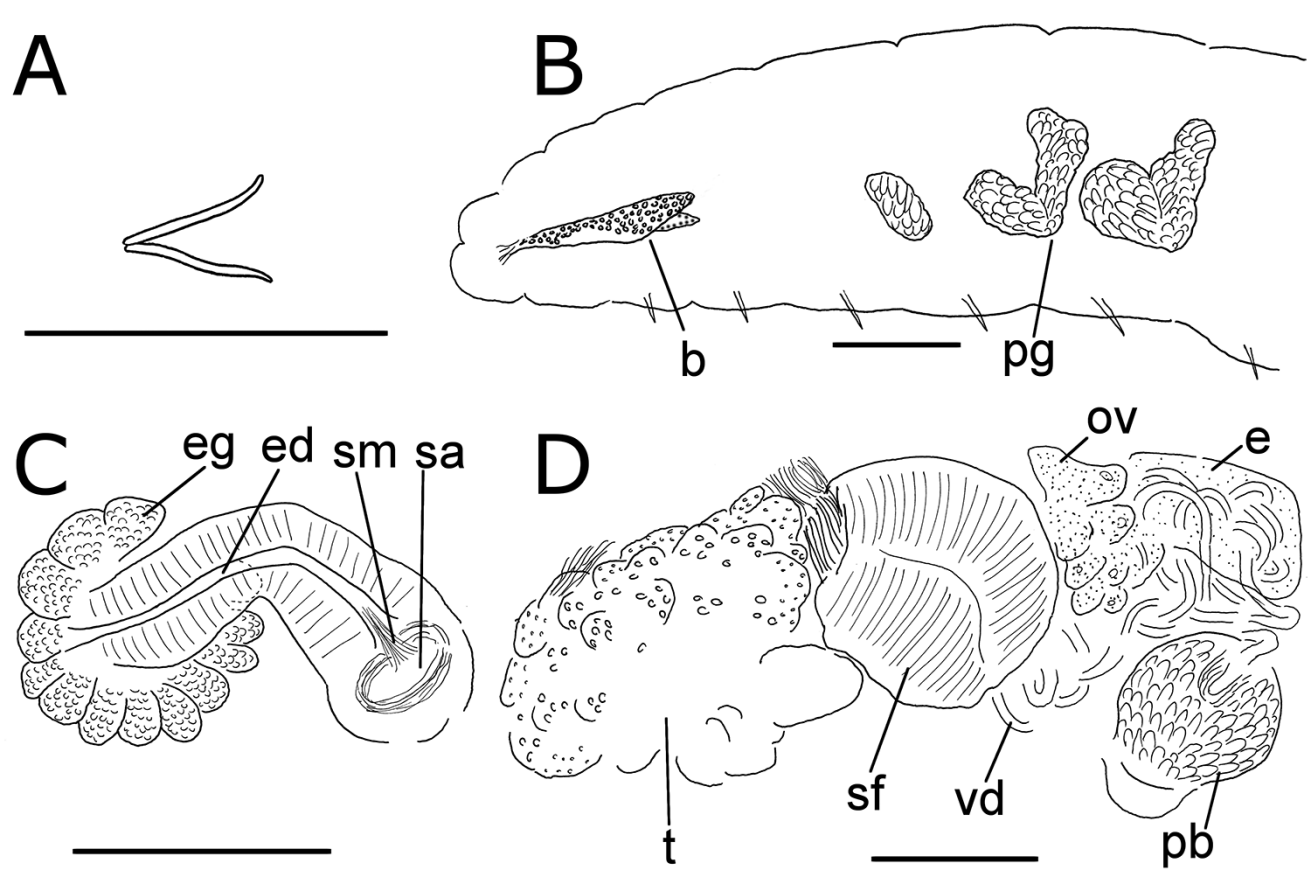
*Lumbricillus knoellneri*. **A** Chaetal bundle **B** Anterior body **C** Spermatheca **D** Other genitalia. Abbreviations under general notes. Scale bars: 100 µm.

White to yellow worms. Length (fixed worms) more than 2.1–3.6 mm (amputated specimens), first 15 segments 1.6–1.9 mm long, width at clitellum 0.20–0.32 mm. More than 16–32 segments. Chaetae straight or slightly sigmoid (Fig. 20A). Lateral bundles with 2 chaetae anterior to clitellum, 2 chaetae in postclitellar segments. Ventral bundles with 2–3 chaetae anterior to clitellum, 2 chaetae posteriorly. Each worm’s longest measured chaetae 25–50 µm long, about 3 µm wide. Clitellum extending over XII–1/2XIII. Head pore at 0/1. Epidermis with transverse rows of gland cells.

Coelomocytes numerous, 10–25 µm long, spindle-shaped, oval, round, granulated with distinct nucleus. Paired pharyngeal glands present in IV, V and VI (Fig. 20B). Dorsal vessel originating in XIII–XV. Nephridia observed in VIII–X and XV–XXXII, about 70 µm long, anteseptale funnel only, postseptale oval, tapering into posterior efferent duct. Brain with posterior incision.

Male genitalia paired (Fig. 20D). Testes originating in XI, extending forwards into X, with testis sacs covering compact mass, slightly lobed but not regularly arranged. Sperm funnels in XI, 100–150 µm long, 70–155 µm wide, making them about as long as wide or 1.5 times longer than wide, funnels tapering towards vasa deferentia. Most of vasa irregularly coiled in XII, 5–10 µm wide. Penial bulbs round, 70–115 µm in diameter. Ovaries in XII. One to two mature eggs present at a time.

Spermathecae (Fig. 20C) in V, club-shaped, with distinct ampulla. Ectal duct more than twice the length of the ampulla, abruptly widening into ampulla. Ampulla round, entally connecting with oesophagus. Sperm in ampulla aggregated into central mass haloed by circle of spermatozoa. Spermathecae 145–270 µm long, 45–65 µm wide at widest part of ampulla. Gland cells surrounding ectal pore, divided in several small lobes, whole glandular body 75–100 µm in diameter at its widest part. Up to four midventral subneural glands in XIII– XVI, 50–65 µm, 35–65 µm, 35–65 µm and 35–60 µm long, respectively; glands in XVI not observed in all specimens.

#### Geographical distribution including BOLD data.

Genetically identified from Norway (mainland and Svalbard) and Sweden. Also known from Denmark and Germany. BIN-number: BOLD:ACM5261.

#### Remarks.

*Lumbricillus knoellneri* is described as having only 2 chaetae throughout the body but the newly studied material suggests that the preclitellar ventral bundles possess 2–3 chaetae. In fact, all of the eight studied specimens had 3 chaetae in at least 2 of the preclitellar ventral bundles, some in as many as 9. We found no pattern of this distribution and for each preclitellar ventral segment bearing chaetae (II–XI), we found representatives with either 2 or 3 chaetae. This shows how variable this trait is and could explain the difference to the description by Nielsen and Christensen. However, it could also mean that our “*L. knoellneri*” is in fact another species. Many of the internal organs of *L. knoellneri* were as long as or even slightly longer than the ones in *L. buelowi*. This in combination with a generally smaller size caused the segments of *L. knoellneri* to appear more contracted. For a further discussion see the Remarks for *L. buelowi* above.

In 1985, Kossmagk-Stephan synonymized *L. cervisiae* (which he himself had described as a new species two years earlier) and *L. christenseni* Tynen, 1966 with *L. knoellneri*. All three species are small, have only two chaetae per bundle (at least according to the original descriptions) and similarly shaped spermathecae. However, *L. christenseni* has a sperm funnel that is 7–8 times longer than wide which is significantly longer than the 1.5 times measured in *L. knoellneri*. The sperm funnel of *L. cervisiae* is 3–4 times longer than wide which is also more than that of *L. knoellneri*. Furthermore, the testis sacs of *L. cervisiae* cover several small scattered lobes and the vasa deferentia extends backwards into XIII. Finally, *L. cervisiae* appears to be more slender than *L. knoellneri* and has significantly smaller internal organs, which we were able to discern by examining the mounted original material of Kossmagk-Stephan. Therefore, we reject the idea of *L. cervisiae* and *L. christenseni* being synonyms of *L. knoellneri* and treat them as separate species.

### The *arenarius* group

*Characteristics*: Testes with testis sacs irregularly lobed. Spermathecae with short gradually widening duct, which is difficult to distinguish from ampulla, and glands surrounding the ectal pore. Chaetae usually 2–3 or more per bundle; upper bundles midlateral, just above the lateral line. Penial bulbs round or bilobed. Sperm funnels three to ten times longer than wide.

### Lumbricillus arenarius

Taxon classificationAnimaliaEnchytraeidaEnchytraeidae

(Michaelsen, 1889)

Fig. 21

Enchytraeus arenarius Michaelsen, 1889: pp. 12–14, figs 5a–d.
Marionina arenaria ; Michaelsen 1900: pp. 74–75.
Enchytraeoides arenarius ; Ude 1929: pp. 62–63; von Bülow 1957: p. 84; Knöllner 1935: pp. 437–438, figs 7–8.
Lumbricillus arenarius ; Nielsen and Christensen 1959: pp. 107–108, figs 127–128; Rota and Healy 1999: pp. 53–54; Erséus et al. 1999; Erséus et al. 2010; Klinth et al. 2017.
Lumbricillus magdalenae Nurminen, 1965: pp. 6–7, figs 2e–g.

#### Type material.

Typus amissus (Nomenclatura Oligochaetologica). Type locality: Elbe River, Hamburg, Germany (Michaelsen 1889). We did not designate a neotype as we do not have material from the type locality.

#### Material examined.

SMNH 152716 (CE1001), one mature specimen from Sweden, and ZMBN 107784 (CE8474), ZMBN 107787 (CE20748), ZMBN 107788 (CE20749) & ZMBN 107789 (CE20750), four mature specimens from Norway. For information on specimen collection localities and GenBank accession numbers see Appendix 1.

#### Description.

**Figure 21.**
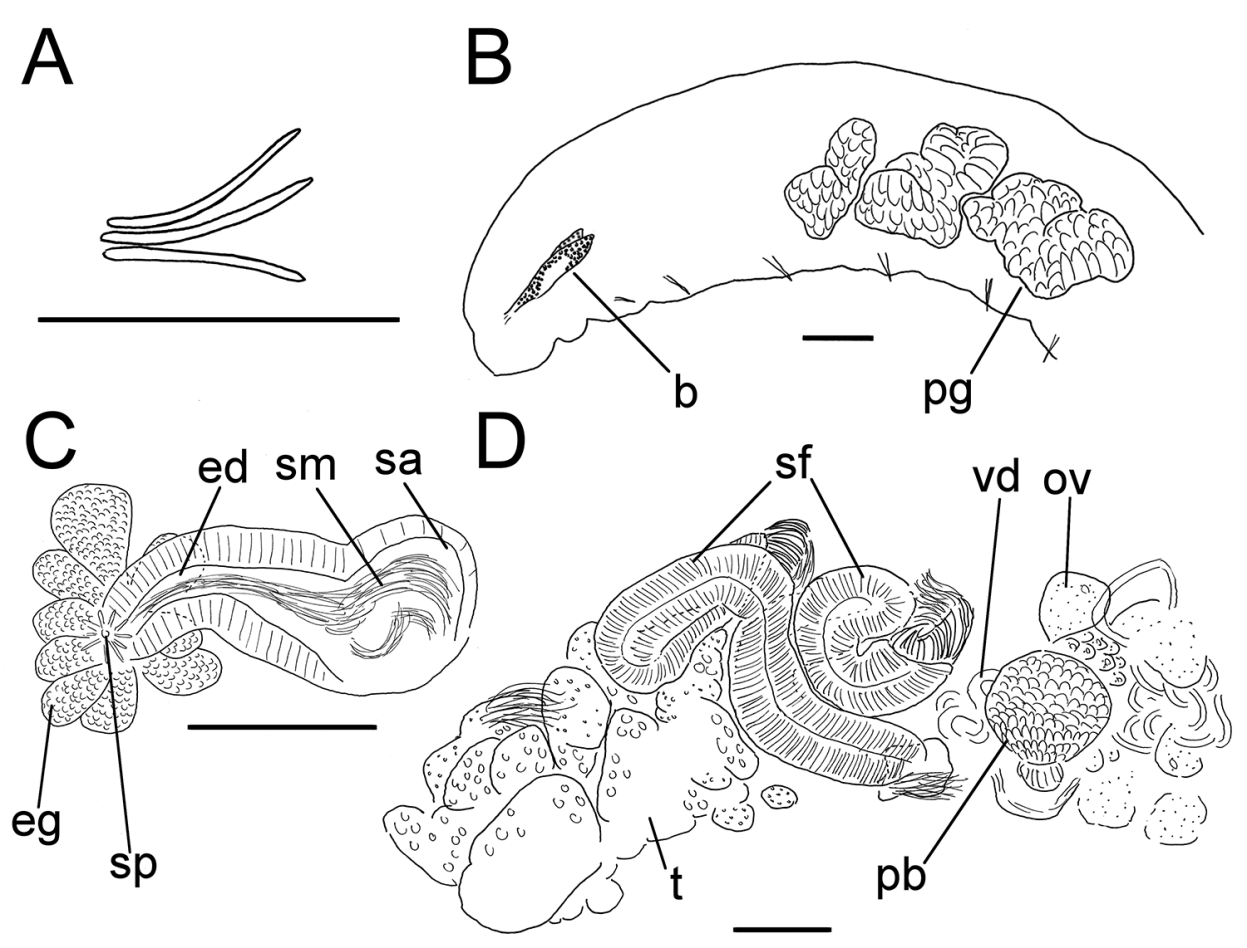
*Lumbricillus arenarius*. **A** Chaetal bundle **B** Anterior body **C** Spermatheca **D** Other genitalia. Abbreviations under general notes. Scale bars: 100 µm.

White to yellow worms. Length (fixed worms) more than 5.0–8.6 mm (amputated specimens), first 15 segments 3.5–4.0 mm long, width at clitellum 0.31–0.51 mm. More than 19–35 segments. Chaetae straight or slightly sigmoid (Fig. 21A). Lateral bundles with 2–3 chaetae anterior to clitellum, 2 chaetae in postclitellar segments. Ventral bundles with 2–3(4) chaetae anterior to clitellum, 2–3 chaetae posteriorly. Each worm’s longest measured chaetae 40–70 µm long, about 5 µm wide. Clitellum extending over XII–1/2XIII, in some covering all of XIII. Head pore at 0/1. Epidermis with transverse rows of gland cells.

Coelomocytes numerous, 20–50 µm long, spindle-shaped, oval, round, granulated with distinct nucleus, some with distally hooked ends. Paired pharyngeal glands present in IV, V and VI (Fig. 21B). Dorsal vessel originating in XIII. Nephridia observed in XV–XVI and XX–XXV, 105–145 µm long, anteseptale funnel only, postseptale oval, tapering into efferent duct. Brain with posterior incision.

Male genitalia paired (Fig. 21D). Testes originating in XI, extending forwards into X, with testis sacs covering mass of rather large irregularly arranged lobes. Sperm funnels in XI, in some specimens extending back into XII, 375–975 µm long, 55–103 µm wide, making them 6–13 times longer than wide, funnels tapering towards vasa deferentia. Most of vasa irregularly coiled in XII, in one specimen extending back into XIV, 5–10 µm wide. Penial bulbs round, 110–140 µm in diameter. Ovaries in XII. One to six mature eggs present at a time.

Spermathecae (Fig. 21C) in V, pouch-shaped. Ectal duct longer than and gradually widening into ampulla. Ampulla oval or round, entally connecting with oesophagus. Irregular mass of sperm aggregated in ampulla. Spermathecae 100–255 µm long, 50–115 µm wide at widest part of ampulla. Gland cells surrounding ectal pore, divided into several flaps, whole glandular body 75–135 µm in diameter at its widest part. Up to four midventral subneural glands in XIII–XVI, 75–110 µm, 90–115 µm, 75–85 µm and 95 µm long, respectively; glands in XVI not observed in all specimens.

#### Geographical distribution including BOLD data.

Genetically identified from Norway (mainland and Svalbard) and Sweden. Also reported from Denmark, Canada, Germany, Greenland, Iceland, Ireland, Wales and North–Western Australia (Rota and Healy 1999). BIN-number: BOLD:AAT8953.

#### Remarks.

The original description by Michaelsen (1889) was later amended by Knöllner (1935) who redrew the shape of the nephridia and spermathecae, also confirmed by Nielsen and Christensen (1959). The newly examined material in this study resembles the original description in most characters but the spermathecae and nephridia are in agreement with the amended descriptions. Coelomic corpuscles were found with hooked ends which seemed to bind to the internal tissue in a way that is described by Michaelsen. The testes seemed to be either an irregular compact mass or divided into separate lobes, encased in testis sacs, but these lobes were not arranged in the bunch-shape seen in the *lineatus*, *pagenstecheri* and “*tuba*” groups.

*Lumbricillus arenarius* is genetically closely related to *L.* sp. H and *L. dubius* (Fig. 1).

### Lumbricillus

Taxon classificationAnimaliaEnchytraeidaEnchytraeidae

sp. H

Fig. 22

Lumbricillus sp. H; Klinth et al. 2017.

#### Material examined.

ZMBN 107945 (CE23136), ZMBN 107947 (CE24967) & ZMBN 107948 (CE24968), three half mature specimens from Norway. For information on specimen collection localities and GenBank accession numbers see Appendix 1.

#### Description.

**Figure 22.**
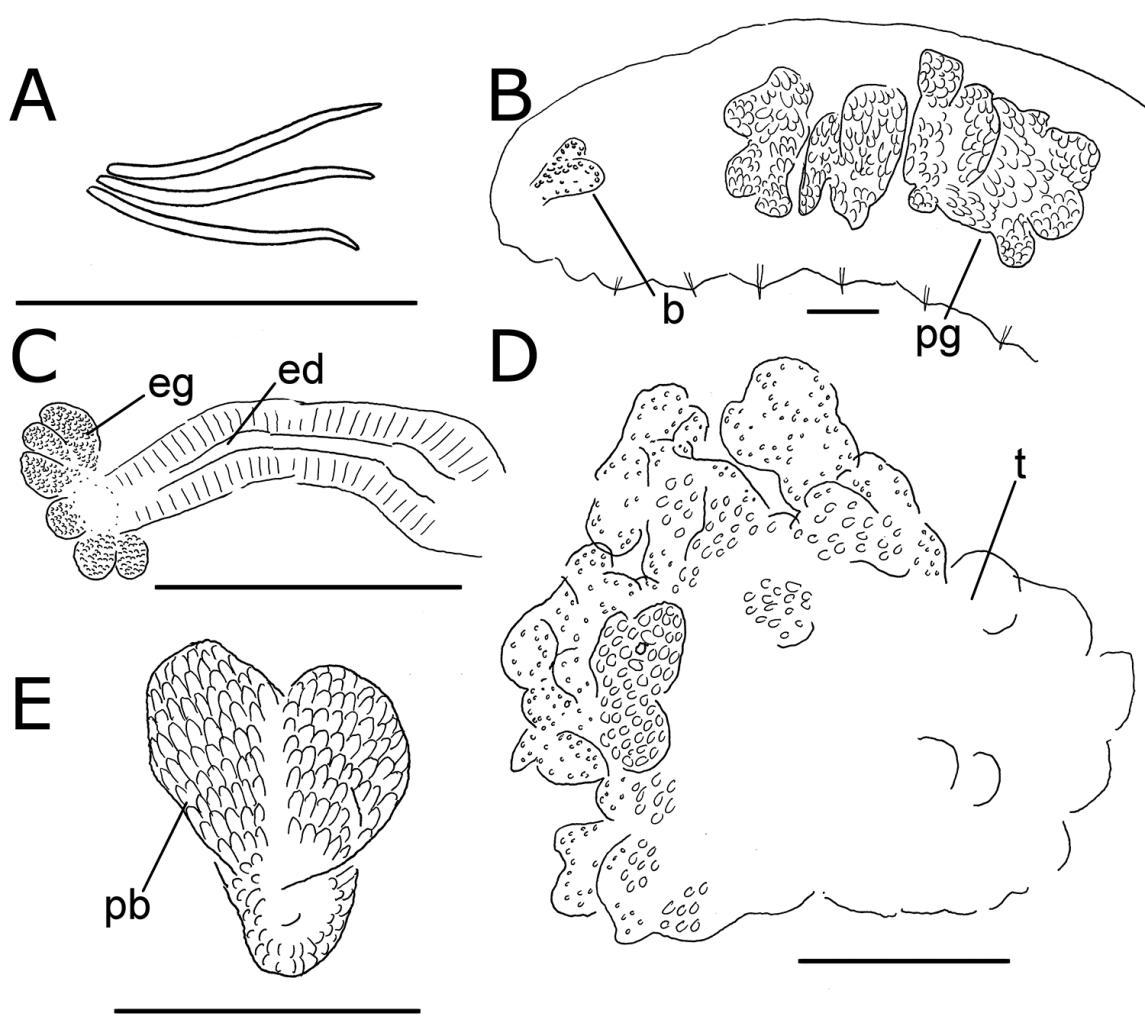
*Lumbricillus* sp. H. **A** Chaetal bundle **B** Anterior body **C** Spermatheca **D** Testis **E** Penial bulb. Abbreviations under general notes. Scale bars: 100 µm.

White to orange worms. Length (fixed worms) more than 3.8–5.4 mm (amputated specimens), first 15 segments 2.0–2.8 mm long, width at clitellum 0.40–0.42 mm. More than 31–33 segments. Chaetae straight or slightly sigmoid (Fig. 22A). Lateral bundles with 2–3 chaetae anterior to clitellum, 2 chaetae in postclitellar segments. Ventral bundles with 2–3 chaetae anterior to clitellum, 2 chaetae posteriorly. Each worm’s longest measured chaetae 70–75 µm long, about 5 µm wide. Clitellum extending over XII–1/2XIII. Head pore at 0/1. Epidermis with transverse rows of gland cells.

Coelomocytes numerous, 15–20 µm long, spindle-shaped, oval, round, granulated with distinct nucleus. Paired pharyngeal glands present in IV, V and VI, sometimes extending into VII; each pair converging dorsally (Fig. 22B). Dorsal vessel originating in XIII. Nephridia observed in XIII–XXVIII, 100–145 µm long, anteseptale funnel only, postseptale oval, tapering into efferent duct. Brain with posterior incision.

Male genitalia paired. Testes (Fig. 22D) originating in XI, extending forwards into X, in one specimen back into XII, with testis sacs covering mass of irregularly arranged lobes. Sperm funnels in XI, 145–170 µm long, 45–50 µm wide, making them 3–4 times longer than wide, funnels tapering towards vasa deferentia. Most of vasa irregularly coiled in XII, 10 µm wide. Penial bulbs (Fig. 22E) slightly bilobed, 85–120 µm in diameter. Ovaries in XII. Mature eggs not observed.

Spermathecae (Fig. 22C) in V, pouch-shaped. Ectal duct long, gradually widening. Ampulla not clearly set off from duct, entally connecting with oesophagus. No sperm observed. Spermathecae 125–145 µm long, 25–40 µm wide at widest part. Gland cells surrounding ectal pore, divided into several lobes, whole glandular body 35–65 µm in diameter at its widest part. Two midventral subneural glands in XV– XVI, 45–100 µm, 50–65 µm long, respectively.

#### Geographical distribution.

Genetically identified from Norway.

#### Remarks.

Initial comparisons found similarities between this species and *Lumbricillus westheidei* Kossmagk-Stephan, 1983, such as similar shape of spermathecae and slightly bilobed penial bulbs. However, having re-examined Kossmagk-Stephan’s type material we found some important differences compared to our specimens. First, *L. westheidei* has only two chaetae per bundle, whereas our specimens have up to three chaetae in the preclitellar segments (the position of the upper bundles is identical in the two species). Second, the three pairs of pharyngeal glands are clearly separated in *L. westheidei* but in our specimens at least the first two pairs appear to have a dorsal connection. Third, the testis sacs of *L. westheidei* are much smaller than the ones we observed in our specimens. Fourth, the vasa deferentia appear to be much longer and form many more coils in segment XII in *L. westheidei* compared to our *L.* sp. H. Finally, the sperm funnels of *L. westheidei* are about 10 times longer than wide, against the 4:1 length:width ratio observed in our specimens. Unfortunately, none of our examined specimens appeared to be fully mature, as sperm were not observed either at the sperm funnels or in the spermathecae, and there were no mature eggs present. This suggests that the sperm funnels and spermathecae were not fully developed and could at maturity resemble those of *L. westheidei* more. Due to this uncertainty we cannot completely rule out that our specimens are of the same species as *L. westheidei*; however, for now we will continue to treat it as an unknown species, simply referred to as *L.* sp. H.

Since *L. westheidei* resembles, in its general morphology, our *L.* sp. H, it is important to add some notes on its generic allocation. Kossmagk-Stephan (1983) questioned the placement in *Lumbricillus* due to the undivided testis sacs. He had also observed this feature in some other “*Lumbricillus*” species, such as *L. arenarius* and *L. semifuscus*, the latter here below transferred to *Claparedrilus* gen. n. Furthermore, he noted a similarity in the morphology of the spermathecae between *L. westheidei*, *L. buelowi*, *L. knoellneri* and *L. codensis* Lasserre, 1971. In 1985, Coates and Erséus established the new genus *Randidrilus* and designated *Lumbricillus codensis* as its type species. Because of the resemblance to the latter in the bilobed penial bulb, the long spermathecal ectal duct, the long sperm funnels and the undivided testis sac, Kossmagk-Stephan (1985) proposed in his doctoral thesis the new combination, *R. westheidei*, and since then the species was regarded as another member of *Randidrilus* (Coates, 1989; Mackei and Erséus 1997; Schmelz and Collado 2012). However, we confirm here that, unlike other species of *Randidrilus*, *L. westheidei* has more than a single chaeta per bundle, does not lack chaetae in numerous lateral and ventral bundles and does not have an unpaired sperm sac extending backwards into postclitellar segments. Instead, *L. westheidei* resembles members of the *arenarius* group within *Lumbricillus* by having few chaetae, long sperm funnels, slightly bilobed penial bulbs and paired testis sacs that are not regularly lobed. Therefore, we transfer this species back from *Randidrilus* into *Lumbricillus*, making it *L. westheidei* once again.

*Lumbricillus* sp. H is genetically closely related to *L. arenarius* and *L. dubius* (Stephenson, 1911) (Fig. 1).

### Lumbricillus dubius

Taxon classificationAnimaliaEnchytraeidaEnchytraeidae

(Stephenson, 1911)

Fig. 23

Enchytraeus dubius Stephenson, 1911: pp. 54–58, figs 10–12 & pl. II, figs 12–14;
Lumbricillus dubius ; Nielsen and Christensen 1959: p. 96; Finogenova and Timm 1988: pp. 92–93, fig. 1; Klinth et al. 2017.

#### Type material.

Typus amissus (Nomenclatura Oligochaetologica). Type locality: Firth of Clyde, Wemyss Bay, United Kingdom (Stephenson 1911). We did not designate a neotype as we do not have material from the type locality.

#### Material examined.

SMNH 152726 (CE5221) & SMNH 152727 (CE5223), two mature specimens from Sweden, and ZMBN 107835 (CE22767), ZMBN 107836 (CE23370), ZMBN 107837 (CE23371), ZMBN 107839 (CE24700), ZMBN 107840 (CE24711) & ZMBN 107841 (CE24726), six mature specimens from Norway. For information on specimen collection localities and GenBank accession numbers see Appendix 1.

#### Description.

**Figure 23.**
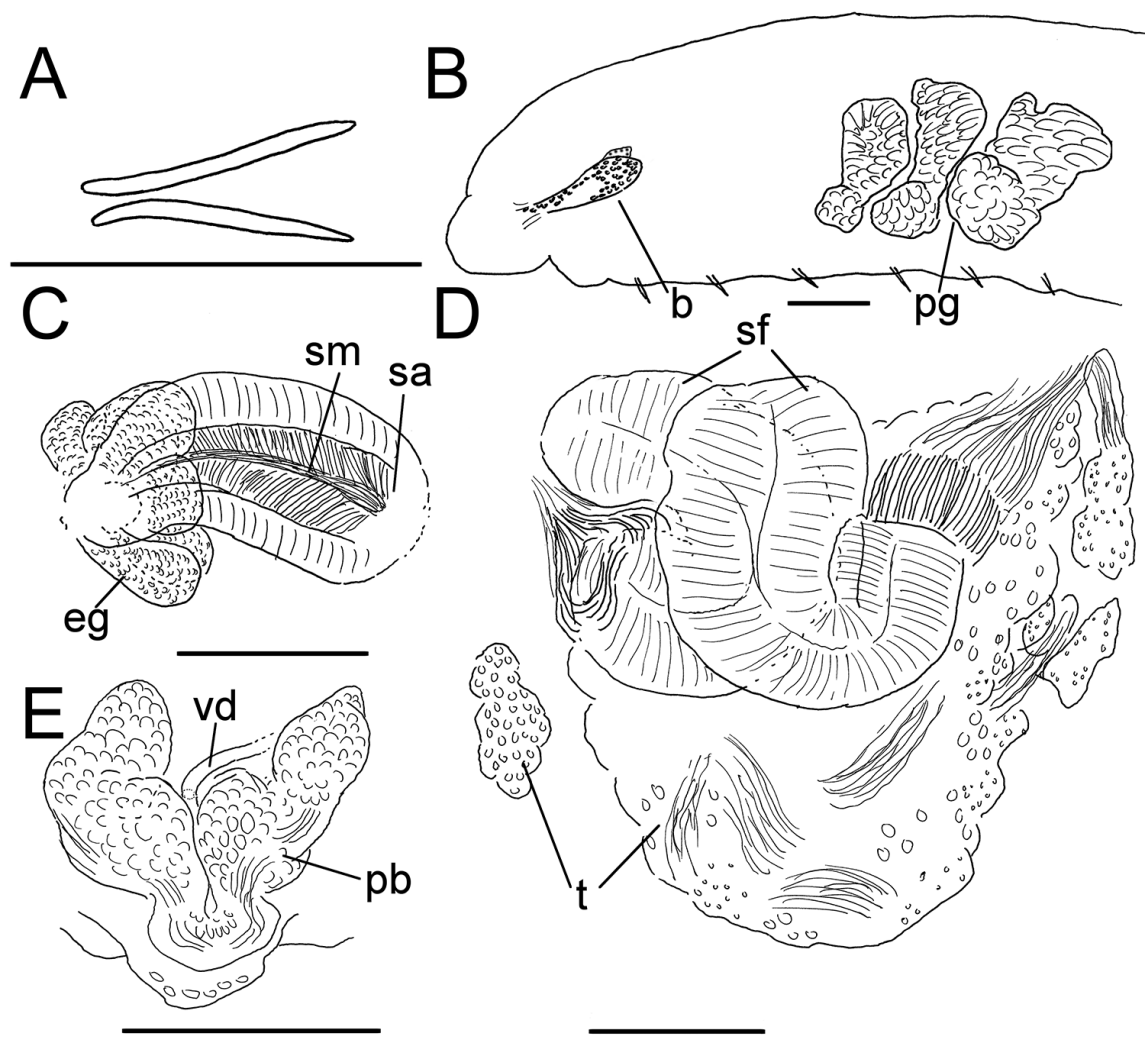
*Lumbricillus dubius*. **A** Chaetal bundle **B** Anterior body **C** Spermatheca **D** Male genitalia **E** Penial bulb. Abbreviations under general notes. Scale bars: 100 µm.

White to yellow worms. Length (fixed worms) more than 2.1–6.1 mm (amputated specimens), first 15 segments 1.5–2.5 mm long, width at clitellum 0.32–0.55 mm. More than 20–44 segments. Chaetae straight or slightly sigmoid (Fig. 23A). All observed bundles with two chaetae. Each worm’s longest measured chaetae 50–75 µm long, about 5 µm wide. Clitellum extending over XII–1/2XIII. Head pore at 0/1. Epidermis with transverse rows of gland cells.

Coelomocytes numerous, 15–30 µm long, spindle-shaped, oval, round, granulated with distinct nucleus. Paired pharyngeal glands present in IV, V and VI. Each pair converges dorsally, connection, if present at all, indistinct (Fig. 23B). Dorsal vessel originating in XIII. Nephridia observed in XVIII–XXI, 50–65 µm long, anteseptale funnel only, postseptale oval, tapering into efferent duct. Brain longer than wide, with posterior incision.

Male genitalia paired. Testes (Fig. 23D) originating in XI, in some specimens extending forwards into X, with testis sacs covering mass of irregularly arranged lobes and detached fragments, fragments spreading in XI–XII. Sperm funnels (Fig. 23D) in XI, 180–390 µm long, 70–145 µm wide, making them 2.5–4 times longer than wide, funnels tapering towards vasa deferentia. Most of vasa irregularly coiled in XI–XII, 5–10 µm wide. Penial bulbs (Fig. 23E), 110–190 µm in diameter, divided into two bulbs each with an extending horn. Ovaries in XII. One to five mature eggs present at a time.

Spermathecae (Fig. 23C) in V, pouch-shaped, without distinct ampulla, gradually widening, entally connecting with oesophagus. Sperm completely occupying lumen of duct and ampulla, regularly arranged with spermatozoan heads facing the wall and tails along the duct, forming denser aggregation throughout the centre of the spermathecae. Spermathecae 95–205 µm long, 40–100 µm wide at widest part of ampulla. Gland cells surrounding ectal pore, divided into few flaps, whole glandular body 70–120 µm in diameter at its widest part. Up to two midventral subneural glands in XIV– XV, 60–80 µm, 60–65 µm long, respectively; glands in XV not observed in all specimens.

#### Geographical distribution including BOLD data.

Genetically identified from Norway, Russia (White Sea), Sweden and the United Kingdom (Klinth et al. 2017). BIN-number: BOLD:AAU0151.

#### Remarks.

The specimens examined match the description of *Lumbricillus dubius* by Stephenson (1911) well, but they are smaller in body size and have testes that seem to form an irregular mass rather than being made up of branches as observed by Stephenson. The clearly divided penial bulbs and the morphology of the spermathecae bear a close resemblance between our specimens and the original description. Stephenson wrote that no sperm were observed in the spermathecae, but his illustrations depicting sections of the same clearly show the unique distribution of spermatozoa with heads regularly arranged perpendicular to the spermathecal wall. It is possible that he did not recognize them as sperm, simply because of this unusual arrangement.

*Lumbricillus dubius* has irregularly lobed testis sacs and spermathecae that are at least superficially similar to those of *L. arenarius*. The chaetae are straight to slightly sigmoid and few in number, which further supports the close relationship with *L. arenarius* and *L.* sp. H.

*Lumbricillus dubius* is genetically closely related to *L.* sp. H and *L. arenarius* (Fig. 1).

### Claparedrilus gen. n.

Taxon classificationAnimaliaEnchytraeidaEnchytraeidae

http://zoobank.org/E7A1215B-D41F-4721-89E9-226176994F0C

#### Genus description/diagnosis.

Prostomium hemispherical. Head pore at 0/1. Epidermis with transverse rows of gland cells. Chaetae straight to sigmoid, without nodulus, grouped into two dorsolateral and two ventrolateral bundles per segment. Oesophageal appendages absent. Pharyngeal glands in four pairs, in IV–VII, converging but not connected dorsally, some with ventral lobes, but without secondary glands. Only nucleated coelomocytes present. Dorsal vessel originating intra or in segments posterior to clitellum. Nephridia with anteseptale made up of funnel on a short stalk. Clitellum more or less covering segments XII–XIII. Testes paired, surrounded by testis sacs; the latter forming compact mass with shallow lobes irregularly arranged. Penial bulbs round and compact. Midventral subneural glands present in XIV–XV. Spermathecae in V, attached to and usually communicating with oesophagus lumen, and with crown of glands surrounding ectal part of ectal duct. Spermathecae club-shaped with ampulla distinctly set off from duct. Spermathecal diverticula absent. Marine, living in the littoral zone.

#### Type species.

*Claparedrilus semifuscoides* sp. n.

#### Other species.

*Claparedrilus semifuscus* (Claparède, 1961) comb. nov.

#### Etymology.

*Clapare*- from Claparède, the original author of the species *C. semifuscus*, a poorly defined species with which the type species for this new genus (*C. semifuscoides*) has been misidentified, and -*drilus* (latinized Greek) for worm.

#### Remarks.

The need for this new genus arose from the difficulty of placing the type species *C. semifuscoides* (which we previously referred to as *L. semifuscus*) in the phylogeny of the Enchytraeidae. Molecular data had previously supported that this species was not a member of *Lumbricillus* and instead closer to, but not a member of, *Globulidrilus* and *Bryodrilus* (Klinth et al. 2017; Martinsson et al. 2017). Both these genera share some traits with *Claparedrilus*, such as the shape of the spermathecae and nephridial anteseptale with small part of the nephridial body, but both have only three pairs of pharyngeal glands and are aquatic or terrestrial. The phylogenetic studies lacked representatives from several potential candidate genera of marine enchytraeids. Therefore, we compared the morphology of our species with these candidates (presented in Table 3), after which we still found support for the recognition of the new genus *Claparedrilus*. In particular, the combination of four pairs of pharyngeal glands, nephridia with a stalked funnel in the anteseptale and the presence subneural glands distinguishes this new genus.

**Table 3.** A comparison of characters distinguishing *Claparedrilus* gen. n. from other marine enchytraeid taxa. Traits of particular importance highlighted in boldface. *Coded according to Rota et al (2008); see differing interpretation in Schmelz and Collado (2008). ** One species (*Randidrilus quadrithecatus* Coates & Erséus, 1985) with two pairs of spermathecae, and four pairs of pharyngeal glands distributed from IV–VII.

Genus	Chaetal shape Upper bundles	Brain posterior	Pharyngeal glands	Coelomocytes	Gut appendages	Nephridial anteseptal	Blood vessel End/Origin	Testes	Subneural glands	Penial bulb	Spermathecal ampulla
*Claparedrilus*	Slightly sigmoid dorsolateral	Indented	Dorsally free; With ventral lobes; **4 pairs**	Nucleated	No.	**Funnel on a short thin stalk**	Peristomial/ XIII	Compact With seminal vesicles	**Yes**	Compact	No diverticula
*Christensenidrilus blocki* (Dózsa-Farkas & Convey, 1997)	Sigmoid dorsolateral	Slightly indented	Dorsally free; With secondary lobes; 3 pairs	**Anucleate**	No	**Funnel and some coils**	?/ XIII	Compact With seminal vesicles	No	Compact	No diverticula
*Lumbricillus* Ørsted, 1844	Straight to sigmoid **Dorso- or Midlateral**	Indented	Dorsally free or fused; With ventral lobes; 3 pairs	Nucleated	No	Funnel only	Peristomial/ XIII-XV	**Reg. or irreg. lobed With testis sacs**	**Yes or No**	Compact, rarely bilobed	No diverticula
*Marionina georgiana* (Michaelsen, 1888)	Sigmoid dorsolateral	Indented	Dorsally free; **No ventral lobes**; 3 pairs	Nucleated	No	Funnel only	Peristomial/ XIII	Compact With seminal vesicles	No*	Small*	No diverticula
*Randidrilus* Coates & Erséus, 1985	Slightly curved **Absent**	Deeply indented	Dorsally fused With ventral lobes; 3–4 pairs**	Nucleated	No	Funnel only	Peristomial/ **XX-XXIII**	Compact With sperm sacs	No	Bilobed	No diverticula
*Stephensoniella* Černosvitov, 1934	Str. or slightly sigmoid **Midlateral**	Slightly indented	Dorsally fused With ventral lobes; 3 pairs	Nucleated	No	Funnel only	Peristomial/ **XII-XXIII**	Compact With seminal vesicles	No	Compact	**Diverticulate**

### Claparedrilus semifuscoides sp. n.

Taxon classificationAnimaliaEnchytraeidaEnchytraeidae

http://zoobank.org/09A6ACEC-1D21-49AA-A134-6966F14D17C8

Fig. 24

Marionina semifusca ; sensu Stephenson 1911: pp. 35–39, figs 2–3, pl. I, fig. 2.
Lumbricillus semifuscus ; sensu Nielsen and Christensen 1959: p. 96; Erséus 1976: pp. 8–9, figs 5–6; Finogenova and Timm 1988: pp. 94–96, figs 2–4; Klinth et al. 2017; Martinsson et al. 2017.
? Marionina semifusca; sensu Southern 1907: p. 71; Southern 1909: pp. 148–149, pl. X, figs 9a–c.
? Lumbricillus semifuscus; Nurminen 1965a: p. 6.
Non Pachydrilus semifuscus Claparède, 1861: pp. 76–79, pl. II, figs 1–5.
Non Marionia semifusca; Michaelsen 1889: p. 29.
Non Marionina semifusca; Michaelsen 1900: p. 76.
Non Enchytraeoides semifuscus; Michaelsen 1927: p. 13, fig. 12; von Bülow 1957: p. 86.

#### Holotype.

SMNH Type-8932 [former SMNH 152823] (CE2249), a whole-mounted voucher of a sexually mature and DNA-barcoded worm (COI barcode is KU893995 in NCBI/GenBank; Klinth et al. 2017).

#### Type locality.

United Kingdom, Wales, Anglesey, Beaumaris, intertidal zone of beach with sand and algae, 53.2623 N, 4.0914 W, collected 15 Febuary 2007 by M. Strand and P. Sundberg.

#### Paratypes.

SMNH Type-8933 [former SMNH 152821] (CE2247), SMNH Type-8934 [former SMNH 152822] (CE2248), SMNH Type-8935 [former SMNH 152825] (CE2252), all whole-mounted sexually mature specimen from the type locality.

#### Other material examined.

ZMBN 107908 (CE23750) & ZMBN 107912 (CE24657), one mature and one half mature specimen from Norway. For information on specimen collection localities and GenBank accession numbers see Appendix 1.

#### Etymology.

Named after its similarity to *Claparedrilus semifuscus*, which it has previously been confused with and misidentified as.

#### Diagnosis.

This species can be distinguished from *C. semifuscus* by the size of the penial bulbs. In *C. semifuscus*, the bulbs are much larger than the sperm funnels, whereas in *C. semifuscoides* they are of about the same size as the funnels or smaller.

#### Description.

**Figure 24.**
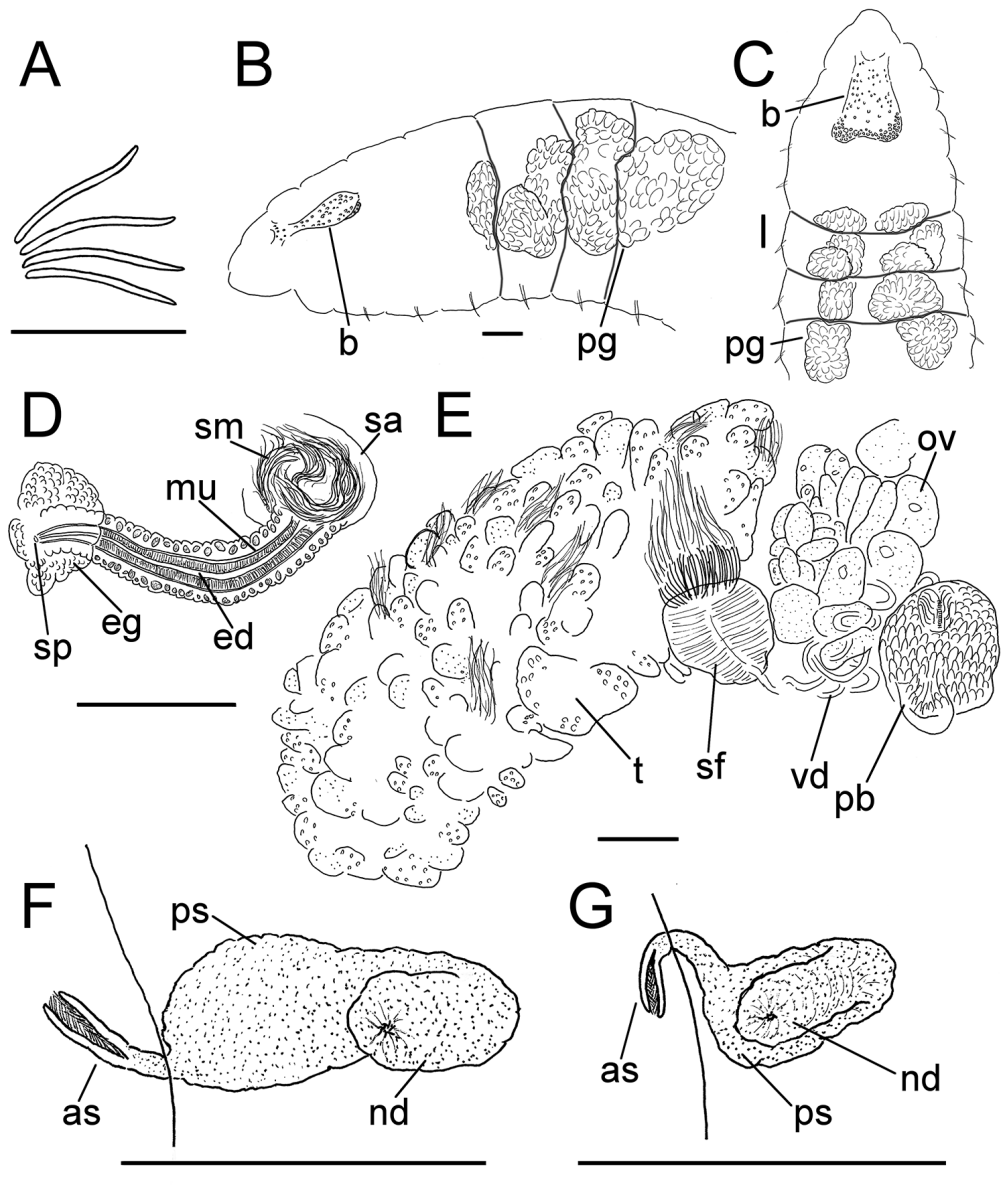
*Claparedrilus semifuscoides* sp. n. **A** Chaetal bundle **B** Anterior body **C** Anterior body dorsal view **D** Spermatheca **E** Other genitalia. **F.** Nephridium from XIV **G** Nephridium from XXIX in another individual. Abbreviations under general notes. Scale bars: 100 µm.

White, grey to pinkish worms. Length (fixed worms) more than 4.0–7.3 mm (amputated specimens), first 15 segments 2.1–3.4 mm long, width at clitellum 0.54–0.69 mm. More than 22–45 segments. Chaetae sigmoid or straight (Fig. 24A). Dorsal bundles with 2–5(6) chaetae anterior to clitellum, 2–5(6) chaetae in postclitellar segments. Ventral bundles with 3–6 chaetae anterior to clitellum, 2–5 chaetae posteriorly. Each worm’s longest measured chaetae 85–115 µm long, about 5–8 µm wide. Clitellum extending over XII–1/2XIII, sometimes XIII. Head pore at 0/1. Epidermis with transverse rows of gland cells.

Coelomocytes numerous, 10–20 µm long, spindle-shaped, oval, round, granulated with distinct nucleus. Paired pharyngeal glands 4 pairs, in IV, V, VI and VII, respectively; each pair converging but not connected dorsally (Figs 24B–C), pair in IV with dorsal lobes only, pair in V with both dorsal and ventral lobes, pairs in VI and VII large and compact, but dorsal lobes difficult to distinguish from potential ventral ones. Dorsal vessel originating in XIII. Nephridia (Figs 24F–G) observed in VI–VIII and XIV and onwards, 65–130 µm long, with various shapes, anteseptale with funnel on a thin stalk, postseptale oval, tapering into efferent duct which seems to originate either terminally or from posterior of the midventral of the postseptale (compare Figs 24F–G). Brain with posterior incision.

Male genitalia paired (Fig. 24E). Testes originating in XI, with testis sacs enclosing compact sperm mass with numerous inconspicuous, irregularly arranged lobes, extending forwards into X, in some specimens extending into IX and XII. Sperm funnels in XI, 125–160 µm long, 110–145 µm wide, making them 1–1.5 times longer than wide, funnels tapering towards vasa deferentia. Most of vasa irregularly coiled in XII, 10–15 µm wide. Penial bulbs round, 140–155 µm in diameter. Ovaries in XII. Two to five mature eggs present at a time.

Spermathecae (Fig. 24D) in V, club-shaped, with distinct ampulla. Ectal pore midlateral. Ectal duct seemingly divided into two zones by intermediary layer of musculature. The outer zone, or coelomic lining, covering muscular layer, containing large, clearly defined nuclei. The inner zone, which is the epithelium and cuticle, lining muscular layer, appearing to be made up by numerous fine lines (perpendicular to the duct axis); these lines possibly epithelial cells or their nuclei, or microvilli crossing the cuticle. Duct twice as long as ampulla, abruptly widening into ampulla. Ampulla round, entally connecting with oesophagus, and containing irregular mass of sperm in postcopulatory specimens. Spermathecae 240–270 µm long, 60–110 µm wide at widest part (the ampullae). Gland cells surrounding ectal duct near spermathecal pore, forming compact mass, 50–105 µm in diameter at its widest part. Two midventral subneural glands in XIV–XV, 40–100 µm, 70–85 µm long, respectively.

#### Geographical distribution.

Genetically identified from Norway and the United Kingdom. Also known (by morphology) from Iceland (Erséus 1976) and Sweden (Erséus 1977).

#### Remarks.

In 1861, Claparède described *Pachydrilus semifuscus* from the Hebrides in Scotland. Due to its unusual and confusing morphology, this species has been moved around among some enchytraeid genera. It was transferred to *Marionina* (Michaelsen 1900), then to *Enchytraeoides* (Michaelsen 1927), and finally to *Lumbricillus* (Nielsen and Christensen 1959). The original description focused almost entirely on the reproductive organs and noted sperm funnels about 1.5 times longer than wide, spermathecae with a long thin duct and clearly separated ampulla, nephridia with anteseptales made up of funnels only, and large kidney-shaped penial bulbs. Southern (1909), studying material from Dublin Bay (Ireland) and Edinburgh (Scotland), added that his specimens had 4–5 chaetae per bundle, a concave posterior of the brain, five pairs of pharyngeal glands in IV–VII (two of them in V), but with cylindrical rather than kidney shaped penial bulbs. Stephenson (1911) also recorded this species from Scotland but then increased the number of chaetae to 4–8 per bundle. He agreed with Southern’s description of the pharyngeal glands but questioned the shape of the penial bulbs, which he found to be spherical and not unusually large, compared to the descriptions by Claparède and Southern. In 1976, Erséus described the species from Iceland, also with pharyngeal glands in IV–VII, but with fewer chaetae per bundle and with anteseptales of the nephridia made up of a few coils as well as the funnels. Having studied our material, which is partly from Wales, we are confident that we have the same species as the one studied by Stephenson, Erséus, and possibly Southern, but that this (new) species is different from the original *Pachydrilus semifuscus*. Considering the way that it has been misidentified throughout history, we have named it *semifuscoides* and, at the same time, established a new genus for it called *Claparedrilus*. However, based on the similarities in the spermathecae and nephridia, we have decided to also transfer *L. semifuscus* into this genus, making it *C. semifuscus* (Claparède, 1861) comb. nov.

*Claparedrilus semifuscoides* can be separated from *C. semifuscus* by the size of the penial bulbs, where the former species have bulbs about the same size as the sperm funnels (about 150 µm in diameter) whereas the latter have bulbs larger than the funnels; they are 400–500 µm long. The nephridium illustrated by Claparède is reminiscent of what we observed (Fig. 24F–G), although our specimens seem to have the septa further back in relation to the funnel, making the funnel appear with a thin stalk, and with the efferent duct originating much further back on the postseptale. Unfortunately, Claparède did not mention the number of chaetae, subneural glands, or pharyngeal glands for his species, which makes its placement into the new genus *Claparedrilus* a bit tentative. This, and the fact that we have no genetic information for *C. semifuscus*, are the reasons why we designated the new taxon, *C. semifuscoides*, as the type species of the new genus.

Our specimens of *C. semifuscoides* are smaller than the ones described by Stephenson as *M. semifusca*, and they possess fewer chaetae, but we still believe that they belong to the same species. Stephenson remarks that (1) the nephridia can be found from V, (2) the anteseptale is made up of funnel only, and (3) the efferent duct extends backwards towards the pore, not forwards as illustrated by Claparède for *C. semifuscus*. We found nephridia from VI (possibly not finding any in V because they were obscured by the pharyngeal glands) and observed that the anteseptale consists of a funnel on a thin stalk. As this character was difficult to see and because there is no true nephridial tissue anterior to the septa this could still have been interpreted by Stephenson as a funnel only. We found that the efferent duct extended forward towards the pore which is more in agreement with Claparède’s illustration of *C. semifuscus* than what Stephenson noted, but the interpretation of this character may differ as the animal extends or contracts. Finally, Stephenson stated that the efferent duct originates well in front of the middle of the postseptale, whereas we observed it originating behind the middle or even from the posterior end. However, Stephenson also noted that this was not apparent in living specimens and only became clear from sections, which we have not studied.

Our specimens of *C. semifuscoides* also largely agree with Southern’s account of *M. semifusca* except for his description of the cylindrical penial bulbs. It is possible that the bulbs he studied were everted (as illustrated for *L. pagenstecheri* A in the present study; Fig. 14), which may have given the impression of them being cylindrical rather than spherical. Unfortunately, Southern did not mention the exact size of the bulbs or their size in relation to that of the sperm funnels, which makes us unable to confidently conclude that his species is the same as ours.

The species reported as *L. semifuscus* from Iceland (Erséus 1976) is probably the same as our *C. semifuscoides*, as Erséus noted the four pairs of pharyngeal glands and an anteseptale with more than just a funnel. From his sectioned material he stated that the anteseptal portion was made up of a few coils of the nephridial canal in addition to the funnel, something we could not make out in our whole-mounted material. Another illustrated nephridium that resembles the one in our species was provided by Finogenova and Timm (1988) who reported *L. semifuscus* from the White Sea (Russia). Their description only differs from that of *C. semifuscoides* in reporting three pairs of pharyngeal glands, with the third pair extending into VII, but this could be a misinterpretation of a fourth pair. As we have not examined their material, we cannot be certain that this is the case, but we find it highly probable that they actually were describing *C. semifuscoides*. Finally, we are not certain about the identity of the species “*Lumbricillus semifuscus*” that Nurminen (1965a) reported from Spitsbergen (Svalbard), as he mentions sperm funnels “considerably longer than 1.5 times the width”.

Compared to the species of *Lumbricillus*, i.e., the genus in which we previously placed this species (and erroneously referred to it as *L. semifuscus*), *C. semifuscoides* can be distinguished mainly by its four pairs of pharyngeal glands, the stalked nephridial funnel, and the irregularly lobed testes.

## Discussion

### General comments on *Lumbricillus* taxonomy

Klinth et al.'s (2017) molecular assessment of *Lumbricillus* was a starting point for the present taxonomic study of the genus, which also has taken the species morphology into account. The *lineatus, pagenstecheri, buelowi* and *arenarius* species groups, which are monophyletic according to Klinth et al. are also supported by consistencies in the morphological characters (Fig. 1, Table 1). The fifth, but non-monophyletic, “*tuba*” group appears morphologically coherent too. In Table 1, 65 additional species of *Lumbricillus* not studied herein, but with some exceptions regarded as valid by Schmelz and Collado (2012), are tentatively classified into these five groups after considering their original descriptions. It is likely that the placement of many of them will be challenged in the future after molecular and morphological examination, and that some of them will even prove to represent lineages of *Lumbricillus* not covered by our sample of carefully studied taxa. Finally, in Table 1, yet five other species with uncertain affinities are listed. The long needed re-assessment of this large genus has just begun.

Diagnosing and delimiting *Lumbricillus* are problematic, as one of its most striking features, the bunch-like arrangement of the lobed testis sacs, is not shared by all species in the genus. This character appears to be a synapomorphy of the *lineatus*, *pagenstecheri* and “*tuba*” groups, which together make up a monophyletic clade, but excludes the *buelowi* and *arenarius* groups, which have unlobed or irregularly lobed testis sacs (Fig. 1). The *buelowi* group has been supported as the sister to the *lineatus*, *pagenstecheri* and “*tuba*” groups, sharing most characters except for the bunch-like arrangement of the testis sacs. The two species in this group studied (*L. buelowi* and *L. knoellneri*) are very small, and it seems that each of their testis sacs comprises a single large lobe, or possibly numerous shallow lobes. However, body size and number of testis lobes do not seem to be functionally correlated because there are other small *Lumbricillus* species, such as *L. pumilio* within the *lineatus* group, which despite their small size have testes with bunch-like arrangement.

Klinth et al. (2017) discussed the possibility of splitting off the *arenarius* group as a separate genus, especially as this group earlier had been suggested as the sister to *Grania* (Erséus et al. 2010). However, the most reliable phylogenetic topology recovered by Klinth et al. (2017) still favored the *arenarius* group as sister to the remaining *Lumbricillus*. Having studied the morphology of the species included in the *arenarius* group we conclude that they resemble *Grania* in some morphological characters, such as non-sigmoid chaetae and few chaetae per bundle. However, in most other characters, first of all the organization of sperm in the spermathecae, *Grania* is clearly different from the *arenarius* group. Furthermore, the *arenarius* group has testes encased in testis sacs, which is only found in *Lumbricillus* and *Enchytraeus* (Stephenson 1930, Tynen et al. 1991), and now also in *Claparedrilus* (present study). Therefore, we propose to keep the three species of the *arenarius* group within *Lumbricillus*, thereby retaining the broad morphological definition of the genus. The different location of the upper chaetal bundles in the *arenarius* and *buelowi* lineages as compared to the other *Lumbricillus* species may simply reflect their adaptation to a mineral, sandy bottom rather than to a loose, water-saturated, organic-rich substrate (for a discussion on this topic see Rota 2001).

### Comments on *Enchytraeoides*

Before we came to the conclusion that the *arenarius* group should remain within *Lumbricillus*, we explored the possibility that it could be treated as a separate genus. Searching the taxonomic literature for possible candidate genera we found *Enchytraeoides*, in which *L. arenarius* had previously been placed by some authors (Ude 1929, Knöllner 1935, von Bülow 1957). Due to the similarity in morphology between *Enchytraeoides* and the *arenarius* group, it is important to determine the proper status of *Enchytraeoides* in relation to *Lumbricillus*.

It is difficult to establish whether *Pachydrilus enchytraeoides* Saint-Loup, 1885 is the same species as *Enchytraeoides marioni* Roule, 1888, but through personal communications with Roule, Delphy (1920) was persuaded that the former had studied the same species, a species that Delphy pointed out should therefore be named *Enchytraeoides enchytraeoides* (more on this in Rota et al. 2008). This taxon, which was described from Marseille, resembles the *arenarius* group in some traits; mainly the irregularly lobed testis sacs, sperm funnels that are several times longer than wide, and spermathecae with what appears to be gradually widening ducts that are difficult to distinguish from the ampulla. However, *E. enchytraeoides* was described as having two to eight chaetae per bundle, which is much more variable than the two to three chaetae per bundle in the *arenarius* group.

Roule described the penial bulbs as situated in segment XI, a trait neither seen in any *Lumbricillus* nor in any other typical enchytraeid in which the bulbs are in XII. Furthermore, there were some discrepancies in Roule’s description as to the location of the spermathecae, which were described as located in segment VI, but were illustrated as in segment V. This could be explained by Roule counting the prostomium as a separate segment, but this still does not explain the placement of the penial bulbs which are also illustrated as part of segment XI. If the material was studied live without proper magnification, and the description and illustration were produced later, this might have caused a misinterpretation of the position of the penial bulbs. However, given the extensive descriptions and illustrations based on such large amounts of material this seems unlikely. Therefore, the true phylogenetic placement of *Enchytraeoides* remains uncertain until newly sampled species have been examined and sequenced and for now it should remain as a junior synonym of *Lumbricillus*.

### Geographical distribution and habitat

It is difficult to make any conclusions of the full geographical distribution of the species in this study, as most of our samples are from Norway and Sweden, with some also from the United Kingdom and other parts of Northern Europe. When taking into account the BOLD data and the reports by other authors it seems that some species are very common, such as *L. lineatus*, *L. rivalis* and *L. pagenstecheri* sensu lato (e.g. Timm 2005). These taxa have been found throughout Europe and in some cases also in North America and the northern Pacific region. Unfortunately, for most reports we do not have DNA sampled specimens to compare with and we are unable to ensure that it is indeed the same species as ours that have been found at some remote location. Fortunately, we could find matching barcoding sequences in the BOLD database for several species that were also collected in Canada, such as *L. lineatus*, *L. rutilus*, *L. rivalis* and *L. pagenstecheri* sensu lato, showing how widespread these species can be and supporting the idea of a partly Holarctic fauna. There are few reports of *Lumbricillus* from tropical areas (possibly due to poor sampling), but species have been reported from the Southern hemisphere, mainly from islands surrounding Antarctica. It would be interesting to compare the genetic information of these species to see if they are parts of, or lineages separate from, the species occurring in the Northern hemisphere. For the *L. pagenstecheri* complex, in which we found support for four different species, our material as well as the many descriptions of species that are very similar to this species seems to indicate that this group is more diverse in the Arctic than it is further south (see for example Shurova 1974, 1977, 1978, 1979).

As evident from Appendix I and the taxonomic literature on *Lumbricillus*, this genus is mostly associated with seashores and brackish waters, but many species (such as *L. arenarius*, *L. fennicus*, *L. knoellneri*, *L. rutilus* and *L. scandicus*) are commonly encountered also in freshwater habitats.

### Future research

Having studied only about one fourth of the 80 or so described species of *Lumbricillus* with a primarily molecular approach, it is clear that a lot of taxonomic and genetic work remains to be done on this genus. Ideally, we would like to be able to link each of the described species to one or more molecular barcodes of COI, and to clearly delimit the species from each other using also nuclear genetic data (as in Klinth et al. 2017). There are several undescribed species left in this genus and we have several unnamed species that could either belong to previously described species or be new to science. A majority of the previously described species identified in our study lack types or other reference material. Thus, a future challenge is to visit type localities of such species, to determine if we truly have the same species or not. Lastly, some groups within *Lumbricillus*, such as *L. pagenstecheri* sensu lato, require some extra attention. For this complex, there are several synonymized names to examine in order to see if they match our specimens. Furthermore, there are many species described from the Pacific coast of Russia (Shurova 1974; 1977; 1978; 1979), and they all seem to share a morphology similar to that of *L. pagenstecheri*, and molecular data will be needed to determine the true number of species in this complex.

## Conclusions

Having studied the morphology of the *Lumbricillus* species included in the molecular study by Klinth et al. (2017) we have found that this genus can be divided into at least five informal subgroups with differing morphology, which may become a useful backbone when resolving the taxonomy and phylogeny of other species in *Lumbricillus*. We propose to keep *L. arenarius*, *L. dubius* and *L.* sp. H within *Lumbricillus* despite their somewhat differing morphology, and their uncertain phylogeny vis-à-vis *Grania*. This means that the morphological characters defining *Lumbricillus* remain quite broad. We have described two new species of *Lumbricillus*: *L. latithecatus* sp. n., which is somewhat reminiscent of *L. lineatus*, and *L. scandicus* sp. n., which was previously thought to be the same as *L. helgolandicus*. *Lumbricillus verrucosus*, which was resurrected from junior synonymy with *L. lineatus* by Klinth et al. (2017), has been given a proper morphological re-description. *Lumbricillus lineatus* remains as the type species for the genus and a neotype has been designated. The genera *Pachydrilus* and *Enchytraeoides* remain as junior synonyms to *Lumbricillus*. Lastly, we have established *Claparedrilus* gen. n., with *C. semifuscoides* sp. n. as the type species, and transferred *Pachydrilus semifuscus* Claparède, a former member of *Lumbricillus*, into the same genus.

## Supplementary Material

XML Treatment for Lumbricillus

XML Treatment for Lumbricillus lineatus

XML Treatment for Lumbricillus rutilus

XML Treatment for Lumbricillus latithecatus

XML Treatment for Lumbricillus verrucosus

XML Treatment for Lumbricillus rivalis

XML Treatment for Lumbricillus

XML Treatment for Lumbricillus kaloensis

XML Treatment for Lumbricillus

XML Treatment for Lumbricillus pumilio

XML Treatment for Lumbricillus rubidus

XML Treatment for Lumbricillus fennicus

XML Treatment for Lumbricillus pagenstecheri

XML Treatment for Lumbricillus pagenstecheri

XML Treatment for Lumbricillus viridis

XML Treatment for Lumbricillus tuba

XML Treatment for Lumbricillus scandicus

XML Treatment for Lumbricillus helgolandicus

XML Treatment for Lumbricillus buelowi

XML Treatment for Lumbricillus knoellneri

XML Treatment for Lumbricillus arenarius

XML Treatment for Lumbricillus

XML Treatment for Lumbricillus dubius

XML Treatment for Claparedrilus

XML Treatment for Claparedrilus semifuscoides

## Appendix 1

List of specimens used in this study, with specimen identification number, collection data, GPS coordinates (in decimal degrees), GenBank accession numbers for the COI barcode, and voucher numbers. Letters for *Lumbricillus pagenstecheri* refer to barcoding clusters. Country codes: ES=Spain, GL=Greenland, NO=Norway, SE=Sweden and UK=United Kingdom.

**Table:**

Species	ID	Collection locality	Coordinates	Date	Leg	Habitat	Barcode Acc. No.	Voucher No.
			Lat. / Long.					
*Claparedrilus semifuscoides* sp n.	CE2247	UK, Wales, Anglesey, Beaumaris	53.2623, -4.0914	15-Feb-2007	M. Strand & P. Sundberg	Intertidal, sand, algae	KU893993	SMNH Type-8933
*Claparedrilus semifuscoides* sp n.	CE2248	UK, Wales, Anglesey, Beaumaris	53.2623, -4.0914	15-Feb-2007	M. Strand & P. Sundberg	Intertidal, sand, algae	KU893994	SMNH Type-8934
*Claparedrilus semifuscoides* sp n.	CE2249	UK, Wales, Anglesey, Beaumaris	53.2623, -4.0914	15-Feb-2007	M. Strand & P. Sundberg	Intertidal, sand, algae	KU893995	SMNH Type-8932
*Claparedrilus semifuscoides* sp n.	CE2252	UK, Wales, Anglesey, Beaumaris	53.2623, -4.0914	15-Feb-2007	M. Strand & P. Sundberg	Intertidal, sand, algae	KU893996	SMNH Type-8935
*Claparedrilus semifuscoides* sp n.	CE23750	NO, Nordland, Bognäs, Tysfjorden	68.2248, 16.0771	17-Aug-2014	C. Erséus	Upper intertidal, seaweeds, stones	KU894097	ZMBN 107908
*Claparedrilus semifuscoides* sp n.	CE24657	NO, Nordland, Lofoten, Grimsöystraumen Strait	68.2607, 14.2679	10-Sep-2014	C. Erséus & E. Willassen	Upper intertidal, coarse shelly sand	KU893989	ZMBN 107912
*Lumbricillus arenarius*	CE1001	SE, Bohuslän, Lysekil, Fiskebäckskil, Kristineberg	58.248, 11.4458	16 May 2005	A. Ansebo	Intertidal shelly sand and gravel	KU893917	SMNH 152716
*L. arenarius*	CE8474	NO, Svalbard, Ny-Ålesund, Kongsfjorden	78.973, 11.4443	21-Jul-2010	D. Fontaneto	Land-locked lagoon	KU894140	ZMBN 107784
*L. arenarius*	CE20748	NO, Svalbard, Spitsbergen, Nordfjorden, Tschermak	78.4903, 15.3289	27-Jul-2013	K. Hårsaker	Freshwater stream, sand	KU893909	ZMBN 107787
*L. arenarius*	CE20749	NO, Svalbard, Spitsbergen, Nordfjorden, Tschermak	78.4903, 15.3289	27-Jul-2013	K. Hårsaker	Freshwater stream, sand	KU893910	ZMBN 107788
*L. arenarius*	CE20750	NO, Svalbard, Spitsbergen, Nordfjorden, Tschermak	78.4903, 15.3289	27-Jul-2013	K. Hårsaker	Freshwater stream, sand	KU893911	ZMBN 107789
*L. buelowi*	CE5224	SE, Bohuslän, Strömstad, Tjärnö	58.8754, 11.1458	8-Oct-2008	C. Erséus	Upper intertidal, sand	KU893945	SMNH 152719
*L. buelowi*	CE22293	NO, Sogn og Fjordane, Neröyfjorden, Bakka	60.9202, 6.8674	15-May-2014	C. Erséus & M. Klinth	Lower intertidal, gravel, sand, algae	KU893941	ZMBN 107802
*L. buelowi*	CE23273	NO, Troms, Tromsö, Tromsö, Lanes	69.629, 18.9207	14-Aug-2014	C. Erséus	Intertidal, sand, gravel	KU894284	ZMBN 107804
*L. buelowi*	CE23375	NO, Troms, Tromsö, Sommaröya, Gurahaugen	69.6321, 18.0278	15-Aug-2014	C. Erséus	Lower intertidal, mixed mineral and shell sand	KU893942	ZMBN 107805
*L. buelowi*	CE23376	NO, Troms, Tromsö, Sommaröya, Gurahaugen	69.6321, 18.0278	15-Aug-2014	C. Erséus	Lower intertidal, mixed mineral and shell sand	KU893943	ZMBN 107806
*L. buelowi*	CE24678	NO, Nordland, Lofoten, Grimsöystraumen Strait	68.2607, 14.2679	10-Sep-2014	C. Erséus & E. Willassen	Upper intertidal, coarse shelly sand	KU893946	ZMBN 107811
*L. buelowi*	CE24688	NO, Nordland, Lofoten, Grimsöystraumen Strait	68.2607, 14.2679	10-Sep-2014	C. Erséus & E. Willassen	Upper intertidal, coarse shelly sand	KU894208	ZMBN 107814
*L. buelowi*	CE24690	NO, Nordland, Lofoten, Grimsöystraumen Strait	68.2607, 14.2679	10-Sep-2014	C. Erséus & E. Willassen	Upper intertidal, coarse shelly sand	KU893947	ZMBN 107816
*L. dubius*	CE5221	SE, Bohuslän, Strömstad, Tjärnö	58.8754, 11.1458	8-Oct-2008	C. Erséus	Upper intertidal, coarse sand	KU893930	SMNH 152726
*L. dubius*	CE5223	SE, Bohuslän, Strömstad, Tjärnö	58.8754, 11.1458	8-Oct-2008	C. Erséus	Upper intertidal, coarse sand	KU893926	SMNH 152727
*L. dubius*	CE22767	NO, Finnmark, Mageröya, Skipsfjorden	71.0093, 25.8933	12-Aug-2014	C. Erséus	Grass at high water mark, roots and sand	KU893931	ZMBN 107835
*L. dubius*	CE23370	NO, Troms, Tromsö, Sommaröya, Gurahaugen	69.6321, 18.0278	15-Aug-2014	C. Erséus	Lower intertidal, mixed mineral and shell sand	KU893934	ZMBN 107836
*L. dubius*	CE23371	NO, Troms, Tromsö, Sommaröya, Gurahaugen	69.6321, 18.0278	15-Aug-2014	C. Erséus	Lower intertidal, mixed mineral and shell sand	KU893932	ZMBN 107837
*L. dubius*	CE24700	NO, Nordland, Lofoten, Grimsöystraumen Strait	68.2607, 14.2679	10-Sep-2014	C. Erséus & E. Willassen	Upper intertidal, coarse shelly sand	KU893928	ZMBN 107839
*L. dubius*	CE24711	NO, Nordland, Lofoten, Grimsöystraumen Strait	68.2607, 14.2679	10-Sep-2014	C. Erséus & E. Willassen	Upper intertidal, coarse shelly sand	KU893929	ZMBN 107840
*L. dubius*	CE24726	NO, Nordland, Lofoten, Grimsöystraumen Strait	68.2608, 14.2678	10-Sep-2014	C. Erséus & E. Willassen	Mid-intertidal, coarse shelly sand, algae	KU893927	ZMBN 107841
*L. fennicus*	CE2767	SE, Öland, Borgholm, S of Föra	56.9855, 16.8764	12-Jun-2007	A. Ansebo, L. Matamoros & C. Erséus	Stream, muddy clay	KU894132	SMNH 152729
*L. fennicus*	CE2768	SE, Öland, Borgholm, S of Föra	56.9855, 16.8764	12-Jun-2007	A. Ansebo, L. Matamoros & C. Erséus	Stream, muddy clay	KU894003	SMNH 152730
*L. fennicus*	CE2988	SE, Öland, Borgholm, S of Föra	56.9855, 16.8764	12-Jun-2007	A. Ansebo, L. Matamoros & C. Erséus	Stream, muddy clay	KU894125	SMNH 152731
*L. fennicus*	CE6092	SE, Bohuslän, Lysekil, Färlev, Färlevsfjorden	58.4785, 11.5722	27-May-2009	C. Erséus, A. Ansebo & M. Johansson	Sand, fine mud	KU894002	SMNH 152732
*L. kaloensis*	CE978	SE, Torslanda, Lilleby, Sillvik	57.7467, 11.755	10-Apr-2005	A. Ansebo	Intertidal sand	KU894128	SMNH 152733
*L. kaloensis*	CE5412	NO, Hordaland, Bergen, Blomsterdalen	60.2689, 5.2212	4-Nov-2011	P. De Wit	Intertidal, sand, rocks	KU894149	ZMBN 107842
*L. knoellneri*	CE980	SE, Västergötland, Göteborg, Fiskebäck	57.6567, 11.8483	15-May-2005	A. Ansebo	Intertidal sand	KU894121	SMNH 152734
*L. knoellneri*	CE982	SE, Västergötland, Göteborg, Fiskebäck	57.6567, 11.8483	15-May-2005	A. Ansebo	Intertidal sand	KU894122	SMNH 152735
*L. knoellneri*	CE19369	NO, Sogn og Fjordane, Luster Nes, Lustrafjorden	61.3864, 7.369	12-Aug-2013	C. Erséus	Upper intertidal, sand	KU893940	ZMBN 107859
*L. knoellneri*	CE20761	NO, Svalbard, Spitsbergen, Nordfjorden, Tschermak	78.4903, 15.3289	27-Jul-2013	K. Hårsaker	Freshwater stream, sand	KU893938	ZMBN 107860
*L. knoellneri*	CE20762	NO, Svalbard, Spitsbergen, Nordfjorden, Tschermak	78.4903, 15.3289	27-Jul-2013	K. Hårsaker	Freshwater stream, sand	KU893939	ZMBN 107861
*L. knoellneri*	CE22615	NO, Finnmark, Porsanger, Roddinessjöen	70.0927, 25.0739	11-Aug-2014	C. Erséus	Intertidal, algae, rubble	KU893937	ZMBN 107863
*L. knoellneri*	CE23252	NO, Troms, Tromsö, Tromsö, Lanes	69.6291, 18.9205	14-Aug-2014	C. Erséus	Supralittoral, seaweed, herbs growing on top	KU894131	ZMBN 107865
*L. knoellneri*	CE23253	NO, Troms, Tromsö, Tromsö, Lanes	69.6291, 18.9205	14-Aug-2014	C. Erséus	Supralittoral, seaweed, herbs growing on top	KU894106	ZMBN 107866
*L. latithecatus* sp. n.	CE1976	SE, Skåne, Höganäs, Mölle	56.2576, 12.5192	29-Jul-2006	C. Erséus	Decaying algae, sand	KU894053	SMNH 152830
*L. latithecatus* sp. n.	CE1979	SE, Skåne, Höganäs, Mölle	56.2576, 12.5192	29-Jul-2006	C. Erséus	Decaying algae, sand	KU894055	SMNH 152831
*L. latithecatus* sp. n.	CE12041	NO, Rogaland, Sola, Ölbörhamna	58.8697, 5.5654	15-Jun-2012	C. Erséus	Intertidal, algae	KU894054	ZMBN 107940
*L. latithecatus* sp. n.	CE12042	NO, Rogaland, Sola, Ölbörhamna	58.8697, 5.5654	15-Jun-2012	C. Erséus	Intertidal, algae	KU894127	ZMBN 107941
*L. lineatus*	CE1640	SE, Bohuslän, Strömstad, Tjärnö	58.8766, 11.1456	1-Sep-2004	C. Erséus	Intertidal, sand, gravel	KU894030	SMNH 152742
*L. lineatus*	CE1694	ES, Galicia, Ponevedra, Illa de Arousa	42.56, -8.87	1-Apr-2006	B. Reboreda Rivera	*Ulva* compost culture	KU894118	SMNH 152744
*L. lineatus*	CE1894	SE, Öland, Mörbylånga, Skärlöv	56.4241, 16.5815	10-Jun-2006	L. Matamoros	Beach, shelley sand, organic material	KU894040	SMNH Type-8931
*L. lineatus*	CE12043	NO, Rogaland, Sola, Ölbörhamna	58.8697, 5.5654	15-Jun-2012	C. Erséus	Intertidal, algae	KU894033	ZMBN 107872
*L. lineatus*	CE21688	NO, Telemark, Kragerö, Gunnarsholmen Island	58.8649, 9.4117	12-May-2014	C. Erséus & M. Klinth	Anaerobic, sand, gravel, 0.3-0.5 m depth.	KU894181	ZMBN 107875
*L. lineatus*	CE21986	NO, Hordaland, Maurangerfjorden, Nedrehus	60.1295, 6.3146	14-May-2014	C. Erséus & M. Klinth	High-water mark, grass roots with sand	KU894177	ZMBN 107878
*L. lineatus*	CE22604	NO, Finnmark, Porsanger, Roddinessjöen	70.0927, 25.0739	11-Aug-2014	C. Erséus	Intertidal, algae, rubble	KU894196	ZMBN 107879
*L. lineatus*	CE22605	NO, Finnmark, Porsanger, Roddinessjöen	70.0927, 25.0739	11-Aug-2014	C. Erséus	Intertidal, algae, rubble	KU894029	ZMBN 107880
*L. pagenstecheri A*	CE1896	SE, Öland, Mörbylånga, Skärlöv	56.4241, 16.5815	10-Jun-2006	L. Matamoros	Beach, shelley sand, organic material	KU893986	SMNH 152766
*L. pagenstecheri A*	CE1897	SE, Öland, Mörbylånga, Skärlöv	56.4241, 16.5815	10-Jun-2006	L. Matamoros	Beach, shelley sand, organic material	KU893987	SMNH 152767
*L. pagenstecheri A*	CE1899	SE, Öland, Mörbylånga, Skärlöv	56.4241, 16.5815	10-Jun-2006	L. Matamoros	Beach, shelley sand, organic material	KU893988	SMNH 152768
*L. pagenstecheri A*	CE2497	SE, St Förö, Västergötland, Göteborg, Stora Förö Island	57.6252, 11.8469	2-Jun-2007	C. Erséus	Supralittoral, algae, gravel and pebbles	-	SMNH 152769
*L. pagenstecheri A*	CE2498	SE, St Förö, Västergötland, Göteborg, Stora Förö Island	57.6252, 11.8469	2-Jun-2007	C. Erséus	Supralittoral, algae, gravel and pebbles	KU894141	SMNH 152770
*L. pagenstecheri A*	CE2500	SE, St Förö, Västergötland, Göteborg, Stora Förö Island	57.62512, 11.8469	2-Jun-2007	C. Erséus	Supralittoral, algae, gravel and pebbles	KU893985	SMNH 152771
*L. pagenstecheri B*	CE22586	NO, Finnmark, Porsanger, Roddinessjöen	70.0927, 25.0739	11-Aug-2014	C. Erséus	Intertidal, algae, rubble	KU893983	ZMBN 107890
*L. pagenstecheri B*	CE22727	NO, Finnmark, Porsanger, Olderfjorden	70.4799, 25.209	12-Aug-2014	C. Erséus	Driftline of dead algae	KU893984	ZMBN 107891
*L. pagenstecheri C*	CE1699	ES, Galicia, Ponevedra, Illa de Arousa	42.56, -8.87	1-Apr-2006	B. Reboreda Rivera	*Ulva* compost culture	KU893981	SMNH 152772
*L. pagenstecheri C*	CE20718	NO, Svalbard, Spitsbergen, Wijdefjorden, Gunvorvatn	79.8186, 15.6646	21-Jul-2013	K. Hårsaker & T. Ekrem	Decaying seaweed	KU893982	ZMBN 107892
*L. pagenstecheri D*	CE22728	NO, Finnmark, Porsanger, Olderfjorden	70.4799, 25.209	12-Aug-2014	C. Erséus	Driftline of dead algae	KU894110	ZMBN 107893
*L. pagenstecheri D*	CE22729	NO, Finnmark, Porsanger, Olderfjorden	70.4799, 25.209	12-Aug-2014	C. Erséus	Driftline of dead algae	KU894210	ZMBN 107894
*L. pagenstecheri D*	CE23482	NO, Nordland, Bjerkvik	68.5481, 17.5422	15-Aug-2014	C. Erséus	Highwater line, sand	KU893980	ZMBN 107895
*L. pagenstecheri D*	SM191	GL, Disko Island, Qeqertarsuaq	69.2489, -53.5437	27-Jul-2013	S. Martinsson	Beach, sand, algae	KU893978	SMNH 152774
*L. pumilio*	CE3346	UK, Wales, Cardiff, Newport, St. Brides	51.517, -3.000	13-Nov-2007	S. Kvist	Supralittoral peat	KU894205	SMNH 152775
*L. pumilio*	CE3347	UK, Wales, Cardiff, Newport, St. Brides	51.517, -3.000	13-Nov-2007	S. Kvist	Supralittoral peat	KU894195	SMNH 152776
*L. pumilio*	CE3427	UK, Wales, Cardiff, Newport, St. Brides	51.517, -3.000	13-Nov-2007	S. Kvist	Supralittoral peat	KU894084	SMNH 152777
*L. pumilio*	CE3428	UK, Wales, Cardiff, Newport, St. Brides	51.517, -3.000	13-Nov-2007	S. Kvist	Supralittoral peat	KU894085	SMNH 152778
*L. pumilio*	CE3430	UK, Wales, Cardiff, Newport, St. Brides	51.517, -3.000	13-Nov-2007	S. Kvist	Supralittoral peat	KU894086	SMNH 152779
*L. pumilio*	CE3436	UK, Wales, Cardiff, Newport, St. Brides	51.517, -3.000	13-Nov-2007	S. Kvist	Supralittoral peat	KU894087	SMNH 152780
*L. pumilio*	CE3437	UK, Wales, Cardiff, Newport, St. Brides	51.517, -3.000	13-Nov-2007	S. Kvist	Supralittoral peat	KU894088	SMNH 152781
*L. rivalis*	CE1873	SE, Blekinge, Sölvesborg, Norje	56.1413, 14.6742	7-Jun-2006	L. Matamoros	Beach, sand, clay	KU894064	SMNH 152782
*L. rivalis*	CE1874	SE, Blekinge, Karlskrona, Torhamn	56.0726, 15.8402	8-Jun-2006	L. Matamoros	Beach, stones, organic material	KU894056	SMNH 152783
*L. rivalis*	CE2503	SE, St Förö, Västergötland, Göteborg, Stora Förö Island	57.6252, 11.8469	2-Jun-2007	C. Erséus	Supralittoral, algae, gravel and pebbles	KU894146	SMNH 152785
*L. rivalis*	CE22596	NO, Finnmark, Porsanger, Roddinessjöen	70.0927, 25.0739	11-Aug-2014	C. Erséus	Intertidal, algae, rubble	KU894103	ZMBN 107897
*L. rivalis*	CE22600	NO, Finnmark, Porsanger, Roddinessjöen	70.0927, 25.0739	11-Aug-2014	C. Erséus	Intertidal, algae, rubble	KU894112	ZMBN 107898
*L. rivalis*	CE22602	NO, Finnmark, Porsanger, Roddinessjöen	70.0927, 25.0739	11-Aug-2014	C. Erséus	Intertidal, algae, rubble	KU894102	ZMBN 107899
*L. rubidus*	CE2549	SE, Saltholmen, Västergötland, Göteborg, Saltholmen	57.6631, 11.8516	7-Jun-2007	C. Erséus	Supralittoral, roots and brown soil	KU894094	SMNH 152792
*L. rubidus*	CE2551	SE, Saltholmen, Västergötland, Göteborg, Saltholmen	57.6631, 11.8516	7-Jun-2007	C. Erséus	Supralittoral, roots and brown soil	KU894153	SMNH 152793
*L. rubidus*	CE2553	SE, Saltholmen, Västergötland, Göteborg, Saltholmen	57.6631, 11.8516	7-Jun-2007	C. Erséus	Supralittoral, roots and brown soil	KU894152	SMNH 152794
*L. rubidus*	CE6105	SE, Bohuslän, Lysekil, Färlev, Färlevsfjorden	58.4765, 11.576	27-May-2009	C. Erséus, A. Ansebo & M. Johansson	Sand and clay	KU894091	SMNH 152795
*L. rubidus*	CE6106	SE, Bohuslän, Lysekil, Färlev, Färlevsfjorden	58.4765, 11.576	27-May-2009	C. Erséus, A. Ansebo & M. Johansson	Sand and clay	KU894092	SMNH 152796
*L. rubidus*	CE6107	SE, Bohuslän, Lysekil, Färlev, Färlevsfjorden	58.4765, 11.576	27-May-2009	C. Erséus, A. Ansebo & M. Johansson	Sand and clay	KU894089	SMNH 152797
*L. rubidus*	CE6108	SE, Bohuslän, Lysekil, Färlev, Färlevsfjorden	58.4765, 11.576	27-May-2009	C. Erséus, A. Ansebo & M. Johansson	Sand and clay	KU894150	SMNH 152798
*L. rutilus*	CE1887	SE, Blekinge, Karlskrona, Torhamn	56.0723, 15.8402	8-Jun-2006	L. Matamoros	Organic sediment	KU894115	SMNH 152801
*L. rutilus*	CE1903	SE, Öland, Borgholm, Neptuni Åkrar	57.3346, 17.0102	11-Jun-2006	L. Matamoros	Sand, shells, org. material	KU894013	SMNH 152802
*L. rutilus*	CE2510	SE, St Förö, Västergötland, Göteborg, Stora Förö Island	57.6252, 11.8469	2-Jun-2007	C. Erséus	Supralittoral, algae, gravel and pebbles	KU894004	SMNH 152804
*L. rutilus*	CE2937	SE, Öland, Mörbylånga, Sandbyborg	56.5536, 16.6394	13-Jun-2007	A. Ansebo, L. Matamoros & C. Erséus	Seashore, sand, organic material, 0.1 m deep in sand	KU894203	SMNH 152809
*L. rutilus*	CE2939	SE, Öland, Mörbylånga, Sandbyborg	56.5536, 16.6394	13-Jun-2007	A. Ansebo, L. Matamoros & C. Erséus	Seashore, sand, organic material, 0.1 m deep in sand	KU894011	SMNH 152811
*L. rutilus*	CE3060	SE, Västergötland, Göteborg, Ryaverket	57.6974, 11.8928	15-Aug-2007	R. Almstrand	Sewage treatment plant	KU894006	SMNH 152813
*L. rutilus*	CE3061	SE, Västergötland, Göteborg, Ryaverket	57.6974, 11.8928	15-Aug-2007	R. Almstrand	Sewage treatment plant	KU894007	SMNH 152814
*L. rutilus*	CE3502	UK, Manchester, Urmston, United Utilities, Davyhulme Wastewater Treatment Works	53.4639, -2.3741	10-Feb-2008	M. Dempsey	Biofilter (indoors)	KU894008	SMNH 152815
*L. rutilus*	CE3506	UK, Manchester, Urmston, United Utilities, Davyhulme Wastewater Treatment Works	53.4639, -2.3741	10-Feb-2008	M. Dempsey	Biofilter (indoors)	KU894126	SMNH 152816
*L. rutilus*	CE9267	SE, Gotland, Visby, Palissaderna Park	57.6346, 18.284	7-Aug-2010	C. Erséus	Freshwater	KU894142	SMNH 152819
*L. scandicus* sp. n.	CE975	SE, Torslanda, Lilleby, Sillvik	57.7467, 11.755	10-Apr-2005	A. Ansebo	Intertidal sand	KU893954	SMNH 152720
*L. scandicus* sp. n.	CE1905	SE, Öland, Borgholm, Neptuni Åkrar	57.3346, 17.0102	11-Jun-2006	L. Matamoros	Sand, shells, org. material	KU893950	SMNH Type-8923
*L. scandicus* sp. n.	CE1907	SE, Öland, Borgholm, Neptuni Åkrar	57.3346, 17.0102	11-Jun-2006	L. Matamoros	Sand, shells, org. material	KU893951	SMNH Type-8925
*L. scandicus* sp. n.	CE1915	SE, Öland, Borgholm, Ölands Norra Udde	57.3681, 17.0918	11-Jun-2006	L. Matamoros	Beach, sand, stones	KU893952	SMNH 152723
*L. scandicus* sp. n.	CE2548	SE, Saltholmen, Västergötland, Göteborg, Saltholmen	57.6631, 11.8516	7-Jun-2007	C. Erséus	Supralittoral, roots and brown soil	KU893953	SMNH 152724
*L. scandicus* sp. n.	CE2552	SE, Saltholmen, Västergötland, Göteborg, Saltholmen	57.6631, 11.8516	7-Jun-2007	C. Erséus	Supralittoral, roots and brown soil	KU893955	SMNH 152725
*L. tuba*	CE22614	NO, Finnmark, Porsanger, Roddinessjöen	70.0927, 25.0739	11-Aug-2014	C. Erséus	Intertidal, algae, rubble	KU894207	ZMBN 107916
*L. verrucosus*	CE968	SE, Torslanda, Lilleby, Sillvik	57.7467, 11.755	10-Apr-2005	A. Ansebo	Intertidal sand	KU894068	SMNH 152826
*L. verrucosus*	CE21479	NO, Vestfold, Larvik, Jordfallen	59.0427, 10.0174	12-May-2014	C. Erséus & M. Klinth	Subtidal, sand, gravel	KU894193	ZMBN 107919
*L. verrucosus*	CE21486	NO, Vestfold, Larvik, Jordfallen	59.0427, 10.0174	12-May-2014	C. Erséus & M. Klinth	Subtidal, sand, gravel	KU894186	ZMBN 107920
*L. verrucosus*	CE21490	NO, Vestfold, Larvik, Jordfallen	59.0427, 10.0174	12-May-2014	C. Erséus & M. Klinth	Subtidal, sand, gravel	KU894184	ZMBN 107921
*L. verrucosus*	CE21494	NO, Vestfold, Larvik, Jordfallen	59.0427, 10.0174	12-May-2014	C. Erséus & M. Klinth	Subtidal, sand, gravel	KU894067	ZMBN 107922
*L. verrucosus*	CE21811	NO, Vest-Agder, Lyngdal, Lene, Lenefjorden	58.1353, 7.1817	13-May-2014	C. Erséus & M. Klinth	Subtidal, sulfidic gravel	KU894071	ZMBN 107924
*L. verrucosus*	CE21816	NO, Vest-Agder, Lyngdal, Lene, Lenefjorden	58.1353, 7.1817	13-May-2014	C. Erséus & M. Klinth	Subtidal, sulfidic gravel	KU894114	ZMBN 107925
*L. verrucosus*	CE21821	NO, Vest-Agder, Lyngdal, Lene, Lenefjorden	58.1353, 7.1817	13-May-2014	C. Erséus & M. Klinth	Subtidal, sulfidic gravel	KU894073	ZMBN 107926
*L. viridis*	CE12037	NO, Rogaland, Sola, Ölbörhamna	58.8697, 5.5654	15-Jun-2012	C. Erséus	Intertidal, decomp. algae	KU893969	ZMBN 107933
*L. viridis*	CE12038	NO, Rogaland, Sola, Ölbörhamna	58.8697, 5.5654	15-Jun-2012	C. Erséus	Intertidal, decomp. algae	KU893970	ZMBN 107934
*L. viridis*	CE12039	NO, Rogaland, Sola, Ölbörhamna	58.8697, 5.5654	15-Jun-2012	C. Erséus	Intertidal, decomp. algae	KU893971	ZMBN 107935
*L. viridis*	CE23255	NO, Troms, Tromsö, Tromsö, Lanes	69.629, 18.9207	14-Aug-2014	C. Erséus	Intertidal, sand, gravel	KU893967	ZMBN 107938
*L. sp. F*	CE2659	UK, Devon, Plymouth, River Plym	50.3757, -4.1082	20-Jul-2007	L. Matamoros	Iintertidal, stones, clay	KU893997	SMNH 152832
*L. sp. G*	CE2246	UK, Wales, Anglesey, Beaumaris	53.2623, -4.0914	15-Feb-2007	M. Strand & P. Sundberg	Intertidal, sand, algae	KU894001	SMNH 152834
*L. sp. G*	CE2661	UK, Devon, Plymouth, River Plym	50.3757, -4.1082	20-Jul-2007	L. Matamoros	Iintertidal, stones, clay	KU893999	SMNH 152835
*L. sp. G*	CE23373	NO, Troms, Tromsö, Sommaröya, Gurahaugen	69.6321, 18.0278	15-Aug-2014	C. Erséus	Lower intertidal, mixed mineral and shell sand	KU894000	ZMBN 107942
*L. sp. H*	CE23136	NO, Troms, Rotsundselv	69.8001, 20.7158	14-Aug-2014	C. Erséus	Upper intertidal, sand	KU893920	ZMBN 107945
*L. sp. H*	CE24967	NO, Nordland, Gildeskål, Kjöpstad, Holmsundsfjorden Bay	67.0536, 14.2729	11-Sep-2014	C. Erséus & E. Willassen	Upper intertidal, rock pool, gravel, sand	KU893921	ZMBN 107947
*L. sp. H*	CE24968	NO, Nordland, Gildeskål, Kjöpstad, Holmsundsfjorden Bay	67.0536, 14.2729	11-Sep-2014	C. Erséus & E. Willassen	Upper intertidal, rock pool, gravel, sand	KU893919	ZMBN 107948
